# The Goblet Cell Protein Clca1 (Alias mClca3 or Gob-5) Is Not Required for Intestinal Mucus Synthesis, Structure and Barrier Function in Naive or DSS-Challenged Mice

**DOI:** 10.1371/journal.pone.0131991

**Published:** 2015-07-10

**Authors:** Nancy A. Erickson, Elisabeth E. L. Nyström, Lars Mundhenk, Liisa Arike, Rainer Glauben, Markus M. Heimesaat, André Fischer, Stefan Bereswill, George M. H. Birchenough, Achim D. Gruber, Malin E. V. Johansson

**Affiliations:** 1 Department of Veterinary Pathology, Freie Universität Berlin, Berlin, Germany; 2 Department of Medical Biochemistry, University of Gothenburg, Gothenburg, Sweden; 3 Medical Department, Division of Gastroenterology, Infectiology and Rheumatology—Charité, Universitätsmedizin Berlin, Berlin, Germany; 4 Department of Microbiology and Hygiene, Charité - Universitätsmedizin Berlin, Berlin, Germany; French National Centre for Scientific Research, FRANCE

## Abstract

The secreted, goblet cell-derived protein Clca1 (chloride channel regulator, calcium-activated-1) has been linked to diseases with mucus overproduction, including asthma and cystic fibrosis. In the intestine Clca1 is found in the mucus with an abundance and expression pattern similar to Muc2, the major structural mucus component. We hypothesized that Clca1 is required for the synthesis, structure or barrier function of intestinal mucus and therefore compared wild type and *Clca1*-deficient mice under naive and at various time points of DSS (dextran sodium sulfate)-challenged conditions. The mucus phenotype in *Clca1*-deficient compared to wild type mice was systematically characterized by assessment of the mucus protein composition using proteomics, immunofluorescence and expression analysis of selected mucin genes on mRNA level. Mucus barrier integrity was assessed *in-vivo* by analysis of bacterial penetration into the mucus and translocation into sentinel organs combined analysis of the fecal microbiota and *ex-vivo* by assessment of mucus penetrability using beads. All of these assays revealed no relevant differences between wild type and *Clca1*-deficient mice under steady state or DSS-challenged conditions in mouse colon. Clca1 is not required for mucus synthesis, structure and barrier function in the murine colon.

## Introduction

The goblet-cell derived protein Clca1 (chloride channel regulator, calcium-activated-1, previously termed mClca3 or gob-5, see revised nomenclature S1 Table) has repeatedly been linked to several human respiratory conditions such as asthma, cystic fibrosis (CF) and chronic obstructive pulmonary disease (COPD) as well as to corresponding mouse models [1–4]. Common features related to disease severity are goblet cell hyperplasia, mucus overproduction and disturbed clearance, together with Clca1 protein and mRNA overexpression which has led to the conclusion that Clca1 is involved in mucin gene expression and/or altering mucus properties [4]. Experimental overexpression of murine Clca1 aggravates the asthma phenotype in ovalbumin- or interleukin (IL)-9-challenged mouse models [5,6]. Conversely, neutralization via antisense- or antibody-treatment of Clca1 ameliorates disease severities [5,7].

Indeed, it was recently shown that the human CLCA1 induces airway goblet cell metaplasia via an extracellular MAPK (mitogen-activated protein kinase)-signaling pathway that drives mucus gene expression [8]. Therefore, CLCA1 may be key to the pathogenesis of conditions with mucus overproduction, possibly suggesting a role in novel therapeutic concepts [4]. However, the function of CLCA1 is still far from being understood.

CLCA1 is abundantly secreted by goblet cells of the intestinal tract together with the MUC2 mucin [9–13]. MUC2 is a highly glycosylated, multimerizing protein and the main structural mucus component, creating net-like sheets that stack upon each other after secretion, thus forming a sieving mucus structure that is normally impenetrable to bacteria [11,14]. It thereby acts as a protective gel, providing an effective barrier against the commensal colonic bacteria [11,15]. In patients with ulcerative colitis, there is strong evidence for mucus layer disruption contributing to the disease pathogenesis [16]. Also, experimentally altered mucus properties consistently induce colitis in mice due to breakdown of the intestinal mucus barrier [16–18].

Several other proteins have been identified in the mucus which are thought to also contribute to mucus structure formation [11,13]. One of these is Clca1, a major constituent of the extracellular intestinal mucus, with a similar expression pattern to Muc2 along the intestinal axis [13]. During its biosynthesis, the primary Clca1 translation product is autocatalytically cleaved within its carboxy-terminal region into two secreted cleavage products by a self-contained zinc-dependent metallohydrolase [19–22]. Such metallohydrolases are thought to critically alter mucus properties [23]. Additionally, a conserved von Willebrand factor type A (vWA) domain with a metal-dependent adhesion site is contained in the amino-terminal cleavage product of CLCA1 which implies that it may interact with other proteins [20,24,25], supporting the notion of a structural role in mucus formation. Furthermore, restoration of decreased Clca1 protein levels in a mouse model of CF has been shown to ameliorate intestinal mucus plugging which is a common trait of human and murine CF [23,26]. It has consequently been hypothesized that CLCA1 may play a role in mucus formation [5,10,21,24,27].

In this study we characterized the intestinal mucus phenotype and microbiota composition of wild type (WT) versus *Clca1*-deficient (*Clca1* ^-/-^) mice under naive conditions, in a set of *ex vivo* experiments and under conditions of DSS (dextran sodium sulfate) challenge. DSS destabilizes biophysical mucus layer properties but is thought not to affect mucin biosynthesis [28]. It is commonly used to experimentally induce colitis via its rapid deleterious effects on mucus integrity which causes mucus barrier disruption and allows bacterial penetration and subsequent epithelial barrier disruption [28,29]. The consequent inflammatory response results in clinically manifest colitis after a treatment course of several days that in many ways resemble and can be used as a model for Ulcerative colitis [29]. Here, mucus barrier integrity was also determined by quantification of bacterial translocation into sentinel organs. Furthermore, the intestinal microbiota composition was analyzed in terms of main gut bacterial groups since these are known to be sensitively altered during development of experimental colitis [30,31].

Much to our surprise, we failed to detect any differences in all of our experimental readout parameters between *Clca1* ^-/-^ and wild type mice, regardless of whether unchallenged or DSS-challenged tissues were employed.

## Materials and Methods

### Ethics statement, mice and DSS treatment

All animal studies involving naive mice were conducted at the Department of Medical Biochemistry, University of Gothenburg, Sweden, in full compliance with Swedish animal welfare legislation and approved by the Swedish Laboratory Animal Ethical Committee in Gothenburg, Sweden (number: 280–2012). Animal studies also involving DSS treatment were conducted at the Research Institutes for Experimental Medicine, Charité - Universitätsmedizin Berlin, Campus Benjamin Franklin, Germany, in strict accordance with the FELASA guidelines and recommendations for the care and use of laboratory animals [32] and approved by the local governmental authorities (State Office of Health and Social Affairs Berlin, approval IDs: T 0394/12; G 0170/12).

Naive *Clca1* ^-/-^ mice, generated on a C57BL/6J background [33] and control mice of the same genetic background were allowed an acclimatization period of at least 10 days and housed under standardized SPF conditions. Animals of both genders between 6 and 12 weeks of age were used, except for DSS experiments involving only weight- and age-matched female mice. For experimental procedures, mice were given 2.5% DSS (MW 36,000–50,000, 17.1% sulfur substitution, no detectable free sulfate, pH 7.1, MP Biomedicals, LLC., Illkirch, France) in their drinking water for 24 (24 h-group) or 48 (48 h-group) hours to allow for the investigation of initial mucus barrier disruptions. In addition, DSS administration for 7 days with 2 consecutive days of tap water only (7 d-group) allowed the monitoring of effects with a completely disrupted barrier and assessment of clinical parameters in terms of fecal blood content [34], using the hemoCARE Guaiac testing method (CARE diagnostic, Voerde, Germany) and stool consistency [35]. All mice were anesthetized with isoflurane prior to euthanasia by cervical dislocation.

### Immunofluorescence

Intestinal tissues were fixed in Methanol-Carnoy and immunostained as described before [36]. Sections were immunostained with a rabbit anti-MUC2-C3 antibody (1:500) [11] and co-stained with a mouse anti-human CLCA1 antibody (Abcam, Cambridge, UK, ab129283, 1:1,000). Goat anti-rabbit IgG conjugated to Alexa Fluor 488 (Molecular Probes, Thermo Fisher, Waltham, MA USA, 1:1,000), or goat anti-mouse IgG conjugated to Alexa Fluor 546 (Molecular Probes, Thermo Fisher, 1:1,000) were used as secondary antibodies, respectively. WT and *Clca1* ^*-/-*^ intestinal sections were stained with a rabbit anti-mouse CLCA1 antibody (amino-terminal reactive; Abcam ab46512, 1:1,000) or a mouse anti-human CLCA1 antibody (carboxy-terminal reactive; Abcam ab129283, 1:2,000) combined with an appropriate Alexa Fluor 546-conjugated secondary antibody to verify the antibody specificity. Other primary antibodies used were: goat anti-Agr2 (Santa Cruz Biotechnology, Dallas, USA, sc-54561, 1:100), rabbit anti-Fcgbp (Sigma-Aldrich, HPA003517, 1:100), rabbit anti-Klk1 (Boster Immunoleader, Pleasanton, CA, PA1709, 1:1,000) or rabbit anti-Zg16 (made in house, PH8292B-11, 1:600) [13] followed by the appropriate Alexa Fluor 546-conjugated secondary antibodies. DNA was stained for 5 min using 1 μg/ml Hoechst 34580 (Molecular Probes, Thermo Fisher). Micrographs were obtained using a Nikon eclipse E1000 fluorescence microscope (Nikon, Tokyo, Japan) with the NIS elements software (Nikon) or a LSM700 Axio Examiner Z.1 confocal microscope (Zeiss, Göttingen, Germany) with the ZEN 2010 software (Zeiss).

### Proteomics

#### Sample collection

Mucus samples were collected from flushed colonic tissue of WT and *Clca1* ^*-/-*^ mice mounted in a horizontal perfusion chamber as described for mucus thickness measurements below but without removal of the muscle layer. 10 μm polystyrene beads (FluoSpheres, Molecular Probes, Thermo Fisher) were used to visualize the mucus layer. Mucus was gently scraped off the epithelium into the apical buffer and was collected using a micropipette [37]. 2x cOmplete EDTA (ethylenediaminetetraacetic acid)-free protease inhibitor (Roche, Mannheim, Germany) was added to the samples.

#### Sample preparation

Mucus samples were incubated overnight at 37°C in reduction buffer (6 M GuHCl, 0.1 M Tris/ HCl pH 8.5 (Merck), 5 mM EDTA, 0.1 M DTT (Merck)) followed by a FASP (Filter-aided Sample Prep) digestion protocol [38] using 6 M guanidinium hydrochloride (GuHCl) instead of urea. Briefly, proteins were alkylated on 30 kDa cut-off filters and subsequently digested for 4 h with LysC (Wako, Richmond, VA, USA) followed by an overnight trypsin (Promega, Fitchburg, WI, USA) digestion. Peptides released from the filter after centrifugation were cleaned with StageTip C18 columns as previously described [13,39].

#### Nano-Liquid-Chromatography Mass-spectrometry (NanoLC–MS/MS)

NanoLC–MS/MS was performed on an EASY-nLC system (Thermo Scientific, Odense, Denmark), connected to a Q Exactive Hybrid Quadrupole-Orbitrap Mass Spectrometer (Thermo Scientific) through a nanoelectrospray ion source. Peptides were separated with reverse-phase column (150 × 0.075 mm inner diameter, C18-AQ 3 μm) by 95-minute gradient from 5 to 30% B A (A: 0.1% formic acid, B: 0.1% formic acid/80% acetonitrile 200 nl/min). Full mass spectra were acquired from 350–1,600 m/z with resolution of 70,000 (m/z 200). Up to twelve most intense peaks (charge state ≥ 2) were fragmented and tandem mass spectrum was acquired with a resolution of 35,000 and dynamic exclusion 30 s.

#### Data analysis

Data was analyzed with the MaxQuant program (version 1.4.1.2 [40]), as previously described [13].

Label-free quantification was performed by loading LFQ-intensity data from the MaxQuant output to the Perseus software (version 1.4.1.3). The data was filtered based on the presence of a protein group in at least four out of five samples in at least one group. Each group (WT and *Clca1* ^*-/-*^mice) was averaged using the median with the median values being presented in a scatterplot.

The mass spectrometry proteomics data were deposited to the ProteomeXchange Consortium (http://proteomecentral.proteomexchange.org) via the PRIDE partner repository [41] with the dataset identifier PXD001804.

### Mucus thickness measurements and responsiveness to induced secretion

Procedure, material and equipment were employed as previously described [37]. Briefly, the distal colon was dissected and thoroughly flushed with ice-cold, oxygenated Krebs’ solution. It was then opened up and the longitudinal muscle layer removed before being mounted in a 2.5 mm horizontal perfusion chamber with oxygenated basolateral Krebs’ glucose solution and apical Krebs’ mannitol solution. Activated charcoal particles (Fluka) were added to the apical surface to visualize the mucus layer. The mucus thickness was measured every 15 min for 1 h after an initial measurement at time 0 h (t0). To investigate the responsiveness of the tissue to induce mucus secretion, carbachol (CCh, 1mM, Sigma Aldrich) was added to the basolateral buffer after 30 min in one of the two specimen-containing chambers.

### Mucus penetrability assay

The mucus penetrability assay was performed on distal colon tissue as previously described [16,37]. Briefly, the colonic explants were mounted in the perfusion chamber with basolateral Kreb’s glucose solution containing 1μg/ml Calcein violet tissue stain (CellTrace Calcein Violet, Molecular Probes, Thermo Fisher) and a suspension of far red fluorescent beads (FluoSpheres Carboxylate-Modified Microspheres, 1.0 μm, red fluorescent (580/605), 2% solids, Molecular Probes, Thermo Fisher) and Kreb’s mannitol solution was added to the apical side and allowed to sediment for 5 min before it was refilled with Kreb’s mannitol solution. The distribution of the beads in the mucus was investigated by acquiring confocal images in XY stacks (320 x 320 μm) 30min after tissue mounting. These were obtained using a LSM700 Axio Examiner Z.1 confocal microscope with Plan-Apochromat x 20/1.0 DIC water objective (Zeiss) and the ZEN 2010 software (Zeiss). Average bead distance from the tissue surface was calculated by combining bead fluorescence intensity data for each z-plane above the tissue surface.

### Fluorescence-*in situ*-hybridization

This was performed as previously described [36]. In brief, de-waxed and washed Methanol-Carnoy fixed tissues were hybridized with the general bacterial probe, EUB338-I (250 ng, 5’-GCT GCC TCC CGT AGG AGT-3’, Eurofins genomics, Ebersberg, Germany), conjugated to Alexa Fluor 555 (Molecular Probes, Thermo Fisher) [11,36]. For mucus visualization slides were incubated with a rabbit anti-MUC2-C3 antibody (1:500) followed by incubation with goat anti-rabbit IgG conjugated to Alexa Fluor 488 (1:1000, Molecular Probes, Thermo Fisher). Nuclei were stained with Hoechst 34580 (Molecular Probes, Thermo Fisher) at 1 μg/ml in PBS. Slides were dried and cover-slipped with ProLong Antifade (Molecular Probes, Thermo Fisher). Micrographs were obtained using an Eclipse E1000 (Nikon) fluorescence microscope with the NIS element software (Nikon).

### Mucus penetration, mucus layering and goblet cell filling score

Bacterial penetration of the inner mucus layer was scored using the fluorescence-*in situ*-hybridization (FISH)-, anti-Muc2- and DNA-stained sections as described previously (S2 Table) [28]. The mucus layering score, focusing solely on the layering and structure of the inner, stratified mucus layer and the goblet cell filling score [16] were applied (S3 Table and S4 Table, respectively). Sections were scored independently by two blinded individuals, except for the naive controls which were scored by one blinded individual using the Eclipse E1000 and Eclipse90i (Nikon) fluorescence microscope as well as the Axiovert 200M microscope (Zeiss) with the EXFO X-Cite 120 Fluorescence Illumination System (Olympus, Hamburg, Germany). Micrographs were obtained using the LSM700 Axio Examiner Z.1 confocal microscope (Zeiss) with the ZEN 2010 software (Zeiss).

### Bacterial translocation

Blood derived via sterile cardiac puncture and biopsies of spleen, liver and mesenteric lymphnodes (MLN) draining the large intestine were taken from WT and *Clca1* ^*-/-*^ controls under naive conditions as well as after DSS challenge (7 d-group; n = 10 per group) and cultivated in thioglycolate enrichment broths (Thioglycolat-Bouillon, Oxoid, Wesel, Germany) as described [42]. Bacterial growth was monitored daily by turbidity assessment. Aliquots from turbid broths were cultivated on solid media under aerobic, microaerophilic and obligate anaerobic conditions and the bacterial species were identified microbiologically and biochemically as described earlier [43].

### Fecal microbiota composition—Real-time PCR and quantification of 16S rRNA gene sequences

Real-time PCR-based quantification of main gut bacterial groups—*Enterobacteriaceae*, Enterococci, lactic acid bacteria, bifidobacteria, *Bacteroides/ Prevotella spp*., *Clostridium leptum* group, *Clostridium coccoides* group, mouse intestinal bacteroidetes and total eubacterial load—was performed.

DNA was extracted from colonic fecal samples as described previously (Heimesaat 2006) with minor modifications. Here, the sediment was incubated with 50 μl of lysozyme (20 mg/ml) and proteinase K (20 mg/ml; Sigma-Aldrich) for 30 min at 37°C and resuspended with 0.5 ml of lysis buffer (0.01 M Tris pH 9.0, 0.02 M EDTA, 0.15 M NaCl, 0.5% SDS).

Total DNA was quantified via Quant-iT PicoGreen dsDNA reagent (Invitrogen, Paisley, UK) as described [44] and adjusted to 10 ng DNA/μl. The abundance of specific intestinal bacterial groups was measured by quantitative real-time PCR as described [42,44–46]. Targets and 16S rRNA gene group-specific primers are listed in S5 Table. Data are expressed as median in a scatterplot. A minimum cut-off value of 10^0^ gene copy numbers/ng DNA and a minimum log-change of 1 were set as limits of statistically significant relevance with p < 0.05.

### RNA isolation and quantitative reverse transcriptase-qPCR

Sections of the distal colon were opened longitudinally, flushed briefly in ice-cold PBS before snap freezing in liquid nitrogen for subsequent storage at -80°C. Total RNA isolation using the Nucleo Spin RNA isolation Kit (Macherey Nagel, Düren, Germany), primer and probe design, quantitative Reverse Transcriptase-PCR (RT-qPCR) protocols, RT-qPCR and data analysis was performed as previously described [47].

Transcript expression levels of murine *Muc1*, -*2*, -*3*, -*4*, -*5ac*, -*5b* and *Muc6* were determined and normalized to the internal reference genes glyceraldehyde-3-phosphate dehydrogenase (*Gapdh*), elongationfactor 1α (*Ef-1α*) and ß-2 microglobulin (*B2m*) as previously described [47]. Primers and probes for *Ef-1α* [48], *Gapdh* [49], *B2m* [50], *Muc4* [51] and *Muc5ac* [52] were used as described. Primers and probes for *Muc1*, *-2*, *-3*, *-5b* and *-6* were designed using Primer3 software (WWW primertool, Whitehead Institute of Biomedical Research). Primer and probe sequences are listed in S6 Table.

The ΔΔCt method based on data obtained from naive WT control animals as reference allowed for relative expression level quantification and group comparison. Significance levels (p-values) were determined by Mann-Whitney-U test. Data are expressed as fold changes. Fold changes of 0.5 and 2 are considered as limits for valid statement of lowered and elevated parameters, respectively.

### Statistics

Data are expressed as mean ± SEM, except for the proteomics log2 intensity, fecal microbiota composition and gene expression level data which are expressed as indicated. Pearson's correlation coefficient and the fraction of shared variance, R squared, were calculated for mucus proteomics data. Statistical analysis of mucus thickness and growth was performed with 2-way ANOVA followed by Tukey’s HSD test for multiple comparisons. Impenetrable mucus thickness was compared by unpaired two-tailed t-test with 95% confidence interval. Other statistical analyses were performed using the Mann-Whitney-U test. P < 0.05 was considered significant. Statistical analysis and graphical illustrations were performed using GraphPad PRISM 6 (GraphPad Software Inc., La Jolla, USA).

## Results

### Expression of the major intestinal mucin Muc2 and mucus structure are not altered in *Clca1* ^-/-^ mice

**Fig 1:**
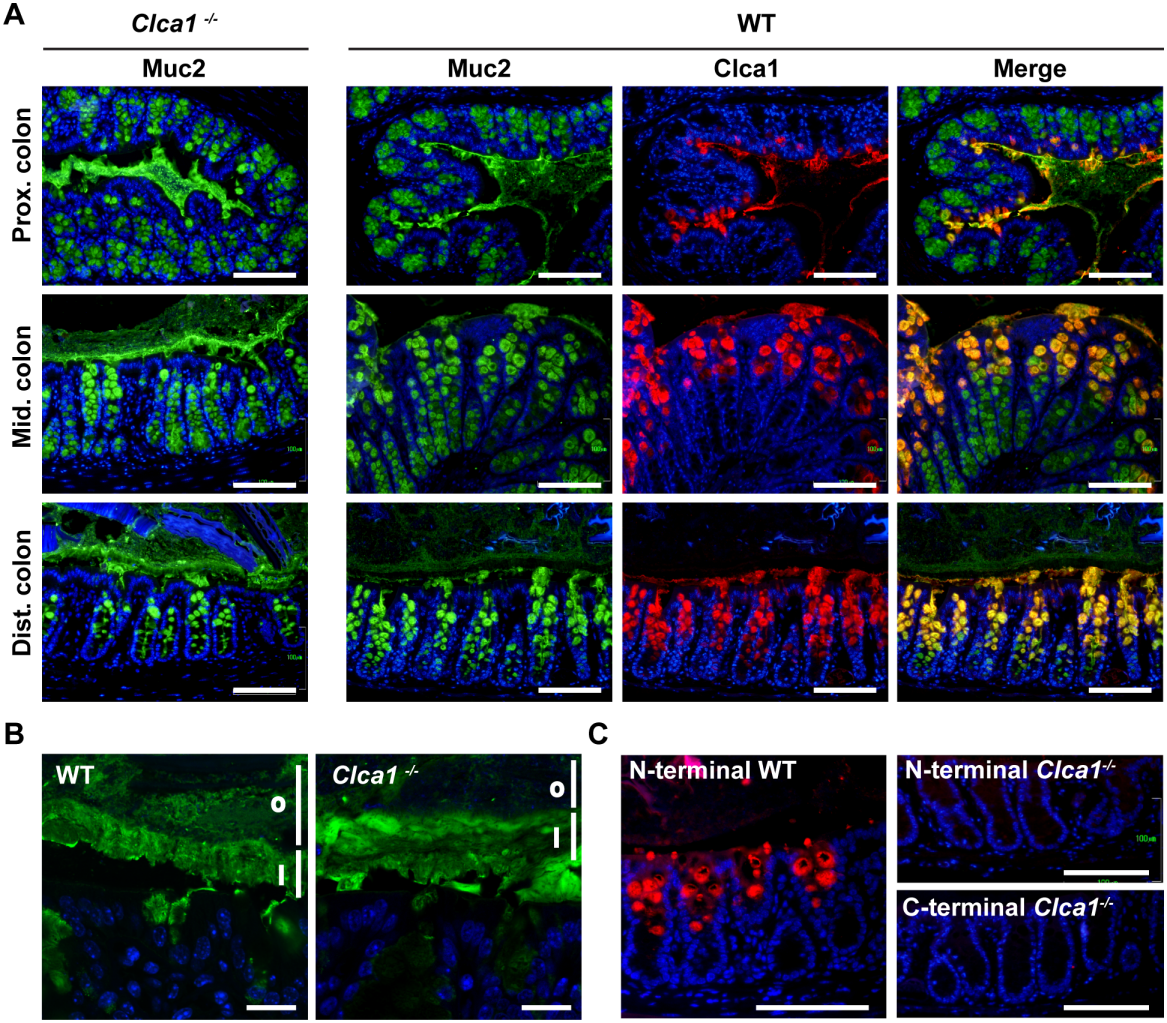
No difference of Muc2 expression pattern was observed by immunofluorescence in WT and *Clca1* ^*-/-*^ mice. (A) The expression patterns of Muc2 (green) were investigated in the large intestine in *Clca1* ^*-/-*^ mice and WT mice as control. No difference in Muc2 expression pattern could be detected between WT and *Clca1* ^*-/-*^ mice in any location. Co-expression of Clca1 and Muc2 were confirmed by co-staining (yellow, merge with Clca1 carboxy-terminus stained red). The highest expression of CLCA1 was found in distal colon of WT mice and was restricted to goblet cells of the upper parts of the crypts. (B) Higher magnification revealed a stratified inner mucus layer by Muc2 staining (green) in both WT and *Clca1* ^*-/-*^ distal colon sections. i = inner mucus layer, o = outer mucus layer. (C) Primary antibodies directed against either the amino- or carboxy-terminus of Clca1 (red) were used to confirm the absence of murine Clca1 expression in the distal colon of *Clca1* ^*-/-*^ mice. All sections were stained with Hoechst 34580 DNA stain (blue), n = 4 per group. Scale bars in A and C 100 μm, in B 20 μm.

Previous studies have identified the highest levels of murine Clca1 expression as being located in the intestinal tract where it is restricted to mucin producing goblet cells in the upper half of the colonic crypts and to villus goblet cells of the small intestine [10,13]. In order to investigate if Clca1 deficiency affected the Muc2 expression pattern or mucus structure in the large intestine, immunofluorescence (IF) using an anti-MUC2-C3 antibody was performed on sections from proximal, mid and distal colon. The anti-MUC2-C3 antibody failed to reveal any difference in staining patterns between the genotypes, neither in the crypt goblet cells nor in the secreted mucus (Fig 1A and 1B, green). Moreover, the stratified structures of the inner mucus were very similar (Fig 1B and 1i). These findings indicate normal Muc2 protein expression and mucus structure in *Clca1* ^-/-^ mice. Staining with an anti-Clca1 antibody revealed, in contrast to previous data [13], a low expression of Clca1 in the proximal colon and higher expression in the distal colon (Fig 1A, red). We therefore focused the following experiments on the distal colon. The merged picture of Muc2 and Clca1 in WT confirmed co-localization of Clca1 to Muc2 producing goblet cells and presence of Clca1 in the secreted mucus (Fig 1A merge).

The specificity of the anti-CLCA1 antibody specificity was verified by staining *Clca1* ^*-/-*^ distal colon sections as negative controls and comparing the staining pattern between antibodies directed against either the amino- or the carboxy terminus of Clca1 in WT sections (Fig 1C).

### Lack of Clca1 has no effect on the abundance of other proteins in the intestinal mucus

**Fig 2:**
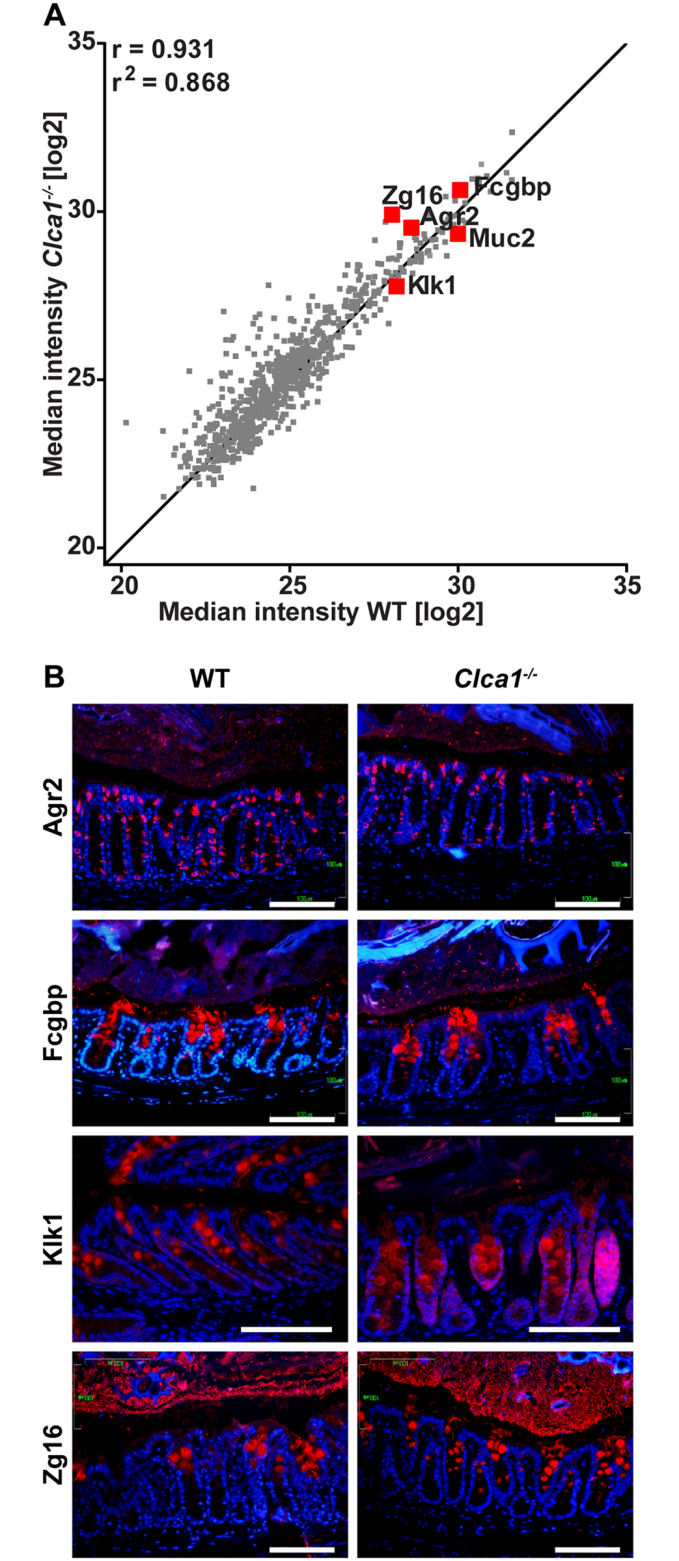
Major mucus components—Agr2, Fcgbp, Klk1, Muc2 and Zg16 –are unaltered in *Clca1* ^*-/-*^colonic mucus compared to WT controls. (A) Scatter plot depicting correlation in the median label-free intensity of all mucus proteins between WT and *Clca1* ^*-/-*^ mice by proteome analysis using mass spectrometry with the correlation coefficient (Pearson’s r) and r^2^ shown. Proteins marked with red represent known major mucus components (Agr2, Fcgbp, Klk1, Muc2 and Zg16). Line = x + 1; n = 5 per group. (B) IF of Agr2, Fcgbp, Klk1 or Zg16 (red) in WT and *Clca1* ^*-/-*^ distal colon sections, confirmed that there is no difference in protein expression in any of the major mucus proteins in *Clca1* ^*-/-*^ mice compared to WT, n = 2–3 per group. Blue = Hoechst 34580 DNA-stain, scale bars 100 μm.

To study possible further effects of Clca1-deficiency on Muc2 and other important components of the mucus, such as Agr2, Fcgbp, Klk1 and Zg16, a proteomics study was performed. The relative abundances of proteins were compared in the mucus layer, displayed in a scatter plot showing a tight and significant correlation between WT and *Clca1* ^-/-^ mucus proteins (Pearson´s r = 0.931, r^2^ = 0.868, p < 0.0001, based on 809 valid pairs; Fig 2A). No significant differences in mucus protein expression were observed between the genotypes, except for the complete absence of the Clca1 protein in *Clca1* ^-/-^ mice. The major mucus components Agr2, Fcgbp, Klk1 and Zg16 were also investigated by IF in distal colon sections with no differences found in their expression patterns or signal intensity between *Clca1* ^*-/-*^ and wild type mice (Fig 2B).

Of the eight murine Clca family members, except for Clca1 in WT mice, only Clca4a and Clca4b (formerly known as mClca6 and mClca7, respectively, see revised nomenclature S1 Table) were identified in the mucus; however, they were only detected in a few samples with very few unique peptides and without any difference between the genotypes. The mass spectrometry proteomics data are presented in S7 Table and have been deposited to the ProteomeXchange Consortium with the dataset identifier PXD001804.

### Mucus thickness, growth rate and impenetrability do not differ between WT and *Clca1* ^-/-^ mice

The mucus phenotype of WT and *Clca1* ^-/-^ mice was characterized in a set of *ex vivo* experiments examining the distal colonic mucus in which Clca1 is most abundantly detected.

**Fig 3:**
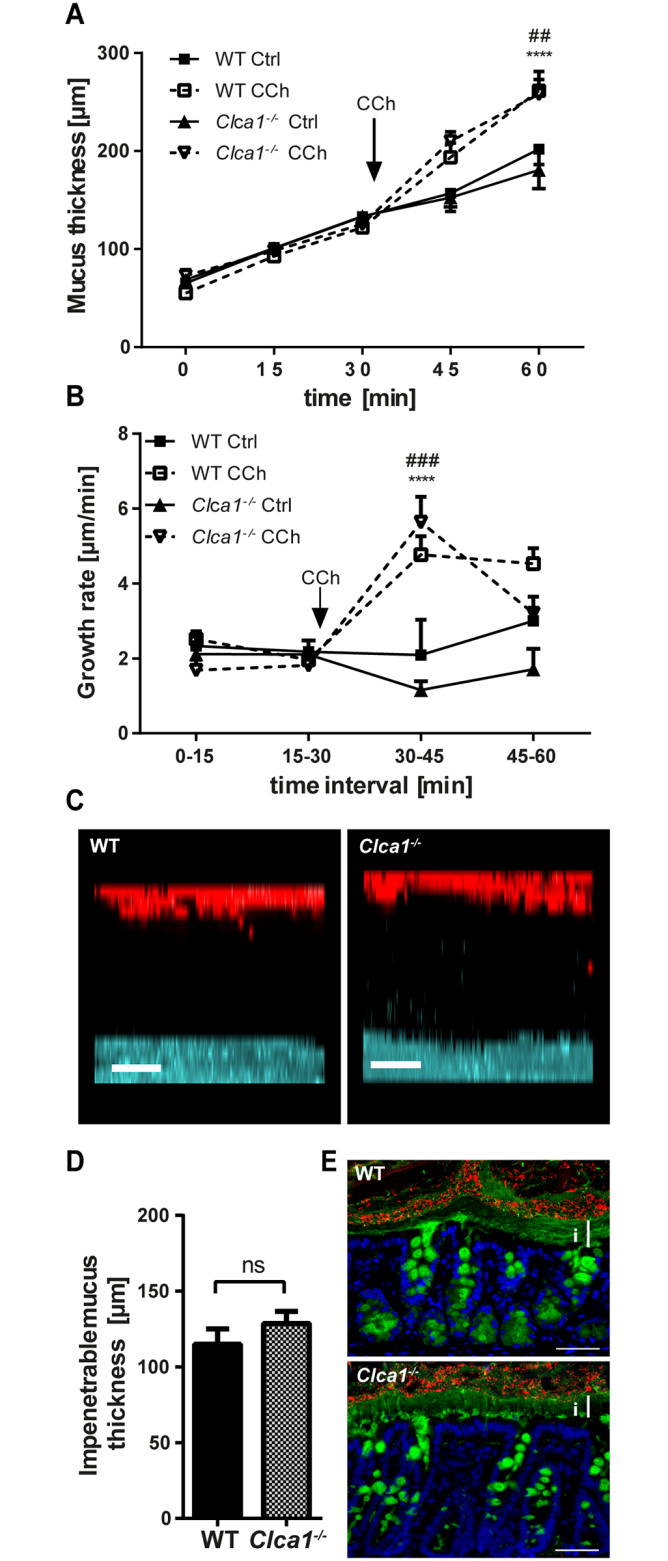
*Clca1* ^*-/-*^ has no effect on mucus growth, responsiveness and penetrability in *Clca1* ^*-/-*^ mice. (A) After flushing distal colon explants, mucus thickness and growth were similar in *Clca1* ^*-/-*^ mice (dotted lines) compared to WT (filled lines). After CCh stimulation, an increase in mucus thickness compared to unstimulated explants was observed both in WT and in *Clca1* ^*-/-*^ mice. Ctrl = control. (B) The growth rate was constant during unstimulated conditions whereas a significant growth rate increase in response to CCh was evident in both groups shortly after its addition. No significant difference was observed between the groups. Ctrl = control. (C) *Ex vivo* mucus penetrability assessment using bacteria-sized beads (1 μm, red) and confocal microscopy was performed. Representative z-stack projections from WT and *Clca1* ^*-/-*^ mucus 30 minutes after tissue mounting both showed a clear separation between the tissue (blue) and the beads (red). Scale bars 50 μm. (D) The impenetrable mucus thickness, measured as the distance between the tissue and the sedimented beads in the confocal z-stacks did not differ between WT and *Clca1* ^*-/-*^ mice. ns = non-significant. (E) FISH with a general bacterial 16S probe (EUB338, red), counterstained for Muc2 (anti-MUC2-C3, green) and DNA (Hoechst 34580, blue) in sections from distal colon confirmed the impenetrability of the inner mucus layer both in WT and *Clca1* ^*-/-*^ mice with a clear separation of the tissue and bacteria. i = inner mucus layer. Scale bars 100 μm. n = 5 per group. Data are presented as mean ± SEM. ^##^ p < 0.01, ^###^ p < 0.001 for WT Ctrl vs. WT CCh; ****p < 0.0001 for *Clca1* ^*-/-*^ Ctrl vs. *Clca1* ^*-/-*^ CCh.

Mucus was measured to investigate the initial thickness of the inner, firmly attached mucus as well as the secreted and expanding mucus over time both under normal and stimulated conditions. The outer, non-adherent mucus layer was flushed away prior to removal of the longitudinal muscle layer, providing an initial measurement representing the inner, adherent mucus thickness. Neither the thickness of the adherent mucus layer (65.3 ± 5.1 μm and 68.6 ± 5.8 μm for WT and *Clca1* ^-/-^ mice, respectively; Fig 3A) nor the spontaneous mucus growth in the explant chamber system during 1 h (total thickness of 202.0 ± 15.8 μm and 180.7 ± 19.0 μm at t = 60 min with an average growth rate of 2.40 ± 0.21 μm/min and 1.89 ± 0.21 μm/min for WT and *Clca1* ^*-/-*^ mice, respectively; Fig 3A and 3B) differed between the two genotypes. Increased mucus secretion was evident in both WT and *Clca1* ^*-/-*^ tissue after addition of CCh, a cholinergic agonist known to induce intestinal epithelial ion and mucus secretion, and a significantly thicker mucus layer compared to unstimulated tissue was measured after a total of 60 min in the chamber (261.6 ± 20.0 μm vs. 202.0 ± 15.8 μm in WT, p < 0.01 and 258.1 ± 15.0 μm vs. 180.7 ± 19.0 μm in *Clca1* ^*-/-*^ mice, p < 0.01; Fig 3A). No difference in total mucus thickness before and after CCh stimulation was noted between WT and *Clca1* ^*-/-*^ mice (261.6 ± 20.0 μm vs. 258.1 ± 15.0 μm, respectively). The mucus growth rate showed an immediate peak response to CCh at 30–45 min in tissue explants of both genotypes (Fig 3B). The difference observed could indicate a small shift in peak response time that might be from variation in administration in the basolateral perfusate. In addition, a drop in the transepithelial potential difference, indicating the presence of an ion transport, was noted concurrently with the increase in mucus growth rate both in WT and *Clca1* ^*-/-*^ mice.

To test whether Clca1 has any structural relevance for mucus barrier formation, a penetrability and distribution assay was performed in which bacteria-sized beads were applied on top of the mucus and their distribution in the mucus was investigated by confocal microscopy. Both in WT and in *Clca1* ^*-/-*^ mice, a clear separation between the epithelium and the beads sedimenting on top of the mucus was observed (Fig 3C). Impenetrable mucus thickness was the same for WT and *Clca1* ^*-/-*^ mice (115.0 ± 10.2 μm and 128.6 ± 8.1 μm in WT and *Clca1* ^*-/-*^ mice, respectively; Fig 3D). The exclusion of bacteria from the inner mucus layer was confirmed for both genotypes with FISH using a general bacterial probe and Muc2 counterstaining (Fig 3E).

### The mucus barriers of WT and *Clca1* ^*-/-*^ mice are identically affected under DSS challenge

**Fig 4:**
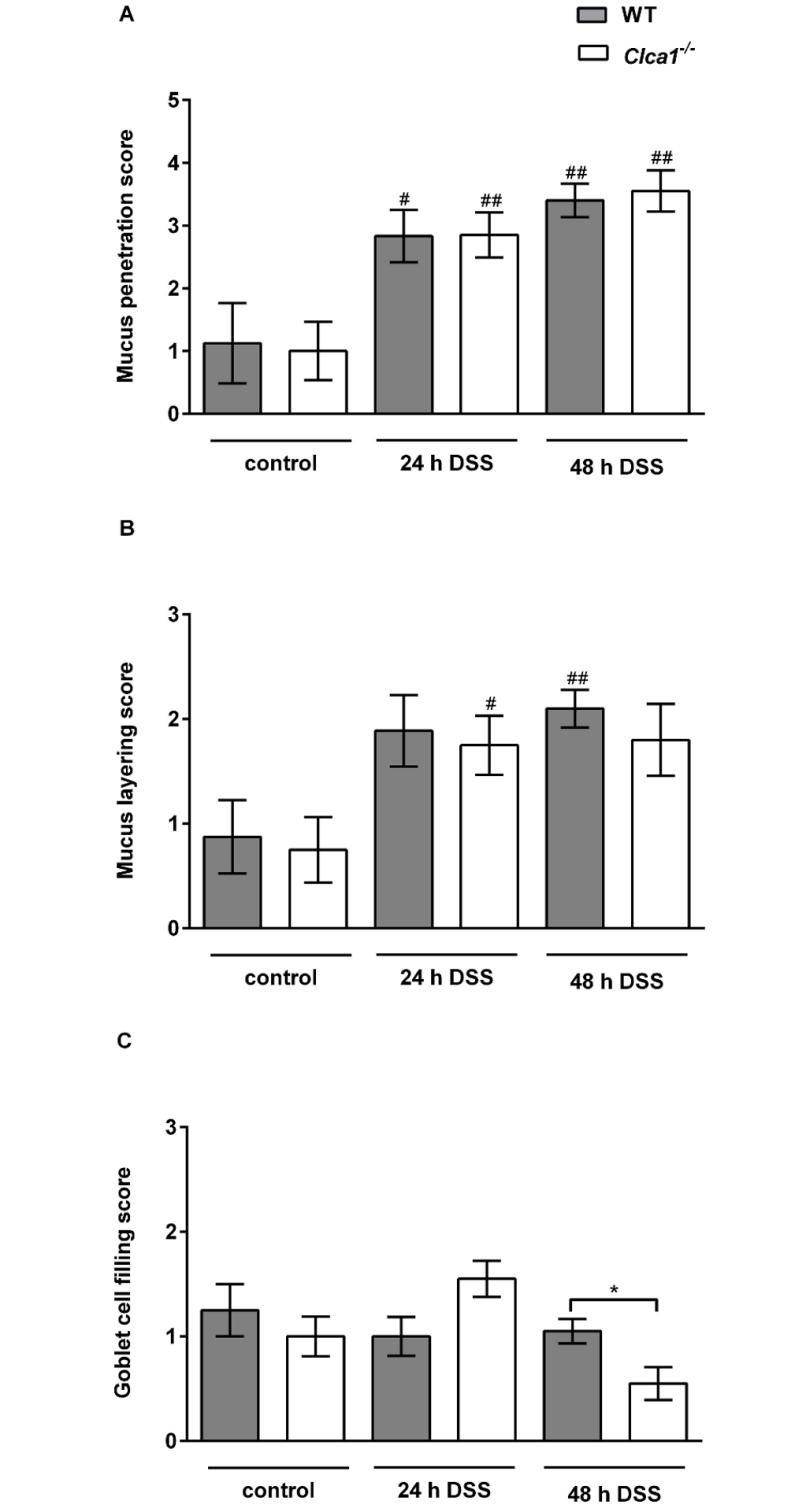
The mucus barrier of WT and *Clca1* ^*-/-*^ mice are identically affected during DSS challenge. Mucus penetration, mucus layering and goblet cell filling in the colon of mice after 24 and 48 h DSS treatment were assessed using IF microscopy. (A) Mucus penetration increased identically under DSS treatment, however, without any significant difference between the genotypes. (B) The mucus layering score also increased under DSS influence without any observable difference between the genotypes. (C) The goblet cell filling score did not show any statistically significant differences, neither between genotypes nor between naive vs. DSS-treated animals, except for WT vs. *Clca1* ^*-/-*^ mice after 48 hours. Mean values (n = 8 to 10). The scoring system is depicted in S3 Table and S4 Table. ^#^p < 0.05 and ^##^p < 0.01 versus the naive control group. *p < 0.05 as indicated. Scale bars 50 μm.

Mucus penetration and layering of the inner, stratified mucus layer and goblet cell filling were assessed via FISH and IF microscopy after 24 and 48 h of DSS application. Overall, the mucus penetrability increased significantly during DSS treatment from 1.1 ± 0.6 and 1.0 ± 0.5 (naive conditions) to 2.8 ± 0.4 and 2.9 ± 0.4 (24 h DSS) and to 3.4 ± 0.3 and 3.6 ± 0.3 (48 h DSS) for WT and *Clca1* ^*-/-*^ mice, respectively (p < 0.01 for all groups except WT naive vs. 24 h DSS p < 0.05; Fig 4A). This clearly reflects the expected loss of mucus barrier integrity within the first 48 h of DSS administration [28]; however, without any significant difference between the two genotypes.

The mucus layering score increased under the influence of DSS without any observable difference between the genotypes from 0.9 ± 0.4 and 0.8 ± 0.3 (naive conditions) to 1.9 ± 0.3 and 1.8 ± 0.3 (24 h DSS) and to 2.1 ± 0.2 and 1.8 ± 0.3 (48 h DSS) for WT and *Clca1* ^*-/-*^ mice, respectively (p < 0.05; Fig 4B), due to loss of mucus structure which correlates well with the penetration score.

The goblet cell filling score remained similar from 1.3 ± 0.3 and 1.0 ± 0.2 (naive conditions) to 1.0 ± 0.2 and 1.6 ± 0.2 (24 h DSS) and to 1.1 ± 0.1 and 0.6 ± 0.2 (48 h DSS) for WT and *Clca1* ^*-/-*^ mice, respectively. A significant increase in goblet cell filling was observed only at the single time point of 48 h for *Clca1* ^*-/-*^ compared to WT mice (p < 0.05; Fig 4C).

### Loss of Clca1 has no effect on bacterial translocation in naive and DSS-challenged mice

In addition to characterizing bacterial mucus penetration via FISH, we also determined the relative translocation frequencies of live bacteria into selected sentinel organs such as blood, mesenteric lymph nodes, liver and spleen. Overall, there was neither a difference between the genotypes nor between naive and colitic mice of the same genotype (data not shown).

### Fecal microbiota composition, blood content and stool consistency are not altered during acute colitis in WT and *Clca1* ^*-/-*^ mice

**Fig 5:**
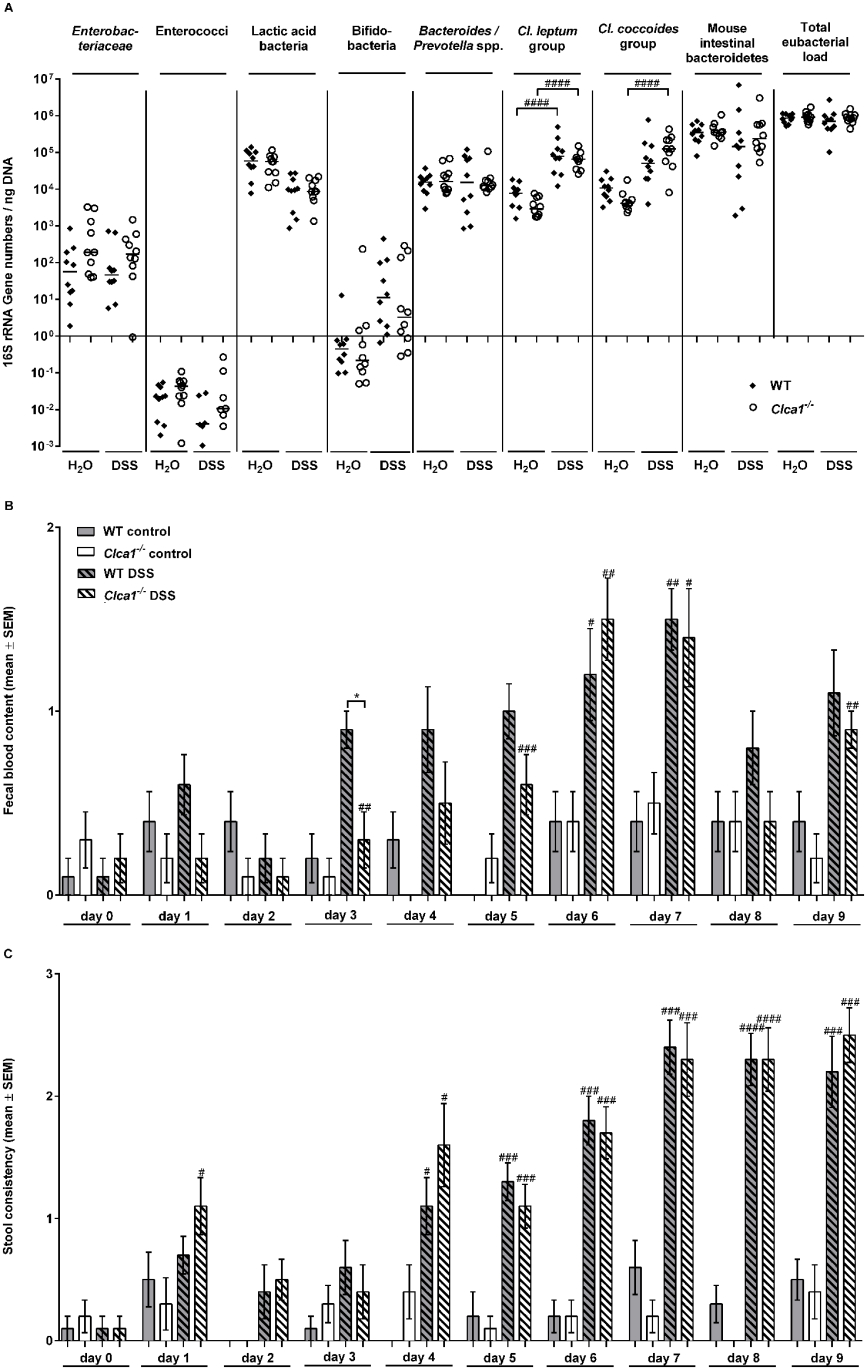
The fecal microbiota composition, blood content and stool consistency are altered during DSS-colitis without differences between *Clca1* ^*-/-*^ and wild type mice. (A) Real-time-PCR analyses, amplifying bacterial 16S rRNA variable regions and 16S rRNA gene numbers/ng DNA derived from colon samples, were employed in order to analyze colonic microbiota composition regarding main intestinal bacterial groups—*Enterobacteriaceae*, enterococci, lactic acid bacteria, bifidobacteria, *Bacteroides/Prevotella spp.*, *Clostridium leptum* group, *Clostridium coccoides* group, mouse intestinal bacteroidetes, and total eubacterial load—whilst comparing WT and *Clca1* ^*-/-*^ fecal samples collected before and after DSS treatment (7 d-group). In the naive state, mice of either genotype had a comparable fecal microbiota composition. Following colitis induction, increases in the colonic *Clostridium leptum* and *Clostridium coccoides* group as well as in bifidobacteria were evident, yet without any statistically significant difference between the genotypes. A minimum cut-off value of 10^0^ gene copy numbers/ng DNA and a minimum log-change of 1 were set as limits of statistically significant relevance. Medians (n = 9 to 10) and significance levels (p values) determined by the Mann-Whitney-U test are indicated. ^####^p < 0.0001 vs. before DSS application. (B) Fecal blood content and (C) stool consistency scores revealed an overall increase in the course of DSS administration without any statistically significant differences between genotypes, except for a higher fecal blood content score in WT vs. *Clca1* ^*-/-*^ mice at day 4. Mean values (n = 9 to 10) ± SEM and significance levels (p values) determined by the Mann-Whitney-U test are indicated. No boxplot = scores of all animals in this group were zero. ^#^p < 0.05, ^##^p < 0.1, ^###^p < 0.01 and ^####^p < 0.001 versus the naive control group. *p < 0.05 as indicated.

Since a change in intestinal microbiota composition gives rise to an altered chemical mucus composition and goblet cell function [53], we quantified selected species of the fecal microbiota in colonic fecal samples of mice before and after treatment with DSS (7 d-group) by real-time PCR, with a comparison between WT and *Clca1* ^*-/-*^ mice (Fig 5A).

In the naive state, mice of either genotype had a very similar microbiota composition. Following colitis induction, increases in the colonic *Clostridium leptum* group and in the *Clostridium coccoides* group were statistically significant. Bifidobacteria were only detectable above the cut-off value of 10^0^ gene copy numbers/ng DNA during colitis with no statistically significant difference between the genotypes.

Fecal blood content (Fig 5B) and stool consistency scores (Fig 5C) revealed an overall increase over the course of DSS administration, but without any statistically significant differences between genotypes, except for a higher fecal blood content score of WT vs. *Clca1* ^*-/-*^ mice at day 4.

### Clca1 deficiency has no effect on the mRNA expression of mucin genes under naive and DSS challenged conditions

**Fig 6:**
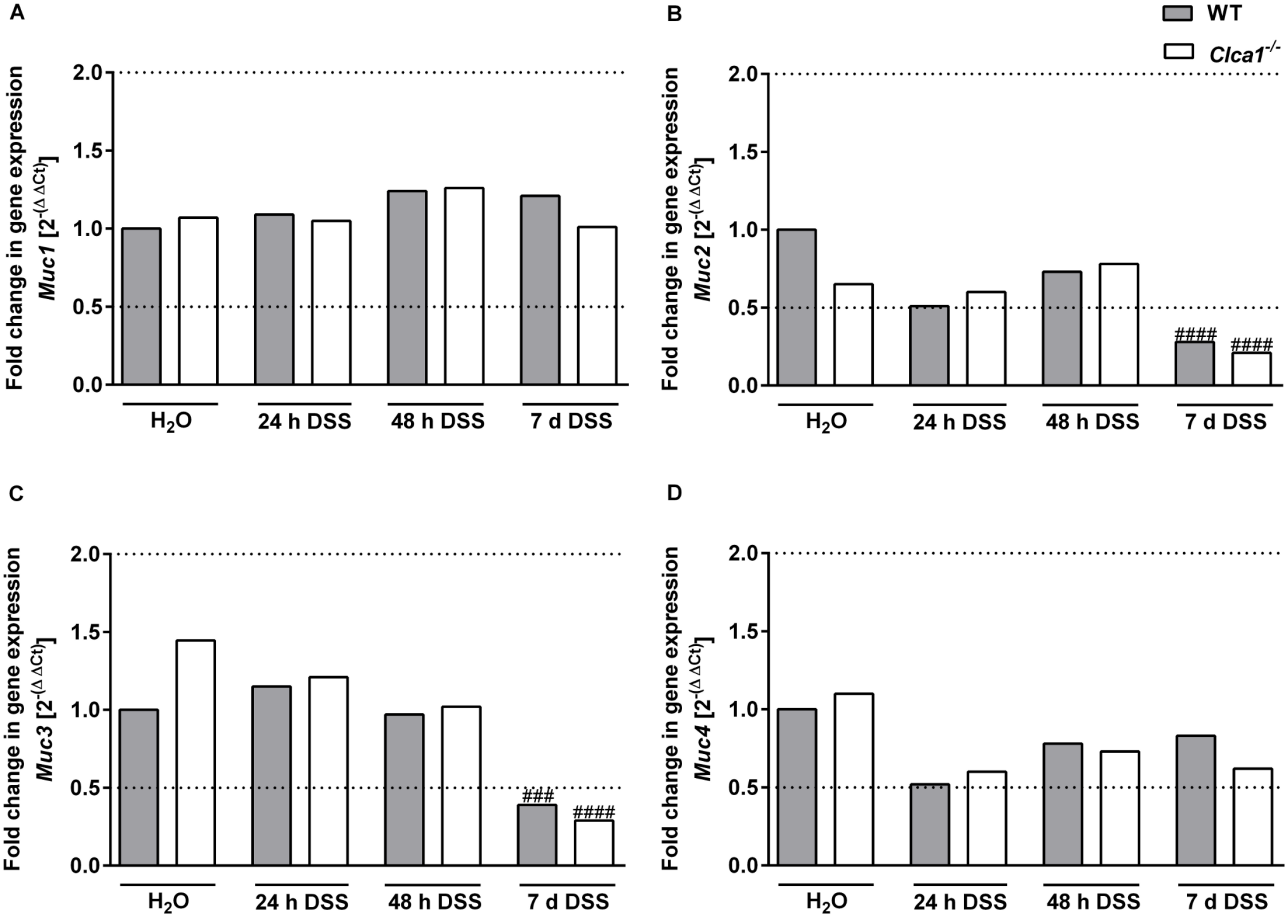
Clca1-deficiency has no effect on the mRNA expression of mucin genes in the intestinal tract under naive and DSS challenged conditions. After treatment of mice with DSS for 24 or 48 h as well as for 7 d with respective controls (n = 9–16 per group), mRNA expression levels of mucin genes were determined by quantitative RT-PCR. (A) *Muc1* gene expression did not show any statistically significant difference, neither between the genotypes nor during challenge. For (B) *Muc2* and (C) *Muc3*, a statistically significant down-regulation in WT and *Clca1* ^*-/-*^ was seen in the 7 d-group compared to water controls. (D) *Muc4* also did not show any significant differential expression, neither between nor amongst the genotypes. Dotted lines indicate a fold change of 0.5 and 2, respectively, as limits for valid statement of lowered and elevated parameters. Ct, cycle threshold. ^###^p < 0.001 and ^####^p < 0.0001 vs. the naive control group.

After treatment with DSS for 24 or 48 h as well as for 7 d (n = 9–16 for each group), mRNA expression levels of selected mucin genes (*Muc1*, *2*, *3*, *4*, *5ac*, *5b* and *6*) were compared by RT-qPCR in distal colon samples to those of respective controls (Fig 6A–6D). *Muc1* gene expression (Fig 6A) did not show any statistically significant difference between the genotypes or between naive and DSS-challenged conditions.

Only in the 7 d-group was a statistically significant difference seen compared to water controls with *Muc2* and *Muc3* (Fig 6B and 6C, respectively) both down-regulated in WT (p < 0.05 and < 0.01, respectively) as well as in *Clca1* ^*-/-*^ mice (p < 0.05 and < 0.0001, respectively). Furthermore, a slightly lower expression of *Muc2* and slightly higher expression of *Muc3* was noted in *Clca1* ^*-/-*^ mice compared to WT mice under naive conditions which levelled out in the course of DSS challenge.

No significant differential expression was observed for *Muc4* (Fig 6D). Overall, *Muc5ac*, -*5b* and *-6* failed to show any relevant expression in the distal colon of WT and *Clca1* ^*-/-*^ mice under naive and DSS challenged conditions.

## Discussion

CLCA1 is expressed and secreted by goblet cells in all investigated species so far including humans [9], mice [10], horses [54], and pigs [55] and is a major constituent of the intestinal mucus [10,11,13]. In CF knockout mice Clca1 expression is reduced [26] while experimental overexpression in CF ameliorates the murine intestinal mucus phenotype, characterized by mucus inspissation and intestinal obstruction [23]. A similar correlation has been found in humans where a certain allelic variant of CLCA1 is significantly overrepresented in CF patients with aggravated intestinal disease [56]. These observations have given rise to the hypothesis that Clca1 play a role in intestinal goblet cell function, mucus properties or modulation of secretion [23]. Consequently, we also speculated that it may have an impact on the protective function of the intestinal mucus barrier.

This study therefore focused on the characterization of the intestinal mucus phenotype in WT controls versus *Clca1* ^*-/-*^ mice. The expression of Clca1, which colocalizes with Muc2 increases along the intestinal axis and thus parallels the increasing impenetrability of the mucus [57]. This also supported our hypothesis that CLCA1 may have a function in the intestinal mucus barrier.

Much to our surprise, the data presented here failed to reveal any involvement in normal mucus structure and function in the model and under the conditions used. No difference in mucus thickness, adherence to the epithelium or transformation from inner to outer mucus was observed between the genotypes, indicating normal mucus secretion and processing in the absence of Clca1. Additionally, an equal responsiveness to the secretagogue carbachol and quality of the mucus preventing bacterial penetration towards the epithelium was observed in *Clca1* ^*-/-*^ and WT mice. The relative protein abundance and expression pattern of the main structural mucus component Muc2 and of other mucus proteins, such as Agr2, Klk1, Fcgbp, Zg16 were unaltered in the Clca1-deficient tissues. The proteomics data also failed to reveal significant changes in mucus composition of *Clca1* ^*-/-*^ mice except for the lack of Clca1.

Our data analysis panel is well in line with and complements previous, solely histological investigations which failed to reveal any intestinal phenotype of various unchallenged *Clca1* ^*-/-*^ models [52,58]. In addition to unchallenged conditions, we also tested for effects of lack of Clca1 in comparison to WT mice after DSS-challenge. The highly sulphated dextran of DSS has a dramatic and rapid destructive impact on the mucus structure and is commonly used as an inducer of colitis in mice [28,59]. Overall, the statistically significant bacterial penetrability increase between the respective naive and DSS-treated groups of the same genotype confirmed the expected loss of mucus barrier integrity within the first 48 h after initial DSS administration [28], but without any significant difference between the genotypes. The overall loss of layering structure within the first 48 h is also in line with previous observations [28]. The only statistically significant difference observed was the higher filling status of *Clca1* ^*-/-*^ goblet cells after 48 h of DSS administration. This could either reflect a decreased mucus release in *Clca1* ^*-/-*^ mice which is contradicted by the mucus structure analysis or may result from an increased synthesis which is contradicted by the gene expression data, both of which show no difference between the genotypes. We therefore feel that the statistical significance of the goblet cell filling score may be biologically debatable. However, we cannot exclude that Clca1 plays a role in exocytosis of mucin granules.

Furthermore, we quantified the induction of selected mucin genes in the distal colon. Mucins are subdivided into the goblet cell secreted gel-forming mucins such as Muc2, Muc5ac, Muc5b and Muc6 and the transmembrane mucins, expressed by epithelial cells, such as Muc1, Muc3 and Muc4 [60–62]. Of these Muc1 to 4 are expressed in the large intestine [63]. No significant alterations in intestinal mucin gene expression were observed between the genotypes in unchallenged conditions although a slightly lower expression of the secreted mucin *Muc2* and slightly higher expression of the membrane-bound *Muc3* were noted in *Clca1* ^*-/-*^ mice compared to WT mice. This difference disappeared during the course of DSS application. In the 7 d-group, *Muc2* and -*3* were, in a very similar fashion, significantly down-regulated in WT and *Clca1* ^*-/-*^ mice compared to water controls which is in line with decreased Muc2 mucin synthesis observed under pathological conditions [64]. It should also be mentioned that RNA levels often poorly correlate with protein levels in the mucus since goblet cells are capable of storing large amounts of mucus. We also failed to detect any changes in Muc2 expression along the intestinal tract by IF or in the relative abundance of Muc2 in the secreted mucus as observed by our proteomics data.

Muc5ac and 5b are the main respiratory mucins. Muc5ac is also expressed in the stomach and is transported through the intesinal tract [13] while MUC5B is expressed at low levels in human colon [65,66] but is not normally observed in the murine gastrointestinal tract [67,68]. MUC6 expression is normally restricted to the stomach and duodenum [69]. Muc5ac expression was of particular interest due to its observed induction by CLCA1 overexpression in airway cell culture systems and in the respiratory tract [5,33]. In Crohn´s disease, MUC5AC expression is known to be induced in the intestine together with MUC5B and MUC6 [70]. Muc5ac is also expressed in the intestine upon clearance of helminth infections [71]. Besides Muc5ac, Muc6 was also of interest as it exhibited a transient *de novo*-expression in *Muc2*-deficient mice, possibly due to a compensatory protective mechanism within the colonic epithelium [72] although this has never been verified at protein level. In our study, *Muc5ac* and -*5b* as well as *Muc6* failed to show any significant expression in the distal colon, neither in the *Clca1* ^*-/-*^ model nor during pathological conditions regardless of the genotype used. The lack of *Muc5ac*, *-5b* and -*6* on RNA-level is in line with previous reports that failed to detect these mucins at the protein level in unchallenged WT mice [13]. With this data we failed to observe any impact of *Clca1*-deficiency on mucin gene expression in the distal colon. Similar results had been obtained for the murine respiratory tract under naive and challenged conditions [47,52,58]. In contrast, it had been shown that human CLCA1 activates an Il-13-dependent MAPK-mediated airway mucin expression. These differences in mucin gene expression between mice and humans are perhaps due to compensatory effects of certain murine CLCA members [8]. It has previously been argued that, additionally to Clca1, the murine Clca2, -4a and -4b may also induce airway gene expression [8]. Of note, Clca4a and -4b have previously been found to be upregulated at the mRNA level in *Clca1* ^*-/-*^ mice [52]. Here, however, only minute traces of Clca4a and -4b were identified in the secreted mucus without any difference between the genotypes. Clca4a is known to be expressed by enterocytes and its amino-terminal part is released into the mucus [21]. Clca4b is also expressed in the intestine [52,73]. However, its expressing cell type is unknown to date. The Clca2 protein which is expressed in keratinocytes of stratified epithelia and in distinct niches of the respiratory tract [48,74] has not been detected in the intestinal tract to date which is consistent with our findings [48]. The absence of any differential Clca expression pattern in the mucus on the protein level suggests that the lack of a phenotype may not be due to a phenotype rescue by other Clca homologs.

Intestinal microbiota composition was investigated with a focus on main gut bacterial groups which are known to be altered during colitis development [30,44]. After induction of acute DSS colitis, an increase in the colonic clostridia and bifidobacteria was observed which is consistent with previous findings, yet without significant differences between the genotypes [30]. The expected increased scores of fecal blood content and stool consistency, monitored as clinical parameters of colitis occurred independently of the genotype [34,35].

Taken together our data point towards a function of CLCA1 other than in mucus structure, function and barrier integrity. As an alternative function a role for CLCA1 as a signaling molecule regulating cytokine expression has become the focus of recent research. In the respiratory tract, *Clca1* ^*-/-*^ mice develop decreased levels of Cxcl-1, a potent neutrophil chemoattractant [75,76], and of Il-17, a proinflammatory cytokine [77] at the mRNA and protein levels with a consequent decrease in neutrophil response during *Staphylococcus aureus* pneumonia [47]. This observation is in line with reduced Clca1 and CXCL-1 in an IL-17 neutralization study [78]. Consistent with this notion is that the human CLCA1 presumably induces a proinflammatory macrophage response [79]. It has therefore been speculated that CLCA1 may play a role in the modulation of an innate immunity response or cytokine regulation. As a second possible function it has been suggested that CLCA1 may play a role in the differentiation and proliferation of intestinal epithelial cells and colorectal cancer cells [80,81]. Finally, it was recently discovered that secreted CLCA1 increases the surface expression of TMEM16A (Anoctamin1/DOG1), the first genuine calcium-activated chloride channel, in a paracrine fashion and thereby increases the calcium-dependent chloride current *in vitro* [82]. Future experiments will have to clarify whether and how CLCA1 actually fulfills and possibly even combines several of these suggested functions and how they may relate to the proposed roles of CLCA1 in maladies with secretory dysfunctions. A role for CLCA1 as a signaling molecule affecting the integrity of the epithelial barrier could also be a new area of interest. In conclusion, our results show that Clca1 is not required for intestinal mucus synthesis, structure and barrier function in naive or DSS-challenged mice.

## Supporting Information

S1 Table Revised CLCA nomenclature. (PDF)

S2 Table Scoring system for bacterial penetration of the inner mucus layer. (PDF)

S3 Table Scoring examples for the inner mucus layer. (PDF)

S4 Table Scoring system for the goblet cell filling. (PDF)

S5 Table 16S rRNA gene group-specific primers for quantitative Real Time-PCR. (PDF)

S6 Table Primers and probes for quantitative Real Time-PCR. (PDF)

S7 Table Mucus proteome in wild type and *Clca1* ^*-/-*^ distal colon. (PDF)
