# Supplementary material for: The Goblet Cell Protein Clca1 (Alias mClca3 or Gob-5) Is Not Required for Intestinal Mucus Synthesis, Structure and Barrier Function in Naive or DSS-Challenged Mice
Source: PLoS One. 2015 Jul 10;10(7):e0131991. doi: 10.1371/journal.pone.0131991 (PMC4498832; doi:10.1371/journal.pone.0131991)
Supplement: S1 Table — (PDF) [file pone.0131991.s001.pdf]

S1 Table. Revised CLCA nomenclature

Human symbol	New mouse symbol[1]	Previously used mouse symbols	Gene bank accession number
**CLCA1**	**Clca1**	**mClca3, gob-5**	**NM 017474**
**CLCA2**	**Clca2**	**mClca5**	**NM 178697**
**CLCA3P**	**Clca3a1**	**mClca1**	**NM 009899**
	**Clca3a2**	**mClca2**	**NM 030601**
	**Clca3b**	**mClca4**	**NM 139148**
**CLCA4**	**Clca4a**	**mClca6**	**NM 207208**
	**Clca4b**	**mClca7 (AI747448)**	**NM 0001033199**
	**Clca4c**	**Clca8 (Gm6289, EG622193, A730041H10Rik)**	**NM 001039222**

[1]Renamed by the Mouse Gene Nomenclature Committee (MGNC) due to a realignment of the *Clca* genes with respect to human and rat nomenclature in accordance with HUGO (Human Gene Nomenclature Committee) und RGD (Rat Genome Database).
