# Supplementary material for: The Goblet Cell Protein Clca1 (Alias mClca3 or Gob-5) Is Not Required for Intestinal Mucus Synthesis, Structure and Barrier Function in Naive or DSS-Challenged Mice
Source: PLoS One. 2015 Jul 10;10(7):e0131991. doi: 10.1371/journal.pone.0131991 (PMC4498832; doi:10.1371/journal.pone.0131991)
Supplement: S2 Table — (PDF) [file pone.0131991.s002.pdf]

S2 Table. Scoring system for bacterial penetration of the inner mucus layer

Bacterial penetration score[1]	
**0**	**The inner mucus layer separates bacteria from epithelium**
**1**	**Few bacteria in the inner mucus layer**
**2**	**More bacteria entering the mucus but are not in contact with the epithelium**
**3**	**Some few bacteria are in contact with the epithelium**
**4**	**A lot of bacteria are in contact with epithelium**
**5**	**All epithelial surfaces have contact with bacteria**

[1] According to Johansson 2010 and 2014 [1;2]
