# Supplementary material for: The Goblet Cell Protein Clca1 (Alias mClca3 or Gob-5) Is Not Required for Intestinal Mucus Synthesis, Structure and Barrier Function in Naive or DSS-Challenged Mice
Source: PLoS One. 2015 Jul 10;10(7):e0131991. doi: 10.1371/journal.pone.0131991 (PMC4498832; doi:10.1371/journal.pone.0131991)
Supplement: S3 Table — (PDF) [file pone.0131991.s003.pdf]

S3 Table. Scoring examples for the inner mucus layer

Mucus layering score			
**Score 0**	**Score 1**	**Score 2**	**Score 3**
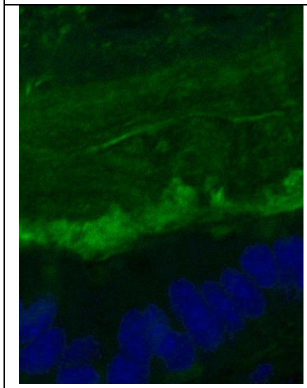	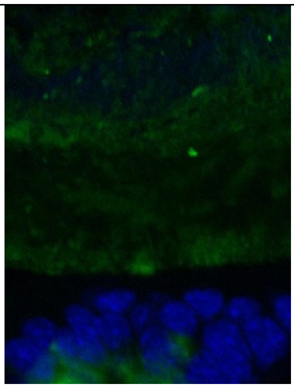	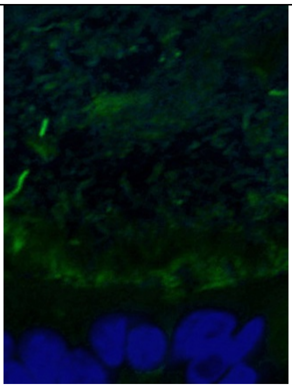	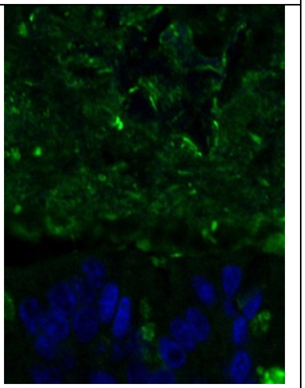
Clearly visible layering with stratified structure of the inner mucus layer	Observable layering in the inner mucus layer but with less overall structure	Remains of layering in the inner mucus are visible	No structure at all in the mucus can be observed

Green: Muc2; blue: nuclei
