# Supplementary material for: The Goblet Cell Protein Clca1 (Alias mClca3 or Gob-5) Is Not Required for Intestinal Mucus Synthesis, Structure and Barrier Function in Naive or DSS-Challenged Mice
Source: PLoS One. 2015 Jul 10;10(7):e0131991. doi: 10.1371/journal.pone.0131991 (PMC4498832; doi:10.1371/journal.pone.0131991)
Supplement: S4 Table — (PDF) [file pone.0131991.s004.pdf]

S4 Table. Scoring system for the goblet cell filling

Goblet cell filling score[1]			
**Score 0**	**Score 1**	**Score 2**	**Score 3**
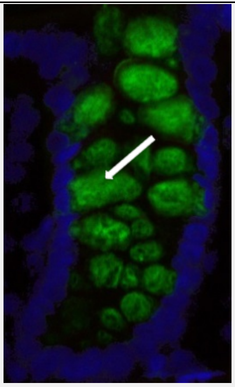	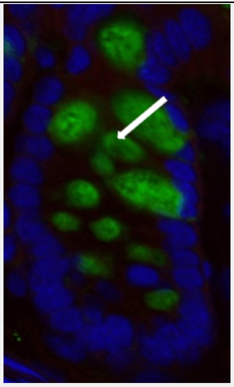	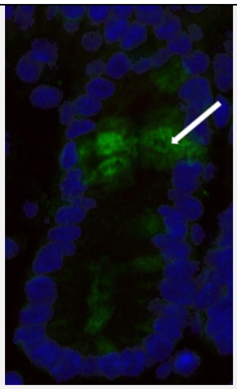	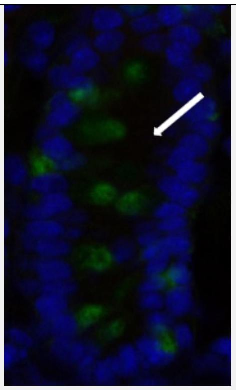
Highly filled and densely packed goblet cells	Moderately filled and loosely packed goblet cells	Minimally filled goblet cells	Depleted goblet cells

[1] Modified from Johansson 2014 [2]

Green: Muc2; blue: nuclei; arrows: representative goblet cell filling status
