# Supplementary material for: The Goblet Cell Protein Clca1 (Alias mClca3 or Gob-5) Is Not Required for Intestinal Mucus Synthesis, Structure and Barrier Function in Naive or DSS-Challenged Mice
Source: PLoS One. 2015 Jul 10;10(7):e0131991. doi: 10.1371/journal.pone.0131991 (PMC4498832; doi:10.1371/journal.pone.0131991)
Supplement: S5 Table — (PDF) [file pone.0131991.s005.pdf]

S5 Table. 16S rRNA gene group-specific primers for quantitative Real Time-PCR[1]

Main bacterial groups (target)	Bacterial reference strain	Forward primer (5´ - 3´)	Reverse primer (5´ - 3´)	References
Enterobacteriaceae (γ-Proteobacteria/ *Enterobacteriaceae*)	*Escherichia coli* ATCC 25922	AAACTCAAATGAATTGACGG	CTTTTGCAACCCACTCC	Kühbacher 2006 [3]
Enterococci	*Enterococcus gallinarium*	ATCAGAGGGGGATAACACTT	ACTCTCATCCTTGTTCTTCTC	Matsuda 2009 [4]
Lactic acid bacteria (*Lactobacillus* group[2])	*Lactobacillus acidophilus* DSM 20079	AGCAGTAGGGAATCTTCCA	CACCGCTACACATGGAG	Heilig 2002 [5] Walter 2001 [6]
Bifidobacteria (*Bifidobacterium* genus)	*Bifidobacterium* sp. (murine origin)	CTCCTGGAAACGGGTGG	GGTGTTCTTCCCGATATCTACA	Matsuki 2002 [7]
Bacteroides/ Prevotella spp. (*Bacteroides* group[3])	*Bacteroides ovatus* DSMZ 1896	GAAGGTCCCCCACATTG	CAATCGGAGTTCTTCGTG	Bartosch 2004 [8]
Clostridium leptum group (*Clostridium leptum* subgroup[4])	*Clostridium leptum* DSMZ 753	TTACTGGGTGTAAAGGG	TAGAGTGCTCTTGCGTA	Van Dyke 2002 [9]
Clostridium coccoides group (*Clostridium coccoides*[5])	*Clostridium coccoides* DSMZ 935	AAATGACGGTACCTGACTAA	CTTTGAGTTTCATTCTTGCGAA	Matsuki 2002 [7]
Mouse intestinal bacteroidetes (Mouse Intestinal Bacteroides)	MIB plasmid 16-1	CCAGCAGCCGCGGTAATA	CGCATTCCGCATACTTCTC	Barman 2008 [10]
Total eubacterial load (Domain Bacteria, targets 16S V3 region)	*Escherichia coli* ATCC 25922	CGGYCCAGACTCCTACGGG	TTACCGCGGCTGCTGGCAC	Lee 1996 [11]

[1] from Rausch 2013 [12]
[2] including *Leuconostoc*, *Pediococcus*, *Aerococcus* and *Weissella* but not *Enterococcus* or *Streptococcus*
[3] including *Prevotella* and *Porphyromonas*
[4] including *Faecalibacterium* (Fusobacterium) *prausnutzii Clostridium* 16S rRNA cluster IV
[5] *Eubacterium rectale* subgroup (*Clostridium* 16S rRNA cluster XIVa/b)
