# Supplementary material for: The Goblet Cell Protein Clca1 (Alias mClca3 or Gob-5) Is Not Required for Intestinal Mucus Synthesis, Structure and Barrier Function in Naive or DSS-Challenged Mice
Source: PLoS One. 2015 Jul 10;10(7):e0131991. doi: 10.1371/journal.pone.0131991 (PMC4498832; doi:10.1371/journal.pone.0131991)
Supplement: S6 Table — (PDF) [file pone.0131991.s006.pdf]

S6 Table. Primers and probes for quantitative Real Time-PCR

Gene	Forward primer (5´ - 3´)	Reverse primer (5´- 3´)	Probe	References
Gapdh	TCACCACCATGGAGAAGGC	GCTAAGCAGTTGGTGGTGCA	ATGCCCCCATGTTTGTGATGGGTGT	Giulietti 2001 [13]
Ef-1α	CAAAAACGACCCACCAATGG	GGCCTGGATGGTTCAGGATA	AGCAGCTGGCTTCACGCTCAGGTG	Braun 2010 [14]
B2m	ATTCACCCCCACTGAGACTGA	CTCGATCCCAGTAGACGGTC	TGCAGAGTTAAGCATGACAGTATGGCCG	Norris 2000 [15]
Muc1	TATGGGCAGCTGGACATCTT	ATTACCTGCCGAAACCTCCT	ATGAGTGAATACCCTACCTACCACA	*
Muc2	CTCCATTGAGTTTGGGAACAT	TTCGGCTCGGTGTTCAGAG	CTGAGGCAGTACAAGAACCG	*
Muc3	GGAACTGGTGGAGAGCGTAG	CGGTCCTGTAGCTTCTCACTG	TCCGTGGAAGTGAGTGTGAC	*
Muc4	GTCCACTTCTTCCCCATCTC	GTAGCCTTTGTAGCCATCACAT	CCAGGACCAGATGGCTCTGAACCT	Tetaert 2007 [16]
Muc5ac	GCGTGGAGAATGAAAAGTATGCT	CCCAGTGCCACGCCACCGTG	CATACATGCAGTTCGAGAAGAAG	*
Muc5b	GCACGTAAATGCGACTGTCT	TATCCAAGTACTCCATGGAGGCCC	ATGGACCTTGCTCTCCTGAC	Mundhenk 2012 [17]
Muc6	CACCAACACAGAAGGCTGTT	CTGTGTACCCTGTGCACCAC	CTGTGTACCCTGTGCACCAC	*

*Primers and probes designed by authors.

Reference List

1 Johansson ME, Gustafsson JK, Sjoberg KE, Petersson J, Holm L, Sjovall H, et al. Bacteria Penetrate the Inner Mucus Layer before Inflammation in the Dextran Sulfate Colitis Model. PLoS ONE 2010;5(8):e12238.

2 Johansson ME, Gustafsson JK, Holmen-Larsson J, Jabbar KS, Xia L, Xu H, et al. Bacteria penetrate the normally impenetrable inner colon mucus layer in both murine colitis models and patients with ulcerative colitis. Gut 2014 Feb;63(2):281-91.

3 Kuhbacher T, Ott SJ, Helwig U, Mimura T, Rizzello F, Kleessen B, et al. Bacterial and fungal microbiota in relation to probiotic therapy (VSL#3) in pouchitis. Gut 2006 Jun;55(6):833-41.

4 Matsuda K, Tsuji H, Asahara T, Matsumoto K, Takada T, Nomoto K. Establishment of an analytical system for the human fecal microbiota, based on reverse transcription-quantitative PCR targeting of multicopy rRNA molecules. Appl Environ Microbiol 2009 Apr;75(7):1961-9.

5 Heilig HG, Zoetendal EG, Vaughan EE, Marteau P, Akkermans AD, de Vos WM. Molecular diversity of Lactobacillus spp. and other lactic acid bacteria in the human intestine as determined by specific amplification of 16S ribosomal DNA. Appl Environ Microbiol 2002 Jan;68(1):114-23.

6 Walter J, Hertel C, Tannock GW, Lis CM, Munro K, Hammes WP. Detection of Lactobacillus, Pediococcus, Leuconostoc, and Weissella species in human feces by using group-specific PCR primers and denaturing gradient gel electrophoresis. Appl Environ Microbiol 2001 Jun;67(6):2578-85.

7 Matsuki T, Watanabe K, Fujimoto J, Miyamoto Y, Takada T, Matsumoto K, et al. Development of 16S rRNA-gene-targeted group-specific primers for the detection and identification of predominant bacteria in human feces. Appl Environ Microbiol 2002 Nov;68(11):5445-51.

8 Bartosch S, Fite A, Macfarlane GT, McMurdo ME. Characterization of bacterial communities in feces from healthy elderly volunteers and hospitalized elderly patients by using real-time PCR and effects of antibiotic treatment on the fecal microbiota. Appl Environ Microbiol 2004 Jun;70(6):3575-81.

9 Van Dyke MI, McCarthy AJ. Molecular biological detection and characterization of Clostridium populations in municipal landfill sites. Appl Environ Microbiol 2002 Apr;68(4):2049-53.

10 Barman M, Unold D, Shifley K, Amir E, Hung K, Bos N, et al. Enteric salmonellosis disrupts the microbial ecology of the murine gastrointestinal tract. Infect Immun 2008 Mar;76(3):907-15.

11 Lee DH, Zo YG, Kim SJ. Nonradioactive method to study genetic profiles of natural bacterial communities by PCR-single-strand-conformation polymorphism. Appl Environ Microbiol 1996 Sep;62(9):3112-20.

12 Rausch S, Held J, Fischer A, Heimesaat MM, Kuhl AA, Bereswill S, et al. Small intestinal nematode infection of mice is associated with increased enterobacterial loads alongside the intestinal tract. PLoS ONE 2013;8(9):e74026.

13 Giulietti A, Overbergh L, Valckx D, Decallonne B, Bouillon R, Mathieu C. An overview of real-time quantitative PCR: applications to quantify cytokine gene expression. Methods 2001 Dec;25(4):386-401.

14 Braun J, Mundhenk L, Range F, Gruber AD. Quantitative expression analyses of candidates for alternative anion conductance in cystic fibrosis mouse models. J Cyst Fibros 2010 Sep;9(5):351-64.

15 Norris MD, Burkhart CA, Marshall GM, Weiss WA, Haber M. Expression of N-myc and MRP genes and their relationship to N-myc gene dosage and tumor formation in a murine neuroblastoma model. Med Pediatr Oncol 2000 Dec;35(6):585-9.

16 Tetaert D, Pierre M, Demeyer D, Husson MO, Beghin L, Galabert C, et al. Dietary n-3 fatty acids have suppressive effects on mucin upregulation in mice infected with Pseudomonas aeruginosa. Respir Res 2007;8:39.

17 Mundhenk L, Johannesson B, Anagnostopoulou P, Braun J, Bothe MK, Schultz C, et al. mCLCA3 does not contribute to calcium-activated chloride conductance in murine airways. Am J Respir Cell Mol Biol 2012 Jul;47(1):87-93.
