# Supplementary material for: The Goblet Cell Protein Clca1 (Alias mClca3 or Gob-5) Is Not Required for Intestinal Mucus Synthesis, Structure and Barrier Function in Naive or DSS-Challenged Mice
Source: PLoS One. 2015 Jul 10;10(7):e0131991. doi: 10.1371/journal.pone.0131991 (PMC4498832; doi:10.1371/journal.pone.0131991)
Supplement: S7 Table — (PDF) [file pone.0131991.s007.pdf]

S7 Table : Mucus proteome in wild type and *Clca1* $^{-/-}$ distal colon

Protein IDs (UniProt)	Protein names	Gene names	P	UP	Unique peptides/sample WT_1	WT_2	WT_3	WT_4	WT_5	KO_1	KO_2	KO_3	KO_4	KO_5	SC [%]	MW [kDa]
G3X8U3	MCG6895	*2210016F16 Rik*	8	8	1	0	2	1	2	2	3	3	2	1	15.7	39
Q61838;D3YW52;D3YUI3;Q6GQT1	Alpha-2-macroglobulin;Alpha-2-macroglobulin 165 kDa subunit;Alpha-2-macroglobulin 35 kDa subunit	*A2m;Pzp*	32	32	1	3	4	8	4	4	11	9	4	10	24.2	166
Q8BGQ7	Alanine--tRNA ligase, cytoplasmic	*Aars*	28	27	0	0	2	4	7	4	5	10	9	4	33.7	107
P61922;P61922-2;Q3TUE8;F7C9G3;F6RN86	4-aminobutyrate aminotransferase, mitochondrial	*Abat*	12	12	0	0	2	0	2	9	6	2	1	2	33.4	56
P21447;P06795;P21440;F6SGP4;Q9DC29;B5X0E4	Multidrug resistance protein 1A;Multidrug resistance protein 1B	*Abcb1a;Abcb1b*	33	32	4	4	6	6	2	14	12	12	7	11	24.3	141
E9QN99;Q8VCR7	Alpha/beta hydrolase domain-containing protein 14B	*Abhd14b*	8	8	4	4	2	1	3	5	6	6	6	6	45.7	22
Q8CBW3-5;B7ZCU2;B7ZCU5;Q8CBW3-2;B7ZCU4;B7ZCU3;Q8CBW3-4;Q8CBW3-3;B7ZCU0;Q8CBW3;J3QNK8;P62484	Abl interactor 1;Abl interactor 2	*Abi1;Abi2*	14	14	0	1	2	0	1	3	6	3	5	3	30.7	43
Q921H8;H3BKL5;H3BJZ9;Q8VCH0;H3BKA1;H3BJC1	3-ketoacyl-CoA thiolase A, peroxisomal;3-ketoacyl-CoA thiolase B, peroxisomal	*Acaa1a;Acaa1b*	11	11	0	1	0	0	0	4	4	4	2	3	37.7	44
Q8BWT1	3-ketoacyl-CoA thiolase, mitochondrial	*Acaa2*	18	18	4	3	4	3	7	8	5	6	5	9	52.9	42
P51174	Long-chain specific acyl-CoA dehydrogenase, mitochondrial	*Acadl*	21	21	4	3	4	1	6	11	11	9	8	9	46.3	48
P45952;D6RFD7	Medium-chain specific acyl-CoA dehydrogenase, mitochondrial	*Acadm*	22	22	3	4	5	3	6	12	12	10	9	9	39.7	46
Q07417;F6RAZ3	Short-chain specific acyl-CoA dehydrogenase, mitochondrial	*Acads*	16	16	5	1	5	5	7	12	7	7	7	8	39.6	45
P50544;B1AR28	Very long-chain specific acyl-CoA dehydrogenase, mitochondrial	*Acadvl*	19	19	2	2	2	0	2	11	6	4	5	5	32.5	71
Q8QZT1	Acetyl-CoA acetyltransferase, mitochondrial	*Acat1*	20	20	4	3	8	7	10	9	7	8	6	6	45.8	45
Q8CAY6;Q80X81;G3XA25;F2Z459	Acetyl-CoA acetyltransferase, cytosolic	*Acat2;Acat3*	16	16	2	1	3	2	6	1	4	3	6	5	37	41
Q3V117;Q91V92;Q3TS02	ATP-citrate synthase	*Acly*	61	61	7	10	22	16	21	29	32	28	27	28	52	121
P28271;Q811J3	Cytoplasmic aconitate hydratase	*Aco1*	30	30	1	0	5	4	8	14	13	16	15	13	31.9	98
Q99KI0	Aconitate hydratase, mitochondrial	*Aco2*	47	47	9	9	16	12	15	26	22	24	20	20	58.1	85
Q91V12-2;Q91V12-4;Q91V12;E9PYH2;Q91V12-3	Cytosolic acyl coenzyme A thioester hydrolase	*Acot7*	13	13	1	0	2	2	7	4	6	5	4	5	39.9	38
Q8VCW8	Acyl-CoA synthetase family member 2, mitochondrial	*Acsf2*	12	12	1	0	4	1	3	6	3	4	2	4	20.5	68
Q3UNX5;Q3UNX5-2	Acyl-coenzyme A synthetase ACSM3, mitochondrial	*Acsm3*	13	13	1	3	3	0	3	8	2	7	2	1	21.7	66
Q9QXG4;A2AQN4;A2AQN5;F7CU63;D6RHA7	Acetyl-coenzyme A synthetase, cytoplasmic	*Acss2*	10	10	0	0	1	1	1	3	4	8	8	8	18.5	79

P; Peptides, UP; Unique peptides, SC; Sequence coverage, MW: Molecular weight

P63260;B1ATY1;G3UZ07;G3UYG0;E9Q606;F8WGM8;F6WX90;E9Q3M9	Actin, cytoplasmic 2;Actin, cytoplasmic 2, N-terminally processed	*Actg1*	42	1	0	0	0	0	0	1	1	1	1	1	80	42
Q7TPR4;A1BN54;D3YY95	Alpha-actinin-1	*Actn1*	56	29	2	4	5	6	10	13	14	14	16	15	57.3	103
P57780;E9Q2W9;D3Z0L8;D3Z761;O88990	Alpha-actinin-4	*Actn4*	87	59	11	13	20	22	19	27	28	28	26	28	74	105
P61164	Alpha-centractin	*Actr1a*	9	5	0	1	2	2	3	3	3	3	2	3	33	43
Q8R5C5;E0CZD4;E0CYB4	Beta-centractin	*Actr1b*	9	5	0	2	2	1	1	3	4	3	3	3	34.6	42
P61161	Actin-related protein 2	*Actr2*	18	18	0	3	4	7	7	6	10	8	8	6	42.9	45
Q99JY9;Q641P0;Q641P0-2	Actin-related protein 3	*Actr3*	24	24	3	3	6	6	10	9	14	10	11	10	49.5	47
Q99JW2	Aminoacylase-1	*Acy1*	15	15	0	1	5	1	5	7	7	5	8	6	35.5	46
Q9QYB5-2;Q9QYB5	Gamma-adducin	*Add3*	7	7	0	0	2	1	1	2	3	1	2	2	13.9	75
P00329;E0CXV3	Alcohol dehydrogenase 1	*Adh1*	24	24	0	5	9	9	13	9	12	11	10	7	54.7	40
P28474;Q64437	Alcohol dehydrogenase class-3	*Adh5*	14	14	0	1	1	3	5	4	6	5	5	3	26.2	40
P55264;P55264-2	Adenosine kinase	*Adk*	7	7	0	0	1	2	6	4	5	3	4	4	21.1	40
P54822;E9Q242;E9Q3T7;E9Q0A0	Adenylosuccinate lyase	*Adsl*	14	14	0	0	1	1	1	3	2	5	3	2	30.2	55
O88312	Anterior gradient protein 2 homolog	*Agr2*	13	13	4	4	5	5	5	6	5	6	5	5	70.9	20
P50247;A2ALT5	Adenosylhomocysteinase	*Ahcy*	27	27	1	2	8	6	11	11	14	12	12	10	50.5	48
F8WGT1;Q68FL4;E9PV16;Q68FL4-2;F8WI65;H3BKT5;D3YX97;H3BL31;D3YYM7;E9PX77;F7ATQ6;D3Z2Q0	Adenosylhomocysteinase;Putative adenosylhomocysteinase 3	*Ahcyl2*	21	12	1	0	2	3	4	2	3	3	3	2	26.8	67
E9Q616;G5E8K8	Protein Ahnak	*Ahnak*	238	238	30	37	63	39	62	98	78	58	60	53	67.9	604
F7CVJ5;F7DBB3;E9PYB0	Protein Ahnak2	*Ahnak2*	6	6	2	0	3	1	2	3	3	1	3	4	35.6	115
P29699	Alpha-2-HS-glycoprotein	*Ahsg*	8	7	2	2	3	4	4	2	5	4	4	3	27.5	37
B1AU25;Q9Z0X1	Apoptosis-inducing factor 1, mitochondrial	*Aifm1*	18	18	0	1	5	2	2	9	5	6	6	5	33.9	66
P31230;Q3UZG4	Aminoacyl tRNA synthase complex-interacting multifunctional protein 1;Endothelial monocyte-activating polypeptide 2	*Aimp1*	15	15	1	0	1	0	6	3	2	2	3	3	53.5	34
Q9R0Y5;Q9R0Y5-2	Adenylate kinase isoenzyme 1	*Ak1*	5	5	2	3	3	2	1	1	2	3	3	2	34	22
Q9WTP6-2;Q9WTP6;F7BP55	Adenylate kinase 2, mitochondrial	*Ak2*	20	20	4	4	5	3	6	7	6	8	5	6	61.2	26
Q9WTP7;F6RP11	GTP:AMP phosphotransferase AK3, mitochondrial	*Ak3*	16	16	3	4	3	5	6	8	6	9	7	7	63.9	25
Q9JII6;B1AXW3	Alcohol dehydrogenase [NADP(+)]	*Akr1a1*	24	24	6	7	10	9	10	14	13	15	14	12	61.2	37
P45376;D3YVJ7	Aldose reductase	*Akr1b1*	21	21	4	5	6	9	14	11	14	11	11	11	54.4	36
P45377	Aldose reductase-related protein 2	*Akr1b8*	11	5	1	0	0	1	2	1	1	1	2	1	29.4	36
Q8VC28	Aldo-keto reductase family 1 member C13	*Akr1c13*	20	4	1	0	0	0	1	2	4	1	1	1	58.8	37
Q91WT7;E0CYR9;D3Z3P8;G3XA14;Q8K023-2;Q3UXL1;Q91WR5;Q8K023;P70694	3-alpha-hydroxysteroid dehydrogenase type 1	*Akr1c14*	14	14	2	2	3	5	2	5	4	6	6	5	47.4	37
G3X9Y6	Aldo-keto reductase family 1, member C19	*Akr1c19*	11	11	3	3	3	3	4	9	9	8	9	8	40.2	37
Q8CG76	Aflatoxin B1 aldehyde reductase member 2	*Akr7a2*	10	10	1	2	2	4	4	3	3	2	3	3	28.9	41
P10518	Delta-aminolevulinic acid dehydratase	*Alad*	8	8	1	0	1	1	2	5	5	3	5	3	29.4	36
P07724	Serum albumin	*Alb*	60	56	23	25	22	30	34	19	29	23	24	22	76.8	69

P; Peptides, UP; Unique peptides, SC; Sequence coverage, MW: Molecular weight

D3Z0B9;Q571I9;F6RQF0	Aldehyde dehydrogenase family 16 member A1	*Aldh16a1*	10	10	0	0	0	0	1	2	3	2	1	2	16.8	79
P24549	Retinal dehydrogenase 1	*Aldh1a1*	21	10	3	0	3	1	5	5	5	5	5	5	48.3	54
Q9CZS1;G3UYH1;G3UWP3;Q9JHW9	Aldehyde dehydrogenase X, mitochondrial	*Aldh1b1*	33	28	3	5	7	9	9	12	13	10	10	12	55.3	58
Q8R0Y6;D3Z6B9;Q8K009	Cytosolic 10-formyltetrahydrofolate dehydrogenase	*Aldh1l1*	51	51	10	11	15	10	18	22	29	24	24	24	55.1	99
P47738;D3YYF3	Aldehyde dehydrogenase, mitochondrial	*Aldh2*	38	35	11	9	13	10	12	18	17	21	14	17	60.1	57
Q9JLJ2;Q3U367	4-trimethylaminobutyraldehyde dehydrogenase	*Aldh9a1*	20	20	1	1	1	4	4	4	8	9	9	7	37	54
P05064;A6ZI44;D3YWI1;Q9CPQ9;A6ZI46;D3YV98;D3Z510;A6ZI47	Fructose-bisphosphate aldolase A;Fructose-bisphosphate aldolase	*Aldoa;Aldoart1*	43	33	14	11	13	15	14	16	17	17	17	16	79.1	39
Q91Y97	Fructose-bisphosphate aldolase B	*Aldob*	26	25	1	4	8	7	6	9	11	11	10	10	62.9	40
O08583;Q9JJW6-2;O08583-2;Q9JJW6;G3X9I4	THO complex subunit 4;RNA and export factor-binding protein 2	*Alyref;Refbp2;Alyref2*	9	9	0	3	4	2	2	5	3	3	4	5	31.4	27
Q8K3X6	Ankyrin repeat and SAM domain-containing protein 4B	*Anks4b*	13	13	1	0	0	2	0	3	6	6	2	3	25.3	48
O35381;D3Z7M9;D3YYE1;F6UFG6;Q64G17;E9Q148	Acidic leucine-rich nuclear phosphoprotein 32 family member A	*Anp32a*	14	11	2	1	3	4	3	4	5	5	4	6	38.5	29
P10107;A2BH40-4;A2BH40-2;A2BH40-3;A2BH40;E9QAQ7	Annexin A1	*Anxa1*	32	32	1	4	5	7	7	9	10	8	8	7	73.7	39
P97384;D3Z7U0	Annexin A11;Annexin	*Anxa11*	28	28	5	5	8	5	5	10	9	11	9	7	43.3	54
P07356;B0V2N7;B0V2N8;B0V2N5;REV__F6WNG1;REV__Q8BND4	Annexin A2	*Anxa2*	45	45	12	10	14	11	15	16	18	20	17	18	81.1	39
O35639	Annexin A3	*Anxa3*	36	36	7	7	8	9	8	13	17	13	10	13	78.6	36
P97429;D3Z0S1;F7ANV6	Annexin A4;Annexin	*Anxa4*	31	31	1	1	8	9	5	11	13	13	12	13	69	36
P48036	Annexin A5	*Anxa5*	27	27	2	3	4	9	11	6	6	8	2	8	56.1	36
Q07076	Annexin A7	*Anxa7*	18	18	3	2	4	2	1	10	9	8	9	7	35.4	50
O35643;Q5SVG5;Q5SVG4	AP-1 complex subunit beta-1	*Ap1b1*	18	9	0	0	0	0	0	2	3	3	1	1	23.9	104
P22892;Q8CBB7	AP-1 complex subunit gamma-1	*Ap1g1*	11	11	0	0	1	0	1	5	3	3	2	2	11.6	91
Q8R146-2;Q8R146	Acylamino-acid-releasing enzyme	*Apeh*	15	15	1	1	5	4	4	8	6	9	7	8	25.5	80
Q00623;CON__P15497	Apolipoprotein A-I;Truncated apolipoprotein A-I	*Apoa1*	25	25	6	11	10	11	10	8	10	12	11	8	65.2	31
P06728	Apolipoprotein A-IV	*Apoa4*	23	23	3	3	4	9	5	2	10	11	13	12	61.8	45
Q8K3G9;H3BL43;D3Z340;D6RFR7	DCC-interacting protein 13-beta	*Appl2*	12	12	0	0	2	3	1	2	3	4	4	4	19.6	74
Q5XJY5	Coatomer subunit delta	*Arcn1*	25	25	2	1	5	3	8	6	11	11	11	9	47.6	57
P84078;P61205;Q8BSL7;P84084;D3YV25;E9Q2C2	ADP-ribosylation factor 1;ADP-ribosylation factor 3;ADP-ribosylation factor 2	*Arf1;Arf3;Arf2*	6	4	0	0	1	1	0	3	1	3	3	3	41.4	21
P61750;E9Q798;F6UFB9	ADP-ribosylation factor 4	*Arf4*	5	3	0	0	2	0	0	2	2	2	2	2	33.9	20
Q9D8S3	ADP-ribosylation factor GTPase-activating protein 3	*Arfgap3*	9	9	1	0	1	1	1	3	3	3	3	2	18.4	57
Q5FWK3;A2AH25	Rho GTPase-activating protein 1	*Arhgap1*	15	15	0	1	2	1	2	5	5	5	5	5	36	50
Q99PT1	Rho GDP-dissociation inhibitor 1	*Arhgdia*	17	17	5	5	6	8	8	8	8	7	8	8	68.6	23
Q9WV32;F6THG2;F6VVE6;D3Z6S0;D3YVI5	Actin-related protein 2/3 complex subunit 1B	*Arpc1b*	21	20	1	1	2	5	5	6	6	5	5	5	45.4	41

P; Peptides, UP; Unique peptides, SC; Sequence coverage, MW: Molecular weight

Q9CVB6;D3YXG6	Actin-related protein 2/3 complex subunit 2	*Arpc2*	18	18	2	2	6	8	9	11	11	8	11	10	45	34
Q9JM76;H7BWZ3;D3Z2F7;D3Z2F8	Actin-related protein 2/3 complex subunit 3	*Arpc3*	9	9	4	1	2	2	3	3	5	6	3	4	38.8	21
P59999;E9PWA7	Actin-related protein 2/3 complex subunit 4	*Arpc4*	12	12	0	0	4	3	5	5	4	6	5	4	45.8	20
Q9CPW4;Q3UA72;E9Q2K4	Actin-related protein 2/3 complex subunit 5	*Arpc5*	12	12	3	3	3	1	7	4	4	3	4	3	72.8	16
Q9D0A3	Arpin	*Arpin*	8	8	1	0	2	2	2	2	2	2	2	2	28.3	25
Q91WU5	Arsenite methyltransferase	*As3mt*	15	15	2	1	3	2	2	4	5	9	6	4	40.4	42
E0CXM2;Q91YI0;E0CY49;F7D439;E0CYV3	Argininosuccinate lyase	*Asl*	13	13	0	0	0	0	2	1	3	5	6	4	29.9	49
P16460;J3QNG0	Argininosuccinate synthase	*Ass1;Gm5424*	9	9	1	2	2	2	5	3	3	2	2	4	21.1	47
Q9CWJ9	Bifunctional purine biosynthesis protein PURH;Phosphoribosylaminoimidazolecarboxamide formyltransferase;IMP cyclohydrolase	*Atic*	27	27	3	5	8	11	13	8	14	12	13	10	47.6	64
O08997	Copper transport protein ATOX1	*Atox1*	5	5	2	2	2	2	4	2	3	3	2	2	85.3	7
Q9Z1W8	Potassium-transporting ATPase alpha chain 2	*Atp12a*	45	37	8	10	5	9	3	17	22	15	12	12	41.2	115
Q8VDN2;Q91WH7;Q64436;E9QNX7	Sodium/potassium-transporting ATPase subunit alpha-1	*Atp1a1*	62	44	12	11	15	13	16	28	19	22	23	23	51.2	113
P14094	Sodium/potassium-transporting ATPase subunit beta-1	*Atp1b1*	17	17	3	3	5	4	4	9	8	6	5	4	39.8	35
Q03265;D3Z6F5;D6RJ16	ATP synthase subunit alpha, mitochondrial;ATP synthase subunit alpha	*Atp5a1*	49	49	20	19	20	17	19	25	24	20	22	22	61.8	60
P56480	ATP synthase subunit beta, mitochondrial	*Atp5b*	29	29	12	10	13	13	15	17	18	15	16	16	52.6	56
Q8C2Q8;A2AKU9;Q91VR2;A2AKV1;A2AKV2;A2AKV3;A2AKV0	ATP synthase subunit gamma;ATP synthase subunit gamma, mitochondrial	*Atp5c1*	19	19	7	6	9	8	6	9	10	10	7	7	48.9	30
Q9D3D9	ATP synthase subunit delta, mitochondrial	*Atp5d*	4	4	1	1	1	2	2	2	2	2	2	3	20.2	18
Q9CQQ7	ATP synthase subunit b, mitochondrial	*Atp5f1*	17	17	2	1	2	0	3	9	6	4	4	4	38.7	29
Q06185;Q8BTB6	ATP synthase subunit e, mitochondrial	*Atp5i*	4	4	1	1	2	1	1	2	2	3	2	2	38	8
P97450;E9QAD6	ATP synthase-coupling factor 6, mitochondrial	*Atp5j*	6	6	1	1	1	1	2	4	5	5	4	5	48.1	12
Q9DB20;F6XVM5;F7D3P8	ATP synthase subunit O, mitochondrial	*Atp5o*	17	17	4	3	4	4	3	6	5	6	5	5	66.2	23
P50516;P50516-2;D3Z1B9;D3YWH3;D3YZ23	V-type proton ATPase catalytic subunit A	*Atp6v1a*	20	20	3	1	1	2	4	6	6	7	8	7	29.7	68
P62814;Q91YH6	V-type proton ATPase subunit B, brain isoform	*Atp6v1b2*	19	19	0	2	3	3	3	4	4	5	5	1	39.1	57
P50518;Q9D593	V-type proton ATPase subunit E 1	*Atp6v1e1*	12	12	0	4	3	1	2	4	3	3	5	0	44.7	26
E9PV44;O35143	ATPase inhibitor, mitochondrial	*Atpif1*	13	13	1	2	2	2	2	3	2	3	2	3	74.3	9
P01887	Beta-2-microglobulin	*B2m*	4	4	2	1	1	2	1	2	3	1	2	2	24.4	14
Q9DBJ3	Brain-specific angiogenesis inhibitor 1-associated protein 2-like protein 1	*Baiap2l1*	17	16	0	1	2	5	3	5	6	6	3	3	35.6	57

P; Peptides, UP; Unique peptides, SC; Sequence coverage, MW: Molecular weight

O54962	Barrier-to-autointegration factor	*Banf1*	6	6	0	0	1	1	2	2	1	3	2	2	42.7	10
A2AVX1;E9Q8Q5;Q80YN3;F7BNZ5	Breast carcinoma-amplified sequence 1 homolog	*Bcas1*	21	21	8	3	6	8	6	9	12	11	11	8	39.3	61
Q80XN0;D3Z2Y8	D-beta-hydroxybutyrate dehydrogenase, mitochondrial	*Bdh1*	12	12	1	3	2	4	2	7	6	6	3	3	36.2	38
Q8R016;E9Q6V3;E9PY26;E9PZH4;E9QA53	Bleomycin hydrolase	*Blmh*	11	11	0	1	0	3	3	2	2	4	2	2	21.3	53
Q9CY64;A2ASB8;A2ASB7;A2ASB1	Biliverdin reductase A	*Blvra*	10	10	0	1	3	3	3	2	1	4	5	2	30.8	34
Q923D2;E9PZC3;E9PZC4	Flavin reductase (NADPH)	*Blvrb*	8	8	2	4	2	4	2	2	2	3	3	3	43.2	22
Q9Z0S1;D3Z0E6;D3Z5X0	3(2),5-bisphosphate nucleotidase 1	*Bpnt1*	12	12	3	1	6	3	7	4	8	8	6	6	49	33
K3W4Q8;P18572-2;P18572;J3QP71	Basigin	*Bsg*	12	12	1	3	5	5	4	8	6	8	4	5	44.5	24
Q9CQH7	Transcription factor BTF3 homolog 4	*Btf3l4*	2	2	1	0	2	2	2	2	2	2	2	2	14.6	17
Q9CQC6	Basic leucine zipper and W2 domain-containing protein 1	*Bzw1*	12	12	0	0	0	2	4	2	2	2	5	4	22.4	48
P01027;P01027-2;H3BKW9;H3BL60	Complement C3;Complement C3 beta chain;Complement C3 alpha chain;C3a anaphylatoxin;Acylation stimulating protein;Complement C3b alpha chain;Complement C3c alpha chain fragment 1;Complement C3dg fragment;Complement C3g fragment;Complement C3d fragment;Complement C3f fragment;Complement C3c alpha chain fragment 2	*C3*	64	60	11	9	5	16	14	6	29	8	19	8	33.9	186
P13634;D3YYQ4	Carbonic anhydrase 1	*Ca1*	18	18	10	11	12	11	13	14	14	13	14	14	80.8	28
P00920	Carbonic anhydrase 2	*Ca2*	16	16	8	11	10	10	8	11	11	11	11	11	67.3	29
Q64444;F6ST32	Carbonic anhydrase 4	*Ca4;Car4*	13	13	7	7	7	8	5	5	7	5	6	4	36.7	34
Q06138;D3YV52;D3Z704;Q9DB16-2	Calcium-binding protein 39	*Cab39*	12	11	0	0	1	0	3	3	6	5	4	3	28.7	40
Q9CXW3	Calcyclin-binding protein	*Cacybp*	16	16	2	0	1	2	3	3	2	5	6	4	52.4	27
P62204;Q3UKW2;Q9D6P8;G3UX57;P20801	Calmodulin	*Calm1*	15	15	6	6	6	6	6	7	8	8	8	8	61.1	17
P14211	Calreticulin	*Calr*	37	37	5	5	6	13	16	13	12	16	13	10	69	48
Q6ZQ38;D3YWC5;Q6ZQ73	Cullin-associated NEDD8-dissociated protein 1	*Cand1*	26	26	0	1	2	0	6	9	8	11	10	6	23.6	136
P35564	Calnexin	*Canx*	17	17	1	2	4	2	5	10	3	3	2	3	26.9	67
P40124;B1ARS0;D3YTR7;Q9CYT6	Adenylyl cyclase-associated protein 1	*Cap1*	36	36	8	10	11	13	13	13	19	17	15	17	70.5	52
O35350	Calpain-1 catalytic subunit	*Capn1*	19	19	1	0	2	0	3	6	5	6	6	4	29	82
O08529	Calpain-2 catalytic subunit	*Capn2*	12	12	0	0	2	2	0	4	6	7	5	4	19.6	80
D3YW48;O88456;Q9D7J7	Calpain small subunit 1	*Capns1*	8	8	0	0	2	2	2	3	2	3	4	2	41.9	25
Q5RKN9;P47753	F-actin-capping protein subunit alpha-1	*Capza1*	15	12	3	5	3	2	5	7	8	7	7	7	72.7	33
P47754;D6RCW7	F-actin-capping protein subunit alpha-2	*Capza2*	14	11	1	2	2	1	2	9	9	9	9	7	70.3	33
A2AMW0;P47757-2;A2AMV7;P47757;F7CAZ6;F6QJN8;F6YHZ8	F-actin-capping protein subunit beta	*Capzb*	21	21	4	5	8	5	8	8	11	9	8	8	58.8	29
P29452	Caspase-1;Caspase-1 subunit p20;Caspase-1 subunit p10	*Casp1*	24	24	1	2	3	3	9	4	5	7	6	7	48.3	46

P; Peptides, UP; Unique peptides, SC; Sequence coverage, MW: Molecular weight

P70677	Caspase-3;Caspase-3 subunit p17;Caspase-3 subunit p12	*Casp3*	8	8	0	0	0	1	3	2	2	5	4	2	32.1	31
P97864	Caspase-7;Caspase-7 subunit p20;Caspase-7 subunit p11	*Casp7*	14	14	0	1	2	2	2	2	3	4	4	3	40.3	34
O89110	Caspase-8;Caspase-8 subunit p18;Caspase-8 subunit p10	*Casp8*	15	15	0	1	2	3	4	4	3	5	5	3	26.9	55
P51125-3;P51125-2;P51125;Q8CE80;P51125-6;P51125-7;P51125-5;P51125-4	Calpastatin	*Cast*	21	21	6	2	5	5	5	5	5	4	4	5	31.9	80
P48758	Carbonyl reductase [NADPH] 1	*Cbr1*	34	33	7	8	9	14	13	14	14	15	12	12	90.6	31
Q8K354	Carbonyl reductase [NADPH] 3	*Cbr3*	15	14	2	3	5	7	7	4	6	5	5	5	54.5	31
P80314	T-complex protein 1 subunit beta	*Cct2*	33	33	3	5	9	9	11	10	10	7	14	10	50.3	57
P80318;E9Q133;Q3U0I3;F6Q609;F6ZVG8	T-complex protein 1 subunit gamma	*Cct3*	30	30	1	4	7	9	12	8	11	9	11	10	56.9	61
P80315;G5E839;G3UYW5;G3UXF3;G3UXG2	T-complex protein 1 subunit delta	*Cct4*	26	26	0	0	3	2	7	4	3	3	3	3	46.9	58
P80316;E0CZA1	T-complex protein 1 subunit epsilon	*Cct5*	31	31	2	4	7	6	11	4	2	6	4	4	46.4	60
P80317;E9QPA6;Q61390;B1AT05	T-complex protein 1 subunit zeta	*Cct6a*	25	25	3	5	10	7	7	9	9	12	11	11	37.7	58
P80313	T-complex protein 1 subunit eta	*Cct7*	22	22	2	3	5	6	8	5	4	8	8	4	37.3	60
P42932;H3BL49;H3BJB6;H3BLL1;H3BKG2;H3BKR8	T-complex protein 1 subunit theta	*Cct8*	31	31	0	2	2	3	12	10	6	13	12	13	50	60
Q9JLQ0	CD2-associated protein	*Cd2ap*	27	27	3	3	5	5	4	7	11	10	6	10	43.2	70
Q61081	Hsp90 co-chaperone Cdc37	*Cdc37*	16	16	2	0	0	2	1	4	5	4	4	4	39.3	45
P60766;P60766-1	Cell division control protein 42 homolog	*Cdc42*	7	7	0	0	1	1	2	4	4	1	2	2	30.9	21
P09803	Cadherin-1;E-Cad/CTF1;E-Cad/CTF2;E-Cad/CTF3	*Cdh1*	28	28	2	3	6	4	5	7	7	10	10	7	31.6	98
Q9R100;A2AKS7	Cadherin-17	*Cdh17*	34	34	8	6	12	10	12	23	20	20	18	19	40.3	92
Q4VAA2-2;Q4VAA2;F8WGL9	Protein CDV3	*Cdv3*	9	9	1	2	2	4	5	1	1	3	3	1	41.5	24
P31809-2;Q925P3;P31809;Q3LFS9;P31809-3;Q925P2;E9QLI9	Carcinoembryonic antigen-related cell adhesion molecule 1	*Ceacam1*	8	8	3	0	2	3	2	2	6	2	3	2	13.1	50
P23953;D3Z5G7;F6XTA4;E9PYP1;Q8VCC2	Carboxylesterase 1C	*Ces1c*	15	13	2	1	3	3	2	3	9	6	9	5	28.5	61
Q91WG0;D3YWM6	Acylcarnitine hydrolase	*Ces2c;Ces2d-ps*	16	9	3	2	0	2	2	5	5	2	2	3	26.4	62
P18760;F8WGL3	Cofilin-1	*Cfl1*	28	21	3	6	6	7	6	9	10	6	9	9	94.6	19
P45591	Cofilin-2	*Cfl2*	15	8	0	1	2	4	1	2	2	3	4	4	69.3	19
E9QMC1;D3YUW7;P59242;P59242-2	Cingulin	*Cgn*	42	42	7	1	9	4	2	6	6	4	4	5	31.8	137
Q9CRB9;Q9D9P1;D3Z0L4;F6QFL0	Coiled-coil-helix-coiled-coil-helix domain-containing protein 3, mitochondrial	*Chchd3*	15	15	2	3	3	4	4	4	5	5	4	4	45.8	26
Q8BJ64	Choline dehydrogenase, mitochondrial	*Chdh*	22	22	4	1	3	1	3	11	7	9	5	2	32.9	66
Q9D8B3	Charged multivesicular body protein 4b	*Chmp4b*	16	13	1	1	1	1	4	4	5	7	6	4	54.9	25
Q91WS0	CDGSH iron-sulfur domain-containing protein 1	*Cisd1*	5	5	0	0	2	2	2	3	2	4	2	3	46.3	12
Q04447	Creatine kinase B-type	*Ckb*	22	20	8	10	11	12	11	11	13	13	13	12	63.5	43
P07310	Creatine kinase M-type	*Ckm*	18	15	3	4	5	7	8	5	11	9	11	10	56.2	43

P; Peptides, UP; Unique peptides, SC; Sequence coverage, MW: Molecular weight

P30275;A2ARP5;B0R0F0;B0R0E9;B0R0E8;Q6P8J7	Creatine kinase U-type, mitochondrial	*Ckmt1*	44	43	16	17	21	20	19	19	20	20	18	20	69.1	47
Q9D7Z6	Calcium-activated chloride channel regulator 1	*Clca1*	52	52	31	31	35	37	36	0	0	0	0	0	51.5	100
Q9Z0G9-2;Q9Z0G9	Claudin-3	*Cldn3*	5	5	1	0	2	0	0	3	2	3	2	2	19.1	21
Q9Z1Q5	Chloride intracellular channel protein 1	*Clic1*	14	14	3	2	4	3	3	8	8	8	5	6	56.4	27
Q9QYB1	Chloride intracellular channel protein 4	*Clic4*	8	8	1	1	1	2	2	0	3	3	5	3	32.4	29
Q8BXK9	Chloride intracellular channel protein 5	*Clic5*	10	9	1	2	3	1	3	5	4	3	4	4	41.8	28
Q6IRU5-2;Q6IRU5;F7BHJ0;Q6IRU5-3	Clathrin light chain B	*Cltb*	16	16	2	3	4	2	4	5	6	5	3	5	40.3	23
Q68FD5;Q5SXR6;F6Z1R4	Clathrin heavy chain 1	*Cltc*	55	55	4	5	15	10	9	34	28	26	26	27	30.7	192
Q99KK2;Q99KK2-2	N-acylneuraminate cytidylyltransferase	*Cmas*	16	16	1	2	5	3	4	4	6	6	3	4	27.8	48
Q8R1G2;E0CXT6;E0CXH4	Carboxymethylenebutenolidase homolog	*Cmbl*	4	4	0	0	1	0	0	3	2	4	2	3	16.7	28
Q9DBP5	UMP-CMP kinase	*Cmpk1*	25	25	3	6	7	10	8	13	12	13	11	11	73.5	22
Q9D1A2	Cytosolic non-specific dipeptidase	*Cndp2*	23	23	6	7	2	9	8	11	10	8	10	12	44.2	53
Q08091;Q08091-2	Calponin-1	*Cnn1*	14	14	4	6	6	9	8	11	11	8	12	13	41.8	33
Q69ZF7;Q3TWN3-2;Q3TWN3;Q32NY4-2;Q32NY4	Metal transporter CNNM4	*Cnnm4*	16	16	1	1	3	1	1	8	4	1	3	3	20.1	87
Q9QXT0	Protein canopy homolog 2	*Cnpy2*	10	10	0	1	2	4	4	3	3	3	3	3	55.5	21
Q8CIE6;F8WHL2;F6XJN3	Coatomer subunit alpha;Xenin;Proxenin	*Copa*	44	44	1	1	5	4	8	22	17	21	19	16	36.3	138
Q9JIF7	Coatomer subunit beta	*Copb1*	28	28	0	1	3	1	2	10	7	13	8	11	31.8	107
O55029	Coatomer subunit beta	*Copb2*	27	27	3	0	5	7	6	10	10	9	11	12	31.6	102
O89079;D3Z315;E9Q6I5;F6YFR7;F6XIG5	Coatomer subunit epsilon	*Cope*	12	12	0	2	2	1	3	4	3	6	4	4	39.6	35
Q9QZE5;Q7TNQ1	Coatomer subunit gamma-1	*Copg1*	19	17	1	1	2	3	4	8	3	7	6	5	26.5	98
P61202;P61202-2;A2AQE4	COP9 signalosome complex subunit 2	*Cops2*	11	11	0	0	1	0	0	3	3	2	3	3	24.8	52
O88544;F6QTS1;D3Z1R9;D3YV99	COP9 signalosome complex subunit 4	*Cops4*	9	9	0	0	1	0	0	2	3	3	4	4	29.6	46
O89053;G3UYK8;D3YW57;G3UX53;D3YXM2	Coronin-1A;Coronin	*Coro1a*	15	15	0	0	1	0	7	4	1	2	3	3	27.3	51
Q9WUM3;D3YUG6	Coronin-1B	*Coro1b*	23	22	5	6	8	6	9	6	10	8	8	8	34.7	54
Q9WUM4;E9PX03;E9PZJ0;E9PVJ1;Q920M5-3;B9EIZ7;Q920M5-2;Q920M5;Q920M5-4	Coronin-1C	*Coro1c*	26	25	2	2	7	5	11	4	7	5	5	6	39.2	53
Q8C0P5;B1AVH5	Coronin-2A;Coronin	*Coro2a*	9	8	0	1	1	1	1	2	4	3	2	2	14.9	60
Q9CQI6	Coactosin-like protein	*Cotl1*	14	14	4	0	2	3	3	4	4	5	4	3	64.8	16
P19783;D6RG40	Cytochrome c oxidase subunit 4 isoform 1, mitochondrial	*Cox4i1*	14	14	2	2	2	2	3	7	7	4	5	5	55.6	20
P12787	Cytochrome c oxidase subunit 5A, mitochondrial	*Cox5a*	7	7	2	4	3	2	3	3	3	3	3	3	31.5	16
P19536;Q9D881	Cytochrome c oxidase subunit 5B, mitochondrial	*Cox5b*	7	7	3	3	3	3	3	4	4	4	4	4	35.9	14
P43024;Q9DCW5	Cytochrome c oxidase subunit 6A1, mitochondrial;Cytochrome c oxidase subunit 6A, mitochondrial	*Cox6a1*	3	3	0	0	0	0	0	3	3	2	3	2	40.5	12
P56391	Cytochrome c oxidase subunit 6B1	*Cox6b1*	7	7	3	0	0	2	1	3	4	3	2	3	50	10
Q9CPQ1;D3Z6E1	Cytochrome c oxidase subunit 6C	*Cox6c*	9	9	2	2	4	3	2	3	3	3	3	3	61.8	8
P17665;G3X8T4;J3QNB4	Cytochrome c oxidase subunit 7C, mitochondrial	*Cox7c;Gm20091*	2	2	1	1	1	2	0	2	2	2	2	2	28.6	7

P; Peptides, UP; Unique peptides, SC; Sequence coverage, MW: Molecular weight

Q8BT60;B7ZCP8;B7ZCP7;B7ZCP6;B7ZCP5;E9Q806;A3KGK7;Q9D6C8;B7ZCP4;Q8C166;Q1RLL3;Q3UYN2;Q9Z140;Q8BLR2;Q0VE82;Q9DC53;B2RS65;Q8JZW4	Copine-3	*Cpne3*	9	8	0	0	1	1	0	3	2	2	2	1	15	60
Q8BFS6;Q8BFS6-2;Q8BFS6-3;Q8BFS6-4;D3Z7F7;Q8BFS6-5	Calcineurin-like phosphoesterase domain-containing protein 1	*Cpped1*	6	6	1	1	0	1	2	2	4	2	2	2	19.9	35
P52825;A2A8E7;A2A8E8;A2A8E9	Carnitine O-palmitoyltransferase 2, mitochondrial	*Cpt2*	14	14	0	0	1	0	1	11	2	4	3	1	20.2	74
P63254	Cysteine-rich protein 1	*Crip1*	5	5	3	3	3	3	4	3	4	3	3	3	64.9	9
Q9DCT8	Cysteine-rich protein 2	*Crip2*	5	5	2	1	1	2	2	2	3	2	4	3	31.7	23
Q64010;Q8JZR2;Q64010-2;Q5ND50;F7D232;Q3TQV3	Adapter molecule crk	*Crk*	15	15	0	0	2	1	0	3	5	2	5	2	50	34
Q8CJ40-2;Q8CJ40-3;Q8CJ40	Rootletin	*Crocc*	2	2	1	1	1	2	2	2	2	2	2	2	0.8	208
Q99KP3	Lambda-crystallin homolog	*Cryl1*	12	12	3	3	6	8	6	7	7	9	7	7	41.7	35
Q9CZU6;Q80X68	Citrate synthase, mitochondrial	*Cs*	19	19	2	2	8	4	6	9	10	11	9	7	30.8	52
Q9DBE0	Cysteine sulfinic acid decarboxylase	*Csad*	9	9	0	0	0	0	0	3	1	4	4	2	16.8	55
Q60737;A2ANR6;Q6NSS6	Casein kinase II subunit alpha	*Csnk2a1*	12	12	1	0	1	0	0	2	3	6	2	3	40.2	45
P97315	Cysteine and glycine-rich protein 1	*Csrp1*	11	11	4	5	5	8	8	7	7	7	7	7	64.2	21
Q62426	Cystatin-B	*Cstb*	10	10	1	2	3	2	2	3	3	3	3	4	58.2	11
P26231;Q61301-2;Q61301-3;Q61301;E0CXB9;Q8BS72;Q65CL1	Catenin alpha-1	*Ctnna1*	50	50	3	3	11	7	6	22	19	14	11	15	50.1	100
Q02248;E9Q6A9;F6QZ47;F7BAC9;D3YUH4;F7CRC6;D3Z7S6;D3Z5Q1;E9PW26	Catenin beta-1	*Ctnnb1*	22	19	0	1	1	1	2	7	6	2	2	5	33.2	85
G3X9V2;P30999;E9Q8Z6;E9Q8Z8;P30999-2;P30999-3;E9Q8Z5;E9Q8Z4;E9Q8Z9;D3Z2H2;E9Q901;E9Q904;E9Q986;D3Z7H6;E9Q907;E9Q905;E9Q903;E9Q906;D3Z2H7	Catenin delta-1	*Ctnnd1*	28	28	0	5	7	2	2	13	9	5	4	7	33.6	104
Q921L6;Q60598	Src substrate cortactin	*Cttn*	26	26	6	5	8	3	6	11	13	10	8	10	50.5	57
Q9D0M3-2;Q9D0M3	Cytochrome c1, heme protein, mitochondrial	*Cyc1*	10	10	2	2	4	4	5	6	6	6	5	5	37.6	29
P62897;G3UWG1;CON__P62894;P00015	Cytochrome c, somatic	*Cycs;Gm10108*	15	15	2	3	6	7	6	5	4	3	3	5	69.5	12
Q7TMB8;Q7TMB8-2;Q5SQX6;F6QD74;E9PZS0;G3UZI5	Cytoplasmic FMR1-interacting protein 1;Cytoplasmic FMR1-interacting protein 2	*Cyfip1;Cyfip2*	20	20	0	1	1	0	2	5	5	7	8	5	16.5	145
Q9D172	ES1 protein homolog, mitochondrial	*D10Jhu81e*	5	5	2	3	4	3	3	4	2	3	2	3	22.9	28
Q99LF4	tRNA-splicing ligase RtcB homolog	*D10Wsu52e*	23	23	1	3	8	4	6	6	10	8	8	10	45.9	55
Q922B2;Q8BJY7	Aspartate--tRNA ligase, cytoplasmic	*Dars*	25	25	0	0	5	1	8	10	7	11	11	6	52.3	57
P31786;F6WUQ1;Q4VWZ5;F6ZM12;D3Z563	Acyl-CoA-binding protein	*Dbi*	10	10	2	1	2	1	2	2	2	2	2	2	72.4	10
Q9DAR7;Q3TBW9;D6RFQ0	m7GpppX diphosphatase	*Dcps*	11	11	0	0	0	0	1	4	3	3	5	5	39.1	39

P; Peptides, UP; Unique peptides, SC; Sequence coverage, MW: Molecular weight

E9Q586;E9Q3M3;O08788-2;O08788;D3YX34;D3Z2M9;D3YYG9	Dynactin subunit 1	*Dctn1*	35	35	1	1	3	1	4	4	6	5	6	5	27.8	137
Q99KJ8	Dynactin subunit 2	*Dctn2*	28	28	3	3	4	5	4	6	9	10	10	9	64.2	44
Q3U1J4	DNA damage-binding protein 1	*Ddb1*	28	28	0	0	4	0	1	4	1	3	5	3	24.8	127
O88533;Q5SUV9	Aromatic-L-amino-acid decarboxylase	*Ddc*	10	10	1	1	2	0	1	4	6	3	6	5	22.7	54
O35215;G3UZN1	D-dopachrome decarboxylase	*Ddt*	8	4	1	1	2	1	1	3	2	3	3	3	83.1	13
Q91VR5	ATP-dependent RNA helicase DDX1	*Ddx1*	17	17	1	0	1	3	1	7	3	5	4	4	24.9	82
Q9Z1N5;G3UXI6	Spliceosome RNA helicase Ddx39b	*Ddx39b*	15	6	0	0	2	2	3	0	1	1	2	1	32.7	49
Q62167;P16381;Q61496;Q3V086	ATP-dependent RNA helicase DDX3X;Putative ATP-dependent RNA helicase Pl10	*Ddx3x;D1Pas1*	17	3	1	1	1	0	1	1	1	1	1	1	28.4	73
Q61656;Q8BTS0;B1ARB9;B1ARB8;B1ARC0	Probable ATP-dependent RNA helicase DDX5	*Ddx5*	22	15	1	2	2	3	1	9	5	6	5	8	33.6	69
Q9CQ62	2,4-dienoyl-CoA reductase, mitochondrial	*Decr1*	9	9	2	1	0	0	2	3	2	2	2	3	32.5	36
P31001	Desmin	*Des*	45	37	7	7	19	16	16	17	17	21	16	24	62.7	53
Q3U0B3;F2Z445	Dehydrogenase/reductase SDR family member 11	*Dhrs11*	7	7	1	1	0	0	1	2	5	1	2	2	30.8	28
E9QNN1;O70133;O70133-2;O70133-3	ATP-dependent RNA helicase A	*Dhx9*	20	20	1	1	4	1	1	7	3	4	2	4	16.5	150
O08808;E9PXV7;E9PV41;Q6NS79;D3Z074	Protein diaphanous homolog 1	*Diaph1;Diap1*	21	21	0	0	2	2	3	3	3	4	2	3	15.8	139
Q8BMF4	Dihydrolipoyllysine-residue acetyltransferase component of pyruvate dehydrogenase complex, mitochondrial	*Dlat*	16	16	2	2	5	3	4	8	3	2	3	3	26.3	68
O08749	Dihydrolipoyl dehydrogenase, mitochondrial	*Dld*	21	21	5	5	5	4	9	5	6	7	4	6	39.5	54
Q9D2G2;Q9D2G2-2	Dihydrolipoyllysine-residue succinyltransferase component of 2-oxoglutarate dehydrogenase complex, mitochondrial	*Dlst*	15	15	3	3	1	2	3	8	6	6	4	5	28.6	49
Q3T9X3;P39054-2;Q3TCR7;P39054;G3X9G4;F8WIV5;G3UZZ3;P39053-5;P39053-3;P39053-2;P39053-6;P39053-4;P39053;G3UXX2;G3UY64;D6RH60;F6W8Z8	Dynamin-2	*Dnm2*	16	11	0	0	3	4	0	5	5	6	4	5	19.7	97
Q9Z2W0	Aspartyl aminopeptidase	*Dnpep*	11	11	0	1	4	2	0	6	7	5	7	5	27.5	52
Q99KK7	Dipeptidyl peptidase 3	*Dpp3*	24	24	1	1	2	3	4	4	5	6	8	4	33.5	83
O08553;Q3TMU8;O35098	Dihydropyrimidinase-related protein 2	*Dpysl2*	26	21	4	4	4	8	8	10	13	13	14	12	57	62
O55111;Q811I1;Q8VCE3	Desmoglein-2	*Dsg2*	30	30	1	1	4	1	6	11	6	10	6	8	32.2	122
E9Q557;E9PZW0	Desmoplakin	*Dsp*	131	131	6	4	15	5	4	36	15	13	7	3	41.2	333
Q9R0P5	Destrin	*Dstn*	21	17	6	6	9	10	11	7	9	11	7	7	83.6	19
Q9D7X3;B1AQF4;H3BKL8;Q3V2Y9;H3BKD1	Dual specificity protein phosphatase 3	*Dusp3*	4	4	0	4	1	1	0	2	2	2	2	2	26.5	20
Q9JHU4;F6ZX84	Cytoplasmic dynein 1 heavy chain 1	*Dync1h1*	150	150	4	2	20	9	16	59	57	54	51	39	31.5	532
Q8BH95;F6T930	Enoyl-CoA hydratase, mitochondrial	*Echs1*	16	16	4	5	5	1	5	6	5	7	4	4	40.7	31
P10126;D3YZ68;D3Z3I8;P62631	Elongation factor 1-alpha 1	*Eef1a1*	40	40	12	13	18	19	22	17	19	19	18	16	52.2	50

P; Peptides, UP; Unique peptides, SC; Sequence coverage, MW: Molecular weight

O70251;G3UX43;G3UZ47	Elongation factor 1-beta	*Eef1b*	11	9	0	2	2	2	3	6	3	3	5	5	46.7	25
Q9D8N0	Elongation factor 1-gamma	*Eef1g*	22	22	1	1	3	5	5	13	10	13	12	12	36.4	50
P58252;G3UXK8	Elongation factor 2	*Eef2*	57	55	8	13	15	20	21	26	31	30	27	26	50.2	95
Q8C845;Q9D8Y0;Q9D4J1	EF-hand domain-containing protein D2	*Efhd2*	22	22	2	3	3	5	4	5	8	6	5	7	62.9	27
Q8BH64;D3Z7U7	EH domain-containing protein 2	*Ehd2*	12	11	1	3	4	3	5	3	4	3	4	5	25.2	61
Q9EQP2	EH domain-containing protein 4	*Ehd4*	12	11	0	2	1	1	1	3	2	5	3	4	26.6	61
Q6ZWX6	Eukaryotic translation initiation factor 2 subunit 1	*Eif2s1*	15	15	1	1	3	3	3	4	3	4	4	3	37.8	36
Q99L45;J3QPC6;E0CXJ3	Eukaryotic translation initiation factor 2 subunit 2	*Eif2s2;Gm9892*	20	20	2	2	4	2	6	8	8	9	5	7	43.8	38
Q9Z0N1;A2AAW9	Eukaryotic translation initiation factor 2 subunit 3, X-linked	*Eif2s3x*	15	4	0	1	2	0	1	1	2	1	2	1	36.2	51
P23116	Eukaryotic translation initiation factor 3 subunit A	*Eif3a*	37	37	0	1	3	2	5	6	5	6	6	7	30.1	162
Q8JZQ9	Eukaryotic translation initiation factor 3 subunit B	*Eif3b*	21	21	0	1	2	2	3	7	1	7	4	6	25.5	91
Q8R1B4	Eukaryotic translation initiation factor 3 subunit C	*Eif3c*	17	17	0	2	3	3	6	2	4	3	3	2	17.2	106
Q9QZD9;A2AE03	Eukaryotic translation initiation factor 3 subunit I	*Eif3i*	12	12	0	1	2	1	1	4	1	4	4	3	43.1	36
Q3UGC7;Q66JS6	Eukaryotic translation initiation factor 3 subunit J-A;Eukaryotic translation initiation factor 3 subunit J-B	*Eif3j1;Eif3j2*	10	10	0	0	1	0	1	2	2	2	3	2	35.6	29
P60843;D6RJ60;D6RJ11	Eukaryotic initiation factor 4A-I	*Eif4a1*	29	15	2	2	3	4	4	6	4	7	4	4	48.5	46
P10630;P10630-2;Q8BTU6;E9Q561	Eukaryotic initiation factor 4A-II	*Eif4a2*	25	11	0	0	1	1	1	3	3	4	4	3	41.5	46
Q8BGD9	Eukaryotic translation initiation factor 4B	*Eif4b*	23	23	0	2	2	5	8	5	6	5	6	6	35.8	69
Q9WUK2-2;Q9WUK2	Eukaryotic translation initiation factor 4H	*Eif4h*	12	12	0	0	2	2	2	2	3	3	3	3	48.2	25
P59325	Eukaryotic translation initiation factor 5	*Eif5*	14	14	0	0	1	0	2	4	2	4	5	3	28	49
P63242;Q8BGY2;J3QPS8	Eukaryotic translation initiation factor 5A-1;Eukaryotic translation initiation factor 5A-2	*Eif5a;Eif5a2*	11	11	3	2	2	4	4	3	4	4	4	4	57.1	17
O55135;A6PWZ2;D6RG53;D6RJJ3;B1AZQ4	Eukaryotic translation initiation factor 6	*Eif6*	7	7	1	1	2	1	4	3	4	3	3	3	26.5	27
P70372	ELAV-like protein 1	*Elavl1*	13	13	1	1	2	1	2	4	3	2	1	4	39	36
D3YWS2;Q7TNG5;Q7TNG5-2;E9QK48;D6RGM3	Echinoderm microtubule-associated protein-like 2	*Eml2*	14	14	2	1	2	2	0	5	7	5	6	5	24.3	67
P17182;Q6PHC1;B0QZL1;B1ARR7;B1ARR6	Alpha-enolase;Enolase	*Eno1*	48	45	12	14	13	13	15	15	16	17	15	14	79.7	47
P21550;Q5SX59;J3QPZ9;Q5SX60;Q5SX61	Beta-enolase	*Eno3*	28	25	6	7	5	7	7	7	7	9	8	6	59	47
Q99JW5	Epithelial cell adhesion molecule	*Epcam*	18	18	3	3	3	4	4	7	6	6	4	8	41.9	35
Q8R0W0	Epiplakin	*Eppk1*	50	47	4	3	5	5	5	17	10	7	6	5	35.2	723
Q8CGC7	Bifunctional glutamate/proline--tRNA ligase;Glutamate--tRNA ligase;Proline--tRNA ligase	*Eprs*	45	44	0	1	6	4	5	7	7	12	5	4	29.4	170
Q99K30;Q99K30-2	Epidermal growth factor receptor kinase substrate 8-like protein 2	*Eps8l2*	15	15	1	0	3	3	2	4	5	4	3	4	21.9	82

P; Peptides, UP; Unique peptides, SC; Sequence coverage, MW: Molecular weight

Q91WL0	Epidermal growth factor receptor kinase substrate 8-like protein 3	*Eps8l3*	10	10	3	2	2	4	2	4	6	6	5	5	24	68
Q9D1Q6	Endoplasmic reticulum resident protein 44	*Erp44*	11	11	0	2	2	3	4	3	3	2	3	3	25.6	47
H3BJP2;H3BLJ9;H3BJL6;Q9R0P3;H3BKH6;H3BK43;H3BJC6;H3BL99	S-formylglutathione hydrolase	*Esd*	8	8	1	1	3	3	5	5	7	5	5	5	36.4	27
Q8BWY3	Eukaryotic peptide chain release factor subunit 1	*Etf1*	11	11	0	2	1	0	3	4	4	6	5	4	29.7	49
Q99LC5	Electron transfer flavoprotein subunit alpha, mitochondrial	*Etfa*	15	15	3	3	4	6	5	9	8	8	6	6	48.9	35
Q9DCW4	Electron transfer flavoprotein subunit beta	*Etfb*	17	17	7	6	9	8	8	8	9	8	8	9	56.9	28
Q9DCM0	Protein ETHE1, mitochondrial	*Ethe1*	15	15	3	4	5	5	5	9	10	9	9	9	70.1	28
P26040	Ezrin	*Ezr*	68	56	12	10	14	15	12	18	16	24	19	17	65.4	69
P04117	Fatty acid-binding protein, adipocyte	*Fabp4*	7	7	1	0	2	3	3	3	2	2	2	3	33.3	15
Q3UW53;E9PYV4;D3Z233;D3YYZ9	Protein Niban	*Fam129a*	9	9	0	0	2	1	2	1	3	3	4	2	11.3	103
P97805;I7HPY1;I7HLV5;Q9D8T0;I7HJS7	Protein FAM3D	*Fam3d*	5	5	1	1	1	1	1	3	4	3	3	3	18.4	25
P19096	Fatty acid synthase;[Acyl-carrier-protein] S-acetyltransferase;[Acyl-carrier-protein] S-malonyltransferase;3-oxoacyl-[acyl-carrier-protein] synthase;3-oxoacyl-[acyl-carrier-protein] reductase;3-hydroxyacyl-[acyl-carrier-protein] dehydratase;Enoyl-[acyl-carrier-protein] reductase;Oleoyl-[acyl-carrier-protein] hydrolase	*Fasn*	79	79	0	3	9	4	8	22	22	35	33	22	31	272
P70695;Q9QXD6;E9Q0T7;F6YS63	Fructose-1,6-bisphosphatase isozyme 2	*Fbp2*	15	15	2	6	4	6	5	5	6	8	6	6	34.5	37
NP_001116075 GI:169790797;E9Q9C6;E9Q0B5;ENSP00000221347 Human FCGBP;ENSMUSP00000096234 AAI26887 GI:119850803 mouse Fcgbp	Protein Fcgbp	*Fcgbp*	80	68	19	20	24	36	41	26	39	30	28	25	31.2	275
Q920E5	Farnesyl pyrophosphate synthase	*Fdps*	17	17	0	1	1	2	4	5	3	9	8	6	34.3	41
Q8CIB5;A6X941;A6X940	Fermitin family homolog 2	*Fermt2*	12	11	0	0	1	6	4	3	3	5	4	4	16.5	78
Q99K47;E9PV24;CON__P02672	Fibrinogen alpha chain	*Fga*	21	21	2	0	2	7	2	1	4	4	2	3	32.5	61
Q8VCM7	Fibrinogen gamma chain	*Fgg*	15	15	0	0	1	5	0	2	6	6	3	4	32.6	49
P97807-2;P97807;H3BKG7	Fumarate hydratase, mitochondrial	*Fh*	21	21	4	5	8	7	10	9	9	8	8	7	43.9	50
A2AEX8;A2AEX6;P97447;A2AEX7;A2AEY1;A2AEY2;P97447-2;A2AEY0;A2AEX9;Q8CDC8	Four and a half LIM domains protein 1	*Fhl1*	17	17	6	5	9	12	13	6	12	6	7	5	51	34
P26883;F6X9I3	Peptidyl-prolyl cis-trans isomerase FKBP1A	*Fkbp1a*	5	5	2	1	2	3	2	3	2	3	4	4	49.1	12
P30416;F6S2D5;D6RDE2;F7CAT1	Peptidyl-prolyl cis-trans isomerase FKBP4;Peptidyl-prolyl cis-trans isomerase FKBP4, N-terminally processed;Peptidyl-prolyl cis-trans isomerase	*Fkbp4*	36	36	1	5	4	4	12	7	7	13	12	10	65.5	52

P; Peptides, UP; Unique peptides, SC; Sequence coverage, MW: Molecular weight

B7FAU9;Q8BTM8;B7FAV1;F6XC15;J3JS91;Q8BTM8-2;F6Z2C0;F7AVL7	Filamin-A	*Flna*	140	126	34	41	64	68	74	65	81	70	69	75	57.3	281
Q80X90	Filamin-B	*Flnb*	137	121	21	17	32	28	36	41	53	46	40	39	54.7	278
Q8VHX6-2;D3YW87;Q8VHX6;D3Z576	Filamin-C	*Flnc*	40	23	1	4	7	3	6	4	8	6	5	8	15.2	287
P09528	Ferritin heavy chain	*Fth1*	10	10	2	4	4	7	5	7	7	7	6	6	42.9	21
Q9CPX4;P29391;P49945;H3BKD3	Ferritin;Ferritin light chain 1;Ferritin light chain 2	*Ftl1;Ftl2*	14	14	2	2	3	3	1	6	10	5	7	8	66.7	21
G3UXT7;Q8CFQ9;P56959;G3UZD2;Q91VQ2	RNA-binding protein FUS	*Fus*	5	3	0	0	1	0	1	1	0	0	0	0	54.6	14
Q00612;A3KG36;P97324;G3UWD6;REV__P46662	Glucose-6-phosphate 1-dehydrogenase X;Glucose-6-phosphate 1-dehydrogenase	*G6pdx*	28	28	2	2	5	7	5	6	5	7	7	7	50.5	59
Q8R059;B1AV53;B1AV54	UDP-glucose 4-epimerase	*Gale*	18	18	3	3	6	4	3	9	8	9	12	9	41.8	38
Q68FH4;Q8BUU7;B7ZCT4;D6RJ57	N-acetylgalactosamine kinase	*Galk2*	8	8	0	1	0	2	2	0	2	3	4	3	15.5	51
Q8K157	Aldose 1-epimerase	*Galm*	5	5	0	0	0	0	0	3	3	3	4	3	19	38
Q8BHN3;Q8BHN3-2;Q8BHN3-3	Neutral alpha-glucosidase AB	*Ganab*	17	17	0	3	4	3	3	9	3	6	4	4	18.9	107
P16858;F8WJL5;D3YU05;D3YYI5;D3Z0Z9;F6UT49;F6QXU4;Q64467;Q8BJ40	Glyceraldehyde-3-phosphate dehydrogenase	*Gapdh;Gm6316;Gm7293*	32	32	12	12	12	17	14	16	17	15	17	14	70.3	36
Q9CZD3	Glycine--tRNA ligase	*Gars*	21	21	2	1	4	2	5	6	6	7	8	9	27.2	82
P21614	Vitamin D-binding protein	*Gc*	18	18	4	2	6	8	10	3	5	5	4	4	35.7	54
H3BJA3;O09172;F6VNW5	Glutamate--cysteine ligase regulatory subunit	*Gclm*	5	5	0	1	2	1	2	1	3	3	3	3	31.2	22
Q9R111;D3YU09	Guanine deaminase	*Gda*	10	10	0	0	2	1	1	3	3	3	2	1	23.1	51
P50396;B7FAU8	Rab GDP dissociation inhibitor alpha	*Gdi1*	22	16	0	0	1	3	4	4	7	8	7	8	56.8	51
Q61598;Q61598-2	Rab GDP dissociation inhibitor beta	*Gdi2*	36	30	6	4	8	6	10	11	13	13	16	13	72.8	51
P47856-2;P47856;D3YYD9;Q9Z2Z9;D3YYE0	Glutamine--fructose-6-phosphate aminotransferase [isomerizing] 1	*Gfpt1*	47	47	7	10	20	15	25	25	26	26	25	22	57.7	77
Q9Z0L8-2;Q9Z0L8	Gamma-glutamyl hydrolase	*Ggh*	9	9	3	4	3	4	2	6	5	6	6	6	33	35
Q9Z2H7	PDZ domain-containing protein GIPC2	*Gipc2*	10	10	4	1	1	2	1	3	2	4	4	4	28.7	34
P23780	Beta-galactosidase	*Glb1*	6	6	0	0	0	0	0	3	2	3	2	2	11.7	73
Q9CPU0	Lactoylglutathione lyase	*Glo1*	9	9	2	1	3	3	4	3	5	4	5	3	61.4	21
Q9CPV4;Q9CPV4-3;E9Q197;Q9CPV4-2;F6ZTG3;E9Q055;E9Q2R6;F7BB55	Glyoxalase domain-containing protein 4	*Glod4*	16	16	2	2	4	6	8	6	7	6	6	5	49	33
P26443;F7CFA5	Glutamate dehydrogenase 1, mitochondrial	*Glud1*	23	23	2	5	6	4	3	10	7	9	7	7	35.1	61
P15105;D3YVK1;D3Z121	Glutamine synthetase	*Glul*	10	10	1	1	1	1	5	2	1	2	4	4	24.4	42
G3X9L6;Q9DCX2;B1ASE2;D3Z1B2	ATP synthase subunit d, mitochondrial	*Gm10250;Atp5h*	14	14	4	3	3	4	3	6	7	6	6	5	70.6	19
Q1RME4	CAR-like soluble protein	*Gm1123*	11	11	5	3	2	5	0	6	9	6	7	5	29.7	43
F6QL70;P47915;D3Z1N9;F6YH22;F6VZG1;D3Z102	60S ribosomal protein L29	*Gm17669;Rpl29;Gm10709;Gm5218*	9	9	2	2	2	2	2	2	2	1	2	2	41.6	17
E9PZF0;Q01768	Nucleoside diphosphate kinase;Nucleoside diphosphate kinase B	*Gm20390;Nme2*	17	6	2	2	2	2	2	2	3	3	2	2	67	30

P; Peptides, UP; Unique peptides, SC; Sequence coverage, MW: Molecular weight

D3Z3R1;Q6ZWZ4;D3YW41;D3Z7M8;P47964;D3YX71	60S ribosomal protein L36	*Gm5745;Rpl36;Rpl36-ps3*	10	10	1	0	3	4	3	3	1	3	2	2	41.3	12
B2RXM2;Q8BG05-2;J3QNY1;E9Q7H5;A2AL13	0	*Gm6793;Gm9242;Gm8991*	26	3	0	1	1	0	0	1	1	0	1	0	46.4	37
Q8K0C9;J3QMC6	GDP-mannose 4,6 dehydratase	*Gmds*	31	31	10	11	11	11	13	9	14	12	14	10	62.4	42
Q8BTZ7	Mannose-1-phosphate guanyltransferase beta	*Gmppb*	9	9	0	0	3	4	2	2	3	6	4	1	25	40
P21278	Guanine nucleotide-binding protein subunit alpha-11	*Gna11*	14	8	0	2	2	0	0	3	2	3	0	2	43.7	42
P08752	Guanine nucleotide-binding protein G(i) subunit alpha-2	*Gnai2*	9	6	0	0	0	0	0	3	0	2	0	1	26.8	40
P62874;H3BKR2;H3BLF7	Guanine nucleotide-binding protein G(I)/G(S)/G(T) subunit beta-1	*Gnb1*	10	5	2	1	0	1	0	1	3	0	1	1	27.9	37
P68040	Guanine nucleotide-binding protein subunit beta-2-like 1	*Gnb2l1*	17	17	2	3	6	6	8	7	9	9	8	7	50.2	35
Q3UW64;Q91WG8;G3UX23;G3UYD5;G3UZ98;F6WD11;G3UWV5;F6SHH4;F7BWW0;G3UX49	Bifunctional UDP-N-acetylglucosamine 2-epimerase/N-acetylmannosamine kinase;UDP-N-acetylglucosamine 2-epimerase (hydrolyzing);N-acetylmannosamine kinase	*Gne*	31	31	0	1	4	4	8	5	11	13	14	12	39.2	83
Q9DAS9	Guanine nucleotide-binding protein G(I)/G(S)/G(O) subunit gamma-12	*Gng12*	4	4	3	1	2	3	2	2	3	2	1	2	56.9	8
O88958;D3Z0R5	Glucosamine-6-phosphate isomerase 1	*Gnpda1*	8	6	0	0	1	1	2	2	2	3	2	4	28.7	33
Q91XA2	Golgi membrane protein 1	*Golm1*	22	22	4	4	4	4	5	8	3	5	4	5	43.5	44
Q8R088-2;K3W4N3;H3BKI7;Q8R088;H3BJ07;H3BJ82;Q8R088-3;H3BJ12;K3W4Q3;H3BJI3;H3BJN5	Golgi phosphoprotein 3-like	*Golph3l*	9	9	0	0	1	1	2	2	3	4	3	2	40.8	23
P05201;F7ALS6	Aspartate aminotransferase, cytoplasmic	*Got1*	25	25	3	6	5	6	5	10	13	10	10	12	57.4	46
P05202	Aspartate aminotransferase, mitochondrial	*Got2*	20	20	4	6	3	4	6	8	9	7	6	8	37.7	47
Q9JKA5;E9PY15	Cell surface A33 antigen	*Gpa33*	11	11	1	2	2	0	0	7	6	5	3	3	29.2	36
Q3ULJ0-2;Q3ULJ0;D3Z0L6;Q6NXY9	Glycerol-3-phosphate dehydrogenase 1-like protein	*Gpd1l*	14	13	0	0	1	2	5	4	5	3	3	4	35	35
Q64521;A2AQR0	Glycerol-3-phosphate dehydrogenase, mitochondrial	*Gpd2*	18	18	0	2	3	3	2	4	3	2	0	2	26.5	81
P06745;F6SAC3;CON__Q3ZBD7	Glucose-6-phosphate isomerase	*Gpi;Gm1840*	37	37	4	7	11	13	17	14	16	19	17	14	59.3	63
P11352	Glutathione peroxidase 1	*Gpx1*	11	11	0	4	2	3	3	9	8	8	8	8	67.7	22
Q9JHC0	Glutathione peroxidase 2	*Gpx2*	10	10	3	3	1	4	2	5	9	5	7	5	57.4	22
O70325-2;O70325;Q76LV0;Q91XR9	Phospholipid hydroperoxide glutathione peroxidase, mitochondrial;Glutathione peroxidase;Phospholipid hydroperoxide glutathione peroxidase, nuclear	*Gpx4*	5	5	0	0	0	0	1	3	3	2	3	3	27.1	20

P; Peptides, UP; Unique peptides, SC; Sequence coverage, MW: Molecular weight

Q2KHK6;Q8CB12;Q3TR54;Q99NB5	Gasdermin-C2;Gasdermin-C3;Gasdermin-C4	*Gsdmc2;Gsdmc3;Gsdmc4*	10	10	0	0	1	0	0	6	4	4	1	3	19	54
P13020-2;A6PWS5	Gelsolin	*Gsn*	49	2	1	1	1	1	1	1	1	0	1	1	51.3	81
Q8R050-2;Q8R050;Q149F3	Eukaryotic peptide chain release factor GTP-binding subunit ERF3A;Eukaryotic peptide chain release factor GTP-binding subunit ERF3B	*Gspt1;Gspt2*	13	13	1	3	3	3	5	4	4	3	4	4	18.1	69
P47791-2;P47791	Glutathione reductase, mitochondrial	*Gsr*	16	16	1	3	8	6	7	6	8	6	6	7	41.4	51
P51855;Q3UEE2;H3BKH4;A2AQN7;A2AQN9	Glutathione synthetase	*Gss*	26	26	5	6	5	6	7	7	7	11	10	9	49.2	52
P30115	Glutathione S-transferase A3	*Gsta3*	12	8	0	2	2	1	2	2	1	2	1	1	35.3	25
P24472;F6SC55	Glutathione S-transferase A4	*Gsta4*	10	7	1	2	3	2	4	2	1	2	2	2	27	26
P10649;A2AE89;F6WHQ7;D3YVP8;J3QQ61	Glutathione S-transferase Mu 1	*Gstm1*	23	16	6	6	7	7	5	11	13	12	13	13	73.4	26
P15626;D3YX76;Q8R5I6;F6Y363;A2AE91	Glutathione S-transferase Mu 2	*Gstm2*	20	13	1	0	2	4	5	7	9	10	8	7	73.9	26
P19639;D3YVP9;G5E8M7	Glutathione S-transferase Mu 3	*Gstm3*	16	8	1	2	1	3	3	4	3	5	4	4	61.9	26
O09131;D3Z1Q9;Q8K2Q2	Glutathione S-transferase omega-1	*Gsto1*	29	29	10	10	11	12	14	15	17	15	15	15	59.2	27
P19157;P46425;F6RWR5	Glutathione S-transferase P 1;Glutathione S-transferase P 2	*Gstp1;Gstp2*	14	14	6	8	5	3	5	8	9	10	9	9	63.3	24
Q64471;D3Z3X5;D3Z5W7	Glutathione S-transferase theta-1	*Gstt1*	9	9	0	0	3	4	3	2	4	6	4	2	28.8	27
Q99L20	Glutathione S-transferase theta 3	*Gstt3*	11	6	0	0	1	0	3	1	3	2	1	1	41.9	27
P10922	Histone H1.0	*H1f0*	16	16	5	4	4	5	3	5	4	5	6	6	40.2	21
Q9QZQ8;Q9QZQ8-2;Q8CCK0	Core histone macro-H2A.1	*H2afy*	9	9	0	1	2	1	3	6	3	3	2	4	33.6	40
Q61425	Hydroxyacyl-coenzyme A dehydrogenase, mitochondrial	*Hadh*	13	13	2	5	4	5	6	6	7	4	3	5	37.3	34
Q8BMS1	Trifunctional enzyme subunit alpha, mitochondrial;Long-chain enoyl-CoA hydratase;Long chain 3-hydroxyacyl-CoA dehydrogenase	*Hadha*	34	34	9	4	7	9	7	18	15	11	10	9	43.4	83
Q99JY0;D3YXU1	Trifunctional enzyme subunit beta, mitochondrial;3-ketoacyl-CoA thiolase	*Hadhb*	20	20	4	5	8	5	5	10	11	11	11	10	41.1	51
Q99KB8-2;Q99KB8;G5E8T9;E9Q2H8;E9PYA3;D3YWI0;D3YUX8	Hydroxyacylglutathione hydrolase, mitochondrial	*Hagh*	6	6	0	2	2	3	2	2	5	4	3	4	31.2	29
Q9NYQ2	Hydroxyacid oxidase 2	*Hao2*	15	15	2	1	3	2	5	5	5	5	6	7	35.4	39
Q61035	Histidine--tRNA ligase, cytoplasmic	*Hars*	23	20	0	1	0	1	6	3	4	4	3	2	40.5	57
Q91VB8;P01942;P06467;A7M7S6	Hemoglobin subunit alpha	*Hba-a1;Hba*	11	9	5	6	6	6	5	5	5	5	5	5	71.8	15
A8DUK4;E9Q223;P02088;P02089;CON__Q3SX09	Hemoglobin subunit beta-1	*Hbb-b1*	15	14	9	9	9	10	8	9	9	9	9	9	84.4	16
P51859;E0CXA0;E0CYW7	Hepatoma-derived growth factor	*Hdgf*	12	12	2	2	4	4	5	5	6	6	7	4	50.2	26
Q3UGR5;D3YZI3;Q3UGR5-2;D3YUN7	Haloacid dehalogenase-like hydrolase domain-containing protein 2	*Hdhd2*	5	5	0	0	1	0	2	2	2	2	3	2	23.2	29
Q8VDJ3	Vigilin	*Hdlbp*	55	55	3	6	6	7	14	13	7	16	9	13	37	142
P20060	Beta-hexosaminidase subunit beta	*Hexb*	12	12	1	3	2	1	1	5	6	7	7	3	18.7	61

P; Peptides, UP; Unique peptides, SC; Sequence coverage, MW: Molecular weight

P70349;B0R1E3	Histidine triad nucleotide-binding protein 1	*Hint1*	11	11	1	1	2	4	2	4	7	3	5	3	83.3	14
P43275	Histone H1.1	*Hist1h1a*	16	11	2	2	2	3	4	4	4	3	2	4	43.7	22
P43276	Histone H1.5	*Hist1h1b*	20	15	3	3	4	4	4	5	3	3	3	4	46.2	23
P15864;Q07133;I7HFT9	Histone H1.2	*Hist1h1c*	24	8	2	2	4	3	4	2	2	2	2	2	50.5	21
P43277	Histone H1.3	*Hist1h1d*	22	3	0	0	0	0	0	1	1	1	1	1	47.5	22
P43274	Histone H1.4	*Hist1h1e*	20	6	1	1	1	1	2	2	2	2	2	2	43.8	22
P84228;P68433	Histone H3.2;Histone H3.1	*Hist1h3b;Hist1h3a*	17	3	1	1	1	0	0	0	0	0	0	0	47.8	15
P62806	Histone H4	*Hist1h4a*	20	20	8	6	11	9	11	8	7	8	9	8	65	11
G3UVV4;P17710-3;P17710-4;P17710;P17710-2;D3YYR4;D3Z365;E9Q3Z4;E9Q8S8;Q3TRM8	Hexokinase-1	*Hk1*	23	17	0	1	1	0	4	14	5	3	1	2	23.7	102
P63158;D3YVC6;D3YZ18	High mobility group protein B1	*Hmgb1*	20	16	1	1	3	4	5	4	4	5	4	4	47.4	25
P30681	High mobility group protein B2	*Hmgb2*	22	18	2	4	4	4	6	4	4	4	5	4	60.5	24
Q9JL35	High mobility group nucleosome-binding domain-containing protein 5	*Hmgn5*	17	17	2	4	3	5	13	7	5	8	7	4	49.5	45
P97825	Hematological and neurological expressed 1 protein	*Hn1*	9	9	0	0	0	1	1	4	3	3	4	3	84.4	16
O88569	Heterogeneous nuclear ribonucleoproteins A2/B1	*Hnrnpa2b1*	32	2	0	1	1	2	1	1	0	0	0	1	61.2	37
Q99020;Q80XR6;Q20BD0	Heterogeneous nuclear ribonucleoprotein A/B	*Hnrnpab*	13	13	1	2	2	1	3	3	2	4	4	4	41.4	31
Q9Z204-2;Q9Z204;Q9Z204-4;Q9Z204-3;Q9Z204-5;E9Q786;Q8BTF8	Heterogeneous nuclear ribonucleoproteins C1/C2	*Hnrnpc*	19	19	3	4	4	4	3	7	7	7	8	8	52.3	33
Q60668-3;Q60668;F6ZV59;Q60668-4;G5E8G0;Q60668-2;G3X9W0;E9Q5B6;F6SHF3;F7A465;Q9D3U4	Heterogeneous nuclear ribonucleoprotein D0	*Hnrnpd*	16	14	3	3	4	6	3	4	5	4	4	4	44.4	33
Q9Z2X1;Q9Z2X1-2;J3QMQ5;J3QMT0;J3QM80;J3QP45;J3QNH2;J3QMV8	Heterogeneous nuclear ribonucleoprotein F;Heterogeneous nuclear ribonucleoprotein F, N-terminally processed	*Hnrnpf;Gm6430*	14	12	1	2	2	1	5	4	3	7	3	6	39.8	46
P61979-3;B2M1R6;P61979;P61979-2;D3Z5X4;D3YWG1;H3BKD0;H3BLL4;H3BK96;H3BKI8;Q8BT23;H3BLP7;H3BJ43;H3BJS9	Heterogeneous nuclear ribonucleoprotein K	*Hnrnpk;Gm7964*	25	25	4	6	9	10	9	10	11	10	7	10	46.2	49
Q8R081;G5E924;G3UY38;G3UYY3;G3UY56	Heterogeneous nuclear ribonucleoprotein L	*Hnrnpl*	16	16	2	4	4	1	4	4	1	6	7	6	34.8	64
Q9D0E1-2;Q9D0E1;B8JK33;B8JK32;F6W322;F7C9U3;Q91VC4;G3X924	Heterogeneous nuclear ribonucleoprotein M	*Hnrnpm*	44	44	4	6	15	9	14	14	9	10	8	11	62.5	74
G3XA10;Q8VEK3	Heterogeneous nuclear ribonucleoprotein U	*Hnrnpu*	29	29	4	5	6	6	3	9	7	7	6	8	26.1	87
Q00PI9	Heterogeneous nuclear ribonucleoprotein U-like protein 2	*Hnrnpul2*	13	13	1	1	2	3	3	5	5	5	4	4	18.5	85

P; Peptides, UP; Unique peptides, SC; Sequence coverage, MW: Molecular weight

P62748;A2A7R5;D3Z2Z8	Hippocalcin-like protein 1	*Hpcal1*	6	4	0	0	1	1	1	2	1	1	1	2	31.1	22
P00493	Hypoxanthine-guanine phosphoribosyltransferase	*Hprt1*	11	11	0	0	2	0	5	3	3	5	4	5	48.6	25
Q91X72	Hemopexin	*Hpx*	17	17	3	1	3	6	6	2	7	6	7	7	35	51
P51661	Corticosteroid 11-beta-dehydrogenase isozyme 2	*Hsd11b2*	7	7	0	2	0	2	3	4	3	2	2	2	17.6	42
Q99N15;A2AFQ2;O08756	3-hydroxyacyl-CoA dehydrogenase type-2	*Hsd17b10*	9	9	1	1	3	2	0	5	4	2	3	2	42.9	27
P07901;B7ZC50;B7ZC49;A2A6A2	Heat shock protein HSP 90-alpha	*Hsp90aa1*	53	33	5	7	8	8	13	14	11	21	15	15	47.5	85
P11499;E9Q3D6;E9Q0C3;E9PX27;D3Z1R1	Heat shock protein HSP 90-beta	*Hsp90ab1*	67	45	6	10	13	14	15	14	18	21	19	15	62.4	83
P08113;F7C312	Endoplasmin	*Hsp90b1*	50	48	7	10	18	13	18	22	17	19	18	19	49.1	92
Q61696;P17879	Heat shock 70 kDa protein 1A;Heat shock 70 kDa protein 1B	*Hspa1a;Hspa1b*	31	14	3	2	1	3	4	4	5	3	4	5	45.2	70
Q3U2G2;Q61316	Heat shock 70 kDa protein 4	*Hspa4*	57	53	4	4	9	9	16	16	20	19	21	19	59.7	94
P48722;P48722-2;E0CY23;F6SMW7;F6TFH3	Heat shock 70 kDa protein 4L	*Hspa4l*	20	17	1	0	0	0	3	3	3	7	4	2	25.2	94
P20029	78 kDa glucose-regulated protein	*Hspa5*	62	60	15	13	17	14	23	18	14	22	17	15	59.7	72
P63017;Q504P4;D3Z5E2;D3YW43	Heat shock cognate 71 kDa protein	*Hspa8*	65	45	14	12	15	18	16	18	18	18	16	18	67.5	71
P38647	Stress-70 protein, mitochondrial	*Hspa9*	43	42	5	6	11	7	12	16	16	19	15	12	53	73
P14602-2;P14602;P14602-3;D3YZ06	Heat shock protein beta-1	*Hspb1*	11	11	3	4	5	3	3	2	5	3	5	4	66.5	22
P63038;P63038-2;D3Z2F2;D3Z7J9	60 kDa heat shock protein, mitochondrial	*Hspd1*	48	48	11	12	11	11	15	19	17	15	14	15	60.4	61
Q64433	10 kDa heat shock protein, mitochondrial	*Hspe1*	17	17	6	6	6	6	6	6	6	7	6	5	96.1	11
Q9JKR6;E0CYZ2;F6TRP3	Hypoxia up-regulated protein 1	*Hyou1*	31	31	0	0	1	1	3	8	3	3	5	3	32.6	111
O88844;D3YVY3	Isocitrate dehydrogenase [NADP] cytoplasmic	*Idh1*	35	33	6	8	7	10	13	12	13	13	11	14	66.2	47
P54071;D6RIL6	Isocitrate dehydrogenase [NADP], mitochondrial	*Idh2*	25	23	0	1	3	4	7	7	7	5	6	8	48.7	51
Q9D6R2;Q9D6R2-2	Isocitrate dehydrogenase [NAD] subunit alpha, mitochondrial	*Idh3a*	17	17	6	4	5	4	8	9	9	9	6	8	39.1	40
Q91VA7	Isocitrate dehydrogenase 3 (NAD+) beta	*Idh3b*	19	19	3	3	5	8	5	12	11	11	7	7	47.1	42
P70404	Isocitrate dehydrogenase [NAD] subunit gamma 1, mitochondrial	*Idh3g*	11	11	0	0	4	4	3	3	4	3	5	5	31	43
P25085-2;A2AIU1;Q3TBV5;P25085	Interleukin-1 receptor antagonist protein	*Il1rn*	5	5	1	0	1	1	1	2	4	3	3	2	33.3	18
Q8CAQ8-3;Q3TZK4;E9PVS5	MICOS complex subunit Mic60	*Immt*	44	1	1	1	1	0	0	1	1	1	1	1	53	79
Q924B0;O55023;Q80ZJ2;D3Z703	Inositol monophosphatase 1	*Impa1*	16	16	2	3	4	4	9	5	7	7	7	7	49.5	30
Q9JKF1;F8VQ29	Ras GTPase-activating-like protein IQGAP1	*Iqgap1*	83	80	4	9	16	18	18	39	43	44	41	39	51.3	189
Q3UQ44	Ras GTPase-activating-like protein IQGAP2	*Iqgap2*	66	63	1	0	4	0	3	24	18	25	20	16	38.2	181
Q9CX00	IST1 homolog	*Ist1*	8	8	1	2	2	1	4	3	3	3	3	2	15.7	39
Q9D892	Inosine triphosphate pyrophosphatase	*Itpa*	6	6	1	1	2	1	1	2	3	3	4	3	30.8	22
Q9JHI5	Isovaleryl-CoA dehydrogenase, mitochondrial	*Ivd*	10	10	0	2	3	1	2	6	4	4	1	2	20.8	46
Q02257	Junction plakoglobin	*Jup*	28	25	0	0	1	1	3	12	6	10	3	3	40.7	82
Q99MN1;Q8R2P8	Lysine--tRNA ligase	*Kars*	30	30	1	2	7	8	11	9	9	13	11	11	36.5	68
Q3U0V1	Far upstream element-binding protein 2	*Khsrp*	18	15	1	2	4	2	4	9	6	7	6	7	26.9	77
E9Q4K7	Protein Kif13b	*Kif13b*	38	36	1	1	4	2	4	7	13	7	8	8	21.3	205

P; Peptides, UP; Unique peptides, SC; Sequence coverage, MW: Molecular weight

Q61768;E9QAK5;P28738;P33175;A2ARD4	Kinesin-1 heavy chain	*Kif5b*	64	64	5	2	9	10	17	18	17	18	19	15	53.3	110
Q9DBS5;D3Z5Y7;D3Z710;Q91W40	Kinesin light chain 4	*Klc4*	19	17	1	0	4	2	2	4	5	6	4	3	30.9	69
P15947;P15945;P00757;Q61759;P15948;P36369;P36368;P15949;P04071;P00755;P00756;P15946;Q9JM71;Q61754	Kallikrein-1	*Klk1*	16	16	7	7	7	10	7	7	13	12	12	8	52.1	29
O08677-2;D3Z2B2;Q6S9I0;Q6S9I2;Q6S9I3;CON__P01045-1;CON__Q2KJ62;CON__P01044-1	Kininogen-1;Kininogen-2	*Kng1;Kng2*	18	2	1	1	1	2	1	1	1	1	1	1	42.8	48
P70168	Importin subunit beta-1	*Kpnb1*	14	14	0	0	1	0	1	3	2	6	5	1	17.8	97
P11679;CON__H-INV:HIT000292931;CON__Q9H552;CON__H-INV:HIT000016045;CON__REFSEQ:XP_092267	Keratin, type II cytoskeletal 8	*Krt8*	78	38	13	12	13	14	16	15	16	20	14	15	76.7	55
P57016	Ladinin-1	*Lad1*	28	28	5	6	7	4	4	6	10	9	8	6	44.3	59
Q9CPY7-2;Q9CPY7	Cytosol aminopeptidase	*Lap3*	24	24	1	1	5	3	8	6	7	9	9	8	43.2	53
Q61792;A2A6H0;A2A6G9;A2A6G7;A2A6G8;A2A6G6;A2A6H1;A2A6G5;E9Q0N6;A2A6G0;A2A6G4;Q9DC07	LIM and SH3 domain protein 1	*Lasp1*	16	16	6	4	5	5	8	9	8	9	9	8	49.8	30
Q61233;D3YZ25;D3YVW8;D3Z7D9;D3Z311	Plastin-2	*Lcp1*	25	23	1	1	3	4	12	12	7	11	13	11	41	70
P06151;G5E8N5;D3Z736;D3YZQ9;D3YVR7;D3YZE4;P00342	L-lactate dehydrogenase A chain;L-lactate dehydrogenase	*Ldha*	31	29	3	4	11	10	13	10	10	12	11	11	56	36
P16125;D3Z7F0	L-lactate dehydrogenase B chain;L-lactate dehydrogenase	*Ldhb*	9	7	0	0	3	0	2	2	6	5	4	3	31.7	37
P16045	Galectin-1	*Lgals1*	7	7	2	0	2	5	4	3	4	2	3	4	51.9	15
Q8C253;P16110	Galectin-3	*Lgals3*	15	15	6	6	8	7	8	8	7	8	7	6	36	27
Q8K419;O54891	Galectin-4	*Lgals4*	19	19	7	7	12	11	11	12	12	11	10	12	54.9	36
Q9ERG0;Q9ERG0-2;Q8BGB5	LIM domain and actin-binding protein 1	*Lima1*	44	44	12	6	12	9	13	12	16	13	12	10	49.9	84
O88952;Q8JZS0;Q3TUM0;O88951	Protein lin-7 homolog C	*Lin7c*	7	7	1	0	1	3	1	3	2	4	3	3	43.7	22
P48678;P48678-2;P48678-3;D3YUF7	Prelamin-A/C;Lamin-A/C	*Lmna*	78	77	22	27	34	31	31	38	32	36	32	34	77.7	74
P14733	Lamin-B1	*Lmnb1*	40	33	2	6	9	9	7	15	8	15	3	8	53.7	67
P21619;P21619-2	Lamin-B2	*Lmnb2*	39	32	1	0	7	3	4	7	7	4	5	5	54	67
Q8BVA4;F8VPR1	Leiomodin-1	*Lmod1*	9	9	1	2	2	4	4	0	1	1	2	1	16.5	66
Q8BFW7;Q8BFW7-5;Q8BFW7-4;Q8BFW7-2;Q8BFW7-3	Lipoma-preferred partner homolog	*Lpp*	23	23	3	1	6	6	4	9	10	10	9	8	46.8	66
E9Q3Y4;Q9ESE1-2;Q9ESE1;Q9ESE1-3	Lipopolysaccharide-responsive and beige-like anchor protein	*Lrba*	26	25	0	2	2	0	2	7	5	8	3	9	9.9	317
Q91XL1;CON__Q2KIF2	Leucine-rich HEV glycoprotein	*Lrg1*	7	7	2	1	1	2	2	2	1	2	2	2	15.2	37
Q3UZ39;Q3UZ39-3;G5E8E1;Q3UZ39-2;E9Q9T1	Leucine-rich repeat flightless-interacting protein 1	*Lrrfip1*	15	14	0	0	0	0	1	1	3	2	0	1	24.6	79
P24527	Leukotriene A-4 hydrolase	*Lta4h*	34	34	1	2	6	7	14	15	18	20	20	14	55.5	69
P51885;CON__Q05443	Lumican	*Lum*	8	8	1	4	1	3	5	1	2	3	4	3	20.7	38
P70202	Latexin	*Lxn*	3	3	1	0	0	0	1	3	3	3	3	3	18.5	25

P; Peptides, UP; Unique peptides, SC; Sequence coverage, MW: Molecular weight

P97823;D3Z111;J3QK48;P97823-2;J3QP56;D3YUG4;D3Z269	Acyl-protein thioesterase 1	*Lypla1*	4	4	0	0	1	0	1	3	4	2	2	2	21.7	25
Q64133	Amine oxidase [flavin-containing] A	*Maoa*	19	19	3	5	3	4	3	11	12	9	6	6	34.2	60
P31938	Dual specificity mitogen-activated protein kinase kinase 1	*Map2k1*	18	11	0	2	1	1	4	3	4	3	2	2	33.8	43
P27546-2;P27546;E9QPW8;P27546-3;E9PZ43;F7CK47;P27546-4;E9PWC0;F6XPV7;F6V4Z1;Q78TF3	Microtubule-associated protein 4;Microtubule-associated protein	*Map4*	32	32	7	4	8	7	8	9	10	11	13	8	31.6	117
P63085;F6VEI7;E9PXX5;E9Q3I6;Q6P5G0;Q61532	Mitogen-activated protein kinase 1	*Mapk1*	15	12	1	2	3	3	4	4	6	5	5	3	48.3	41
Q63844;D3Z3G6;D3Z6D8	Mitogen-activated protein kinase 3	*Mapk3*	19	16	1	2	4	3	3	7	8	7	10	7	42.4	43
Q61166;D3YUY6;D3Z6G3;Q6PER3	Microtubule-associated protein RP/EB family member 1	*Mapre1*	13	13	0	1	2	3	4	3	5	4	4	6	51.9	30
P26645	Myristoylated alanine-rich C-kinase substrate	*Marcks*	9	9	1	0	1	0	0	5	4	4	5	4	33.3	30
Q3THS6;Q91X83	S-adenosylmethionine synthase isoform type-2	*Mat2a*	19	19	2	3	6	4	6	6	7	8	9	7	42.8	44
Q99LB6-2;Q99LB6;E0CYU5	Methionine adenosyltransferase 2 subunit beta	*Mat2b*	8	8	0	1	1	2	3	3	5	5	4	4	29.7	36
P14152;B1ATQ3	Malate dehydrogenase, cytoplasmic	*Mdh1*	21	21	5	6	7	8	10	9	9	10	8	9	47.3	37
P08249	Malate dehydrogenase, mitochondrial	*Mdh2*	25	25	6	9	11	12	15	14	17	14	11	11	73.1	36
Q9D967	Magnesium-dependent phosphatase 1	*Mdp1*	5	5	0	1	0	0	1	3	2	3	3	3	33.5	19
Q99KE1;Q8BMF3	NAD-dependent malic enzyme, mitochondrial	*Me2*	12	12	1	1	1	2	2	8	5	4	4	5	20.4	66
Q9DCS3;A2A845	Trans-2-enoyl-CoA reductase, mitochondrial	*Mecr*	14	14	1	0	3	2	5	4	4	4	3	3	36.7	40
P34884	Macrophage migration inhibitory factor	*Mif*	5	5	2	0	3	2	1	3	4	4	4	4	47.8	13
Q9D279	Mitotic interactor and substrate of PLK1	*Misp*	31	31	4	5	5	5	3	11	12	8	5	3	45.5	72
Q9CQX8;Q9D6T9;G3UWB2;J3QPR8	28S ribosomal protein S36, mitochondrial	*Mrps36;Mrps36-ps1*	6	6	0	0	0	0	1	2	2	3	2	1	70.6	11
P26041	Moesin	*Msn*	49	37	5	8	10	12	13	12	11	11	16	12	53.4	68
Q7JCZ1;P00405	Cytochrome c oxidase subunit 2	*mt-Co2;Mtco2*	5	5	1	2	3	3	0	4	2	1	2	2	23.8	26
Q922D8	C-1-tetrahydrofolate synthase, cytoplasmic;Methylenetetrahydrofolate dehydrogenase;Methenyltetrahydrofolate cyclohydrolase;Formyltetrahydrofolate synthetase	*Mthfd1*	39	39	2	2	9	7	12	10	12	13	14	10	39.5	101
P62774	Myotrophin	*Mtpn*	11	11	0	0	1	3	4	2	4	4	3	2	74.6	13
P19467;mMuc13_protein_LT200504	Mucin-13	*Muc13*	12	12	4	4	2	5	3	4	5	5	5	3	17.5	59
Q80Z19;mMuc2_protein_LT200504;Q9R1L0;F6QGV1;hMUC2_protein_LT200503;pSN_MG_protein_MJ080907;pSMG_C_protein_MJ080907	Mucin-2	*Muc2*	88	88	21	22	31	40	41	33	57	41	35	29	31.5	293
P28665;P28666;CON__ENSEMBL:ENSBTAP00000024146	Murinoglobulin-1;Murinoglobulin-2	*Mug1;Mug2*	24	24	4	7	4	8	4	3	3	8	3	5	18.2	165

P; Peptides, UP; Unique peptides, SC; Sequence coverage, MW: Molecular weight

E9Q3X0;D3Z2N7	Major vault protein	*Mvp*	67	1	0	0	0	0	0	1	1	1	1	1	64.6	97
Q9JK81;F8WGG3;F7A3N3	UPF0160 protein MYG1, mitochondrial	*Myg1*	8	8	0	0	0	0	1	3	4	3	6	2	25	43
E9QPE7;O08638-2;Q02566	Myosin-11;Myosin-6	*Myh11;Myh 6*	164	9	2	1	4	4	4	3	3	5	4	5	65.8	223
Q6URW6;Q6URW6-2;K3W4R2;Q6URW6-3;D3Z5V8	Myosin-14	*Myh14*	179	167	29	22	36	27	36	62	70	64	59	53	64.8	229
Q8VDD5;F2Z494;Q8BXF2;Q9D6A1;E9PVB9;E9PWM9;B8JJH5;F6VXK7;E9Q264;Q91Z83;P13542;B1AR69;Q5SX39;P13541;A2AQP0;Q5SX40;G3UW82;Q5MJ56;P97479-2;P97479;Q9QZZ4-3;Q9QZZ4-2;Q9QZZ4	Myosin-9	*Myh9*	217	185	28	30	47	41	57	68	72	74	70	68	72.6	226
Q60605-2;Q60605	Myosin light polypeptide 6	*Myl6*	15	13	4	6	6	7	7	7	7	7	6	6	70.2	17
Q9CQ19	Myosin regulatory light polypeptide 9	*Myl9*	12	5	0	1	2	2	2	2	3	4	2	4	65.7	20
Q6PDN3-3;Q6PDN3;B1B1A8;Q6PDN3-2	Myosin light chain kinase, smooth muscle;Myosin light chain kinase, smooth muscle, deglutamylated form	*Mylk*	43	35	8	12	16	17	21	14	20	17	16	14	39.7	114
I7HPW8;F6Y6G7;F6YB52	Protein Myo15b	*Myo15b*	32	15	2	1	4	3	2	6	6	7	2	3	16.6	259
O88329	Unconventional myosin-Ia	*Myo1a*	41	38	7	8	8	6	5	21	24	16	16	18	36.8	119
Q5SYD0;Q5SYD0-2;Q5SUA5	Unconventional myosin-Id	*Myo1d*	40	39	2	1	5	0	0	17	13	15	12	14	40.2	116
E9PVU0;E9Q175;E9Q174;E9Q3L1;Q64331	Unconventional myosin-VI	*Myo6*	53	53	1	5	6	7	7	15	14	16	13	10	38.7	146
Q99MZ6	Unconventional myosin-VIIb	*Myo7b*	23	22	3	1	2	0	0	6	4	6	3	4	10.6	241
P01837	Ig kappa chain C region	*N/A*	6	6	1	1	0	1	3	3	2	0	2	3	49.1	12
P01878	Ig alpha chain C region	*N/A*	7	7	1	3	1	2	0	3	2	2	2	3	21.8	37
Q91V76	Ester hydrolase C11orf54 homolog	*N/A*	14	14	2	0	2	5	4	2	6	4	2	3	40	35
Q60817;P70670	Nascent polypeptide-associated complex subunit alpha;Nascent polypeptide-associated complex subunit alpha, muscle-specific form	*Naca*	6	6	2	1	2	1	3	4	4	4	3	3	29.8	23
Q9QWR8	Alpha-N-acetylgalactosaminidase	*Naga*	5	5	0	0	1	0	2	3	3	2	2	3	13	47
Q99KQ4	Nicotinamide phosphoribosyltransferase	*Nampt*	16	16	0	0	1	0	5	1	3	4	6	2	37.7	55
Q99J77	N-acetylneuraminic acid synthase	*Nans*	29	29	4	8	8	12	11	10	13	13	11	11	58.5	40
Q78ZA7	Nucleosome assembly protein 1-like 4	*Nap1l4*	9	7	0	1	1	0	2	2	2	3	2	2	31.7	43
B1AU76;Q99MD9;B1AU75;Q99MD9-2	Nuclear autoantigenic sperm protein	*Nasp*	11	11	0	0	2	1	3	2	2	2	5	4	24.1	49
P28660;A2AS98;P28660-2;Q8K1X4	Nck-associated protein 1	*Nckap1*	17	17	0	0	1	0	2	6	6	6	4	5	15.9	129
P09405	Nucleolin	*Ncl*	34	34	4	7	14	9	18	10	11	11	9	14	42.6	77
Q62425	NADH dehydrogenase [ubiquinone] 1 alpha subcomplex subunit 4	*Ndufa4*	6	6	2	0	1	1	1	3	3	5	3	3	56.1	9
Q9Z1P6	NADH dehydrogenase [ubiquinone] 1 alpha subcomplex subunit 7	*Ndufa7*	11	11	3	3	3	3	2	2	1	2	1	1	69.9	13
Q9DCJ5	NADH dehydrogenase [ubiquinone] 1 alpha subcomplex subunit 8	*Ndufa8*	8	8	0	2	2	3	4	2	2	2	1	1	41.3	20
Q91VD9	NADH-ubiquinone oxidoreductase 75 kDa subunit, mitochondrial	*Ndufs1*	29	29	3	6	5	3	6	10	5	3	5	6	37.8	80

P; Peptides, UP; Unique peptides, SC; Sequence coverage, MW: Molecular weight

Q9CXZ1;E9QPX3	NADH dehydrogenase [ubiquinone] iron-sulfur protein 4, mitochondrial	*Ndufs4*	5	5	3	2	3	2	4	2	3	4	3	3	26.3	20
D3YUM1;Q91YT0;D3Z1U9;D3YXX5;D3Z0K1;D6RG60	NADH dehydrogenase [ubiquinone] flavoprotein 1, mitochondrial	*Ndufv1*	11	11	2	2	3	2	3	5	5	3	3	2	25.7	50
Q9JHW2	Omega-amidase NIT2	*Nit2*	7	7	0	1	2	1	2	3	4	4	4	3	27.5	31
Q5NC80;P15532;Q5NC79	Nucleoside diphosphate kinase A	*Nme1*	12	1	1	0	1	1	1	0	0	1	1	1	69.3	14
Q810Q5;A2AK37	Normal mucosa of esophagus-specific gene 1 protein	*Nmes1;AA467197*	10	10	3	3	5	3	5	4	3	3	2	3	59	10
Q99K48;Q99K48-2;B1AXT0	Non-POU domain-containing octamer-binding protein	*Nono*	17	15	0	4	2	3	2	5	4	4	3	4	27.5	55
Q11011;F6QYF8;E9Q039;E9Q6F4;F6V7K3;F7ANF4;F2Z3V5	Puromycin-sensitive aminopeptidase	*Npepps*	25	25	0	0	4	2	4	8	7	9	7	6	25.7	103
Q61937;Q5SQB0;Q9DAY9;Q5SQB5;E9Q5T3	Nucleophosmin	*Npm1;Gm5611*	13	13	2	3	3	3	5	2	1	2	2	3	39.7	33
P46460;G3UX86;G3UX98	Vesicle-fusing ATPase	*Nsf*	19	19	0	0	3	0	1	4	2	6	2	2	28.8	83
Q9CZ44;Q9CZ44-3;Q9CZ44-2;A2AT02	NSFL1 cofactor p47	*Nsfl1c*	22	22	0	2	4	3	3	5	4	7	3	4	68.4	41
Q9JM14;A2A9X5	5(3)-deoxyribonucleotidase, cytosolic type	*Nt5c*	11	11	3	1	2	5	3	3	7	4	4	5	45	23
Q9D020-1;Q9D020	Cytosolic 5-nucleotidase 3	*Nt5c3*	15	15	1	1	1	3	4	7	11	10	11	6	55.9	34
Q02819;H3BK79;D3Z7D7;D3Z1N1	Nucleobindin-1	*Nucb1*	37	37	2	5	3	7	7	12	12	8	9	8	71.5	53
P29758	Ornithine aminotransferase, mitochondrial	*Oat*	16	16	0	0	3	1	1	5	3	4	2	2	36.7	48
Q60597;Q60597-2;Q60597-4;Q60597-3;E9Q7L0;Q5SVY0	2-oxoglutarate dehydrogenase, mitochondrial	*Ogdh*	38	38	3	2	4	3	6	20	17	14	12	8	38	116
Q9CZ30;Q9CZ30-2;B1AYJ9;Q99K73	Obg-like ATPase 1	*Ola1*	15	15	3	1	3	5	6	5	6	9	8	7	36.4	45
Q7TQI3;D3YWF6;D3Z7K0	Ubiquitin thioesterase OTUB1	*Otub1*	9	9	0	1	2	1	0	2	3	3	3	3	38	31
Q9D0K2;Q3UJQ9	Succinyl-CoA:3-ketoacid coenzyme A transferase 1, mitochondrial;Succinyl-CoA:3-ketoacid-coenzyme A transferase	*Oxct1*	15	15	6	4	4	2	2	6	8	3	4	4	29	56
P09103;E9Q8G8	Protein disulfide-isomerase	*P4hb*	51	51	14	12	12	12	19	19	15	23	17	16	69	57
P50580;D3YVH7	Proliferation-associated protein 2G4	*Pa2g4*	26	26	4	6	7	2	9	11	10	11	9	10	57.9	44
P29341;Q9D4E6;Q62029;A2A5N3;F6ZAX1;G5E8X2;Q8C7D3	Polyadenylate-binding protein 1	*Pabpc1*	36	25	4	4	5	5	7	10	6	7	8	9	53.3	71
Q9WVE8	Protein kinase C and casein kinase substrate in neurons protein 2	*Pacsin2*	19	16	2	2	6	4	5	5	6	8	7	6	31.1	56
P63005;P63005-2;Q5SW16	Platelet-activating factor acetylhydrolase IB subunit alpha	*Pafah1b1*	15	15	0	1	0	2	2	5	7	6	6	6	38.8	47
Q61205;D3Z7E6;D3Z2X5;Q8CA83	Platelet-activating factor acetylhydrolase IB subunit gamma	*Pafah1b3*	11	11	2	3	2	1	3	3	2	3	2	2	49.1	26
Q8CIN4;A3KGC3;A3KGC4	Serine/threonine-protein kinase PAK 2;PAK-2p27;PAK-2p34	*Pak2*	21	13	0	1	1	1	4	3	3	5	6	3	42.2	58
Q60967	Bifunctional 3-phosphoadenosine 5-phosphosulfate synthase 1;Sulfate adenylyltransferase;Adenylyl-sulfate kinase	*Papss1*	17	16	0	1	2	2	3	2	2	2	4	3	28.8	71

P; Peptides, UP; Unique peptides, SC; Sequence coverage, MW: Molecular weight

O88428	Bifunctional 3-phosphoadenosine 5-phosphosulfate synthase 2;Sulfate adenylyltransferase;Adenylyl-sulfate kinase	*Papss2*	27	26	4	5	5	3	6	11	11	13	12	12	38.2	70
Q99LX0;A2A813;A2A815;A2A817;A2A816	Protein DJ-1	*Park7*	16	16	5	4	5	8	8	5	5	6	7	5	72.5	20
Q3UF75;Q9EPC1;Q9ES46	Alpha-parvin	*Parva*	7	7	0	1	4	2	3	3	4	3	4	4	26.2	38
Q05920;G5E8R3;E9QPD7	Pyruvate carboxylase, mitochondrial;Pyruvate carboxylase	*Pc;Pcx*	34	34	1	2	8	3	3	12	8	8	6	4	30.3	130
P61458	Pterin-4-alpha-carbinolamine dehydratase	*Pcbd1*	6	5	1	2	0	2	0	2	2	4	4	3	49	12
P60335;P57722-2;P57722;E9Q7D8;G3UYM5;P57724	Poly(rC)-binding protein 1	*Pcbp1*	15	8	3	3	2	4	5	4	5	6	4	6	44.7	37
Q61990-2;Q61990-3;Q61990	Poly(rC)-binding protein 2	*Pcbp2*	11	4	0	1	1	1	2	3	3	3	2	3	38.4	35
P56812;D3Z7Q5	Programmed cell death protein 5	*Pdcd5*	8	8	1	0	0	3	1	3	3	4	4	4	62.7	14
Q9WU78;Q9WU78-3;Q9WU78-2	Programmed cell death 6-interacting protein	*Pdcd6ip*	47	47	4	1	13	8	14	15	18	20	17	16	42.7	96
P35486;P35487	Pyruvate dehydrogenase E1 component subunit alpha, somatic form, mitochondrial	*Pdha1*	24	24	4	4	7	3	4	7	9	7	6	6	41.3	43
Q9D051	Pyruvate dehydrogenase E1 component subunit beta, mitochondrial	*Pdhb*	13	13	0	0	1	1	3	4	4	5	3	5	34.8	39
P27773;F6Q404	Protein disulfide-isomerase A3	*Pdia3*	57	57	16	13	17	19	20	21	18	26	21	22	76.4	57
P08003	Protein disulfide-isomerase A4	*Pdia4*	47	47	2	4	4	5	9	17	9	16	9	13	53.6	72
Q3TML0;Q922R8	Protein disulfide-isomerase A6	*Pdia6*	17	17	1	3	3	3	3	7	5	7	6	6	37.8	49
O70400	PDZ and LIM domain protein 1	*Pdlim1*	19	19	5	5	6	7	10	12	15	13	10	12	72.2	36
O70209	PDZ and LIM domain protein 3	*Pdlim3*	12	12	3	1	3	3	6	3	4	4	4	6	42.1	34
Q99K01-2;Q99K01;Q99K01-4;Q99K01-3;D3YZA7;Q99K01-5	Pyridoxal-dependent decarboxylase domain-containing protein 1	*Pdxdc1*	30	30	0	2	6	3	6	15	11	11	9	10	38.8	87
Q8K183;D3Z7R1	Pyridoxal kinase	*Pdxk*	10	10	3	0	3	1	2	3	6	5	6	5	33.3	35
Q9CZG9	PDZ domain-containing protein 11	*Pdzd11*	3	3	0	1	0	0	1	2	2	2	2	2	37.1	16
P70296;D3Z1V4;D6RHS6	Phosphatidylethanolamine-binding protein 1;Hippocampal cholinergic neurostimulating peptide	*Pebp1*	9	9	3	3	4	4	5	5	5	5	5	5	63.1	21
Q11136;G3UXC5	Xaa-Pro dipeptidase	*Pepd*	13	13	1	1	2	1	4	2	4	6	6	3	25.6	55
Q5SUR0;F6RUL9;G3UY86	Phosphoribosylformylglycinamidine synthase	*Pfas*	11	11	0	0	0	0	1	3	2	3	4	2	11.1	145
P12382	6-phosphofructokinase, liver type	*Pfkl*	22	18	0	0	2	2	3	8	10	10	8	6	31.3	85
Q9WUA3;Q8C605;Q9WUA3-2;F6YL81;D3YUA3	6-phosphofructokinase type C;6-phosphofructokinase	*Pfkp*	25	22	0	1	1	4	4	6	6	6	6	6	26.5	85
Q9DBJ1;O70250	Phosphoglycerate mutase 1	*Pgam1*	23	23	9	8	8	11	10	11	11	11	11	11	66.9	29
Q9DCD0	6-phosphogluconate dehydrogenase, decarboxylating	*Pgd*	31	31	2	2	8	4	7	11	13	15	14	12	52.8	53
P09411;P09041	Phosphoglycerate kinase 1	*Pgk1*	40	40	11	9	14	12	14	15	17	15	15	15	83.5	45
Q9CQ60;D3Z4X1;Q8CBG6;F6X8L5	6-phosphogluconolactonase	*Pgls*	8	8	1	2	2	2	4	4	4	6	5	4	39.7	27
Q7TSV4	Phosphoglucomutase-2	*Pgm2*	26	26	2	3	5	6	6	7	9	7	10	9	39.7	69
Q8BWW3;Q9CYR6	Phosphoacetylglucosamine mutase	*Pgm3*	9	9	0	0	2	1	4	6	4	7	4	6	18.4	55

P; Peptides, UP; Unique peptides, SC; Sequence coverage, MW: Molecular weight

Q8CHP8	Phosphoglycolate phosphatase	*Pgp*	11	11	2	2	2	4	4	4	7	6	6	5	34.3	35
P67778;Q5SQG5	Prohibitin	*Phb*	16	16	4	3	7	6	4	9	6	10	10	8	57	30
O35129;F6QPR1;F6Q8V7	Prohibitin-2	*Phb2*	23	23	4	1	9	3	6	7	10	9	7	7	63.5	33
O70570;D3YVM4;D3Z2D3	Polymeric immunoglobulin receptor;Secretory component	*Pigr*	18	18	2	3	3	1	4	2	5	2	2	1	21.4	85
P53810;J3QQ30;J3QPW1;F8WGG5	Phosphatidylinositol transfer protein alpha isoform	*Pitpna*	15	14	1	2	3	2	5	3	6	6	7	6	46.1	32
E9Q509;G3X925;P53657;D3Z2C4	Pyruvate kinase;Pyruvate kinase isozymes R/L	*Pklr*	23	21	2	2	2	3	1	2	4	4	7	3	37.4	59
P52480-2	Pyruvate kinase PKM	*Pkm*	58	3	2	1	2	2	2	3	3	3	3	3	74.4	58
P52480	Pyruvate kinase isozymes M1/M2	*Pkm*	60	5	2	2	2	2	4	2	2	2	2	2	77	58
P51432	1-phosphatidylinositol 4,5-bisphosphate phosphodiesterase beta-3	*Plcb3*	38	38	1	1	3	5	12	3	7	4	4	6	28.8	139
Q9QXS1-3;E9QMZ5;Q9QXS1-15;Q9QXS1-10;E9Q153;Q9QXS1-9;Q9QXS1-12;E9QKX4;Q9QXS1-11;Q9QXS1-13;E9QLJ5;Q9QXS1-5;E9PY96;Q9QXS1-16;Q9QXS1-4;E9QL05;Q9QXS1-14;E9QQ00;Q9QXS1-6;Q9QXS1-7;Q9QXS1-8;E9QK43;Q9QXS1-2;E9QPE8;Q9QXS1;E9QN87;E9Q3W4;F6R059;E9Q9J6;E9PW24	Plectin	*Plec*	237	234	40	24	43	20	28	98	40	34	26	26	48.8	517
Q9DBG5	Perilipin-3	*Plin3*	14	14	2	2	2	1	3	4	3	4	3	2	33.4	47
Q3V0K9;D3Z6J7	Plastin-1	*Pls1*	30	27	3	5	12	9	10	15	13	16	15	15	38.4	70
Q9Z2M7;Q91W01;O35621	Phosphomannomutase 2	*Pmm2*	19	19	1	2	2	4	7	10	12	10	10	8	65.3	28
Q543K9;P23492;Q9D8C9	Purine nucleoside phosphorylase	*Pnp*	15	15	2	0	2	3	2	11	12	9	11	10	63.3	32
Q9D819	Inorganic pyrophosphatase	*Ppa1*	26	26	7	6	6	8	12	10	12	12	10	10	86.9	33
Q91VM9;D3Z636;Q91VM9-2;G8JL76;D3Z096	Inorganic pyrophosphatase 2, mitochondrial	*Ppa2*	12	12	2	2	3	1	3	5	6	5	4	5	41.5	38
P17742;F8VPN3	Peptidyl-prolyl cis-trans isomerase A;Peptidyl-prolyl cis-trans isomerase	*Ppia;Gm5160*	19	19	5	3	4	6	7	7	8	8	6	6	89	18
P24369	Peptidyl-prolyl cis-trans isomerase B	*Ppib*	18	18	3	2	6	3	7	9	8	9	9	8	55.1	24
Q9CR16	Peptidyl-prolyl cis-trans isomerase D	*Ppid*	13	12	0	1	3	3	6	1	3	3	4	4	31.6	41
P36993-2;P36993-4;P36993;P36993-3;P36993-5;Q99NF7;REV__Q3TEG7;REV__P59279	Protein phosphatase 1B	*Ppm1b*	9	7	0	1	1	1	2	0	2	4	2	2	26	43
P62137	Serine/threonine-protein phosphatase PP1-alpha catalytic subunit	*Ppp1ca*	9	4	0	0	0	0	1	1	1	1	1	2	27	38
Q9DBR7;Q9DBR7-2	Protein phosphatase 1 regulatory subunit 12A	*Ppp1r12a*	20	18	0	2	2	2	3	3	6	4	5	2	19	115
Q60829;Q60829-2	Protein phosphatase 1 regulatory subunit 1B	*Ppp1r1b*	12	12	1	3	4	3	5	4	6	5	7	5	75.3	22
Q3UM45;F6TGJ2	Protein phosphatase 1 regulatory subunit 7	*Ppp1r7*	15	15	1	2	2	4	3	7	4	6	6	4	37.1	41

P; Peptides, UP; Unique peptides, SC; Sequence coverage, MW: Molecular weight

Q76MZ3;G3UWL2;G3UXQ1;H3BJ83	Serine/threonine-protein phosphatase 2A 65 kDa regulatory subunit A alpha isoform	*Ppp2r1a*	19	16	0	0	0	0	3	6	6	8	7	7	32.3	65
Q6P1F6;Q925E7;F6RV17;Q8BG02;G3UZP6;G3UXS9;Q6ZWR4-3;Q6ZWR4;Q6ZWR4-2	Serine/threonine-protein phosphatase 2A 55 kDa regulatory subunit B alpha isoform;Serine/threonine-protein phosphatase 2A 55 kDa regulatory subunit B delta isoform	*Ppp2r2a;Ppp2r2d*	10	10	1	0	2	1	0	1	5	4	4	3	24.6	52
P35700;B1AXW5;B1AXW6;B1AXW4	Peroxiredoxin-1	*Prdx1*	22	19	7	9	9	10	11	10	11	10	10	10	58.8	22
Q61171;D3Z4A4	Peroxiredoxin-2	*Prdx2*	17	16	6	6	8	11	7	9	9	8	9	9	68.2	22
P20108	Thioredoxin-dependent peroxide reductase, mitochondrial	*Prdx3*	9	9	2	3	3	1	2	5	4	3	1	2	35.4	28
P99029-2;P99029;G3UZJ4;H3BJQ7	Peroxiredoxin-5, mitochondrial	*Prdx5*	14	14	6	7	7	6	5	8	8	7	7	7	63.6	17
Q6GT24;D3Z0Y2;O08709;Q8BG37	Peroxiredoxin-6	*Prdx6*	31	31	13	15	15	15	15	16	17	16	15	17	80.4	25
Q9QUR6	Prolyl endopeptidase	*Prep*	15	15	1	0	2	1	1	4	8	7	6	7	24.8	81
Q8K1M3;P12367;H3BK84;P31324	cAMP-dependent protein kinase type II-alpha regulatory subunit	*Prkar2a*	15	15	3	3	3	2	3	4	6	5	5	7	39.6	46
O08795;O08795-2	Glucosidase 2 subunit beta	*Prkcsh*	18	18	1	2	2	2	6	3	3	4	4	4	25	59
Q9Z2Y8;Q80ZV3	Proline synthase co-transcribed bacterial homolog protein	*Prosc*	10	10	2	1	1	1	2	2	4	3	5	2	42.3	30
E9PZ00;Q8BFQ1;K3W4L3;Q61207;J3QPG5	Sulfated glycoprotein 1	*Psap*	7	7	1	2	3	3	6	4	3	4	3	3	13.6	61
Q9R1P4	Proteasome subunit alpha type-1	*Psma1*	23	23	5	3	8	9	10	6	7	8	9	8	60.5	30
P49722	Proteasome subunit alpha type-2	*Psma2*	10	10	0	1	2	1	2	3	2	3	3	4	33.8	26
O70435;F7BWF9;E0CZ34;F8WH02;E0CYL6	Proteasome subunit alpha type-3	*Psma3*	19	19	4	2	4	3	4	10	9	9	8	8	57.3	28
Q9R1P0;E9PW69;E9Q0X0	Proteasome subunit alpha type-4;Proteasome subunit alpha type	*Psma4*	14	14	2	3	4	3	4	4	7	5	4	3	37.5	29
Q9Z2U1;D3YX79	Proteasome subunit alpha type-5;Proteasome subunit alpha type	*Psma5;Gm8394*	12	12	2	4	6	5	6	5	5	5	4	6	50.6	26
Q9QUM9;E0CXB1;E0CYT2	Proteasome subunit alpha type-6	*Psma6*	14	14	2	1	2	3	3	4	7	4	3	3	49.2	27
Q9Z2U0;Q9CWH6	Proteasome subunit alpha type-7	*Psma7*	18	18	4	6	9	7	7	6	5	6	6	6	53.6	28
O09061	Proteasome subunit beta type-1	*Psmb1*	9	9	1	2	3	3	4	4	3	3	4	4	40	26
Q9R1P3	Proteasome subunit beta type-2	*Psmb2*	7	7	0	0	1	0	3	2	2	2	2	2	24.9	23
Q9R1P1;D3YVS3;D3YUM8	Proteasome subunit beta type-3	*Psmb3*	11	11	0	0	1	2	2	2	3	2	2	3	41	23
P99026	Proteasome subunit beta type-4	*Psmb4*	6	6	0	0	0	1	1	3	3	4	3	3	30.7	29
O55234	Proteasome subunit beta type-5	*Psmb5*	8	8	0	3	3	2	1	6	4	7	6	6	34.8	29
Q60692	Proteasome subunit beta type-6	*Psmb6*	6	6	2	2	4	4	3	2	3	3	4	3	25.6	25
P70195	Proteasome subunit beta type-7	*Psmb7*	7	7	1	1	3	3	4	3	2	3	3	2	20.6	30
P62192	26S protease regulatory subunit 4	*Psmc1*	18	18	1	0	1	3	6	2	1	2	3	2	42.3	49
P46471;Q8BVQ9	26S protease regulatory subunit 7	*Psmc2*	23	23	1	1	1	3	3	6	8	13	8	7	45.7	49
O88685;B7ZCF1;A2AGN7;F6Q2E3	26S protease regulatory subunit 6A	*Psmc3*	25	25	0	0	1	1	4	4	5	5	3	4	58.6	50
P62196	26S protease regulatory subunit 8	*Psmc5*	23	23	1	2	2	2	5	7	7	10	6	5	63.3	46
P62334	26S protease regulatory subunit 10B	*Psmc6*	20	20	4	1	4	2	5	5	5	8	7	4	50.1	44
Q8BG32;G3UYI4;G3UYL3;G3UWW7;G3UXL5;G3UYH2;G3UYL8;G3UX15;G3UX67;G3UWV7;G3UZ28;G3UZ33	26S proteasome non-ATPase regulatory subunit 11	*Psmd11*	18	18	1	0	3	5	2	5	3	4	4	4	44.5	47

P; Peptides, UP; Unique peptides, SC; Sequence coverage, MW: Molecular weight

Q9WVJ2;F6ZQQ3;E9Q5I9;F6PXS6;E9Q0U1	26S proteasome non-ATPase regulatory subunit 13	*Psmd13*	10	10	0	0	0	0	1	2	1	3	2	2	24.5	43
Q8VDM4;J3KMQ2;E9Q2S8	26S proteasome non-ATPase regulatory subunit 2	*Psmd2;Gm5422*	18	18	1	0	1	1	3	4	4	12	9	5	22.4	100
P14685;F7B7L8	26S proteasome non-ATPase regulatory subunit 3	*Psmd3*	24	24	0	0	3	0	2	4	4	4	3	6	45.7	61
Q99JI4	26S proteasome non-ATPase regulatory subunit 6	*Psmd6*	14	14	0	1	1	1	3	3	4	6	4	5	38	46
P26516	26S proteasome non-ATPase regulatory subunit 7	*Psmd7*	11	11	0	0	2	2	3	2	2	5	3	2	27.1	37
Q9CR00	26S proteasome non-ATPase regulatory subunit 9	*Psmd9*	10	10	0	2	1	2	2	2	2	4	3	2	51.4	25
G3UXZ5;P97371;G3UXY0;G3X9K9;G3UWN9;G3UXR1	Proteasome activator complex subunit 1	*Psme1*	20	19	5	6	7	6	12	10	11	10	12	11	74.4	27
P97372;G3X9V0;E0CZ90	Proteasome activator complex subunit 2	*Psme2*	13	12	1	0	2	1	2	1	2	4	4	3	42.3	27
Q8BGJ5;Q922I7;Q8CB58;P17225;E9QMW9;F7AXP1;F7DCW4;E9Q279;G3UY95;E9Q0W3;F7C521;B2RU80	Polypyrimidine tract-binding protein 1	*Ptbp1*	15	11	2	1	3	2	4	4	3	3	3	3	28.2	57
D3Z7C6;Q9R0Q7	Prostaglandin E synthase 3	*Ptges3*	7	7	2	1	3	2	5	2	3	2	3	3	42.3	15
Q91YR9;REV__Q3TVC7;REV__Q3TVC7-3	Prostaglandin reductase 1	*Ptgr1*	11	11	0	2	2	5	4	5	6	6	6	4	33.1	36
P26350;J3QPK6	Prothymosin alpha;Thymosin alpha	*Ptma*	10	10	2	3	2	2	4	4	4	4	5	4	42.3	12
Q9D0J8	Parathymosin	*Ptms*	10	10	2	2	2	2	3	2	2	2	2	2	33.7	11
O54724	Polymerase I and transcript release factor	*Ptrf*	9	9	1	5	5	5	4	2	2	5	2	4	24.2	44
Q9EPB4	Apoptosis-associated speck-like protein containing a CARD	*Pycard*	13	13	4	3	3	4	5	6	7	7	6	5	75.1	21
Q9DCC4	Pyrroline-5-carboxylate reductase 3	*Pycrl*	12	12	2	3	6	3	6	4	4	3	4	2	36.9	29
Q8CI94;Q3UEJ6;Q9ET01	Glycogen phosphorylase, brain form	*Pygb*	44	36	0	3	6	5	8	14	15	16	19	15	45.9	97
Q8BML9;D3Z158;Q8R1V9;F6TDS3	Glutaminyl-tRNA synthetase	*Qars*	21	20	0	0	1	1	4	5	3	5	7	3	28.5	88
Q8BVI4;D3YWR7;D3Z1A1;D3Z099	Dihydropteridine reductase	*Qdpr*	13	13	3	4	5	7	5	3	8	5	5	4	58.1	26
Q8BND5-3;Q8BND5-2;Q8BND5;Q8BND5-4	Sulfhydryl oxidase 1	*Qsox1*	11	11	1	2	1	4	2	3	7	6	7	3	21	63
Q5SW88;P62821;Q5SW87;Q5SW86;Q8K386;Q3TYH2	Ras-related protein Rab-1A	*Rab1;Rab1A*	13	8	1	1	0	0	1	3	2	0	2	1	64.9	22
P46638;G3UY29;E9Q3P9;F8WGS1;P62492;G3UZD3;G3UZL4;E9PZB2;E9Q6B3;F6R2Z5	Ras-related protein Rab-11B;Ras-related protein Rab-11A	*Rab11b;Rab11a*	13	13	1	1	3	1	2	7	5	5	3	4	54.1	24
Q91V41;A2AL34	Ras-related protein Rab-14	*Rab14*	12	12	0	1	1	2	2	8	2	3	3	2	61.9	24
P53994;Q3TEG7;P59279;G3UXQ7;G3V022	Ras-related protein Rab-2A;Ras-related protein Rab-2B	*Rab2a;Rab2b*	9	9	0	1	0	0	1	6	3	4	4	3	42.5	24
P35278;Q8C266	Ras-related protein Rab-5C	*Rab5c*	7	5	0	0	0	0	0	3	1	2	1	0	43.1	23
P51150	Ras-related protein Rab-7a	*Rab7a*	9	9	0	1	0	2	2	6	1	2	2	3	52.7	23
P63001;Q3TLP8;A2AC13;P60764;Q05144	Ras-related C3 botulinum toxin substrate 1;Ras-related C3 botulinum toxin substrate 3;Ras-related C3 botulinum toxin substrate 2	*Rac1;Rac3;Rac2*	5	5	0	0	2	1	1	2	3	2	2	1	26.6	21

P; Peptides, UP; Unique peptides, SC; Sequence coverage, MW: Molecular weight

P54728	UV excision repair protein RAD23 homolog B	*Rad23b*	10	7	1	2	0	1	1	4	4	2	4	3	20	44
Q9JIW9;F6QC68	Ras-related protein Ral-B	*Ralb*	8	5	0	1	2	2	2	2	1	1	1	1	31.1	23
P62827;Q14AA6;Q61820	GTP-binding nuclear protein Ran;GTP-binding nuclear protein Ran, testis-specific isoform	*Ran;1700009N14Rik;Rasl2-9*	12	12	2	4	3	6	7	3	4	8	5	5	39.8	24
P34022;H7BX22	Ran-specific GTPase-activating protein	*Ranbp1*	10	9	2	2	3	3	3	3	3	3	3	3	33.5	24
Q9D0I9	Arginine--tRNA ligase, cytoplasmic	*Rars*	18	18	0	0	1	0	2	3	3	6	6	3	24.4	76
A2AFJ1;Q60973;A2AFI9;F6ZLC6;F6U539	Histone-binding protein RBBP7	*Rbbp7*	12	5	0	1	0	0	0	3	0	3	1	1	31	47
O89086;Q8BG13	Putative RNA-binding protein 3	*Rbm3*	3	3	0	1	1	1	0	2	2	2	2	2	25.5	17
Q9CWZ3-2;Q9CWZ3	RNA-binding protein 8A	*Rbm8a*	4	4	0	1	2	2	4	3	3	3	3	2	27.2	20
Q9QUI0	Transforming protein RhoA	*Rhoa*	11	6	1	1	1	1	1	3	2	3	3	2	48.7	22
Q05921	2-5A-dependent ribonuclease	*Rnasel*	15	15	0	0	1	1	0	3	5	6	3	2	22.3	83
Q91VI7	Ribonuclease inhibitor	*Rnh1*	15	15	6	4	5	6	8	7	8	7	9	7	35.3	50
E9PYF1;Q8VCT3	Aminopeptidase B	*Rnpep*	14	14	1	0	0	0	1	3	5	5	3	2	25.5	68
Q6ZWV3;I7HLV2;P86048;B7FAU6	60S ribosomal protein L10;60S ribosomal protein L10-like	*Rpl10;Rpl10l*	16	16	4	4	4	5	5	6	4	4	3	4	42.5	25
Q5XJF6;P53026;D6RE43;D3YXT2	Ribosomal protein;60S ribosomal protein L10a	*Rpl10a*	14	14	0	1	3	2	4	5	6	4	5	5	37.3	25
Q9CXW4;E9PYL9;D3Z3K1;A2BH06;E9PZB3;D3YW69	60S ribosomal protein L11	*Rpl11;Gm10036;Gm10288;Gm5093;Gm7589*	10	10	1	1	1	3	2	3	2	3	2	2	40.4	20
P35979;F8VQK7;D3YX33	60S ribosomal protein L12	*Rpl12*	10	10	1	2	3	4	5	3	5	4	4	3	70.9	18
P47963;D3YX54	60S ribosomal protein L13	*Rpl13;Rpl13-ps3*	22	22	5	7	6	3	5	9	8	8	8	8	56.9	24
P19253;E9Q5A0;D3YY61	60S ribosomal protein L13a	*Rpl13a*	11	11	1	3	3	2	4	5	5	6	6	5	40.9	23
Q9CR57	60S ribosomal protein L14	*Rpl14*	8	8	1	1	3	3	3	3	3	3	3	4	34.1	24
Q9CZM2;E9QAZ2;D3Z0H4;E9Q1X1;B8JKK2	60S ribosomal protein L15;Ribosomal protein L15	*Rpl15;Gm10020;Gm8420*	14	14	3	1	4	3	4	7	7	6	6	5	48.5	24
Q9CPR4;Q6ZWZ7;B2RY53;F6YKV1	60S ribosomal protein L17	*Rpl17*	14	14	0	1	3	4	7	6	3	7	5	4	53.8	21
P35980;G3UZK4;G3UZJ6;G3UX28;G3UYV6	60S ribosomal protein L18	*Rpl18*	14	14	5	3	6	5	4	6	6	6	4	6	48.4	22
P62717;F6YJW4	60S ribosomal protein L18a	*Rpl18a*	10	10	2	2	4	5	4	5	5	6	4	3	39.8	21
A2A547;P84099	60S ribosomal protein L19	*Rpl19*	17	17	1	1	3	5	6	2	2	3	2	2	49	23
Q9CQM8;O09167;D3YX47;D3YX83;D3Z4H7	60S ribosomal protein L21	*Rpl21;Rpl21-ps6*	12	12	0	1	3	2	4	4	4	5	4	3	52.5	19
P67984	60S ribosomal protein L22	*Rpl22*	6	5	2	1	1	0	1	2	2	2	2	2	36.7	15
P62830;A2A6F8	60S ribosomal protein L23	*Rpl23*	8	8	0	1	2	2	3	2	2	1	2	2	47.9	15
P62751;D3YWP3;D3Z1D6;D3YU45;D3YU46;D3YTY6;D3YW68	60S ribosomal protein L23a	*Rpl23a;Rpl23a-ps3;Gm10335;Gm3940*	11	11	3	3	2	2	4	5	4	4	5	5	44.9	18

P; Peptides, UP; Unique peptides, SC; Sequence coverage, MW: Molecular weight

Q8BP67;E9Q132;F6RSK3	60S ribosomal protein L24	*Rpl24*	12	12	2	3	2	4	5	5	6	4	5	4	56.7	18
P61255;B1ARA3;B1ARA5	60S ribosomal protein L26	*Rpl26*	14	4	1	0	2	0	2	1	1	1	1	0	52.4	17
P61358;A2A4Q0;E9PV49	60S ribosomal protein L27	*Rpl27*	9	9	3	2	3	3	2	4	3	5	4	5	55.1	16
P14115	60S ribosomal protein L27a	*Rpl27a*	7	7	0	3	4	2	3	2	3	4	3	2	39.2	17
P41105;F6Z0X0	60S ribosomal protein L28	*Rpl28*	18	18	1	5	3	2	3	6	6	6	5	4	68.6	16
P27659;Q9CQ09;E9PWZ3	60S ribosomal protein L3	*Rpl3*	26	26	3	6	6	7	10	7	8	7	5	5	53.8	46
P62889	60S ribosomal protein L30	*Rpl30*	9	9	2	1	4	4	3	5	5	5	4	5	67	13
P62900;D3YVU2;D3YWP7	60S ribosomal protein L31	*Rpl31*	12	12	2	2	4	3	5	4	2	5	5	4	59.2	14
P62911;A2AD25;P17932;F6WJV0	60S ribosomal protein L32	*Rpl32;Gm4987*	12	12	1	1	2	4	6	2	2	4	1	3	63	16
Q9D1R9;D3YWC0	60S ribosomal protein L34	*Rpl34*	10	10	2	3	3	6	5	4	3	3	3	4	51.3	13
O55142	60S ribosomal protein L35a	*Rpl35a*	13	13	2	1	3	2	3	3	1	4	2	2	60.9	13
Q9JJI8	60S ribosomal protein L38	*Rpl38*	7	7	1	1	1	1	2	3	2	2	2	2	52.9	8
Q9D8E6	60S ribosomal protein L4	*Rpl4*	30	30	4	6	4	4	7	14	12	10	11	12	51.1	47
P47962;D3YYV8	60S ribosomal protein L5	*Rpl5*	19	19	2	2	5	4	3	7	6	7	6	5	42.8	34
P47911;E9PUX4	60S ribosomal protein L6	*Rpl6;Gm5428*	20	20	5	6	7	10	9	11	8	9	7	8	39.2	34
P14148;F6XI62	60S ribosomal protein L7	*Rpl7*	21	21	1	0	4	4	3	9	6	8	7	6	50	31
P12970;D3YXT4;D3YVE6;D3YU93;F6U2H0;F6YI27;F6ZVW4;F6VBB8;F6TIC7;F6SSI5	60S ribosomal protein L7a	*Rpl7a;Gm5619;Rpl7a-ps10;Rpl7a-ps3;Rpl7a-ps5;Gm17415;Gm5459*	19	19	4	2	3	3	7	9	8	8	6	8	53	30
P62918	60S ribosomal protein L8	*Rpl8*	18	18	4	4	6	6	7	4	3	4	3	3	37	28
P14869;E9Q070;D3YVM5	60S acidic ribosomal protein P0	*Rplp0;Gm8730*	8	8	0	0	3	0	4	3	2	6	3	3	30.3	34
P99027	60S acidic ribosomal protein P2	*Rplp2*	6	6	2	2	2	3	3	4	3	4	4	3	60.9	12
Q91YQ5	Dolichyl-diphosphooligosaccharide--protein glycosyltransferase subunit 1	*Rpn1*	22	22	0	2	2	2	3	14	6	2	2	5	33.4	69
P63325;J3QNI7;Q3UW83	40S ribosomal protein S10	*Rps10;Rps10-ps1*	10	10	2	2	4	1	3	2	3	3	2	2	50.3	19
P62281	40S ribosomal protein S11	*Rps11*	17	17	4	4	2	4	3	5	6	5	5	4	74.1	18
Q6ZWZ6;P63323	40S ribosomal protein S12	*Rps12*	6	6	2	1	2	2	2	2	2	2	2	1	40.2	15
P62301	40S ribosomal protein S13	*Rps13*	14	14	1	4	4	3	4	3	3	3	4	3	52.3	17
P62264;D3YVF4;D3Z7I1	40S ribosomal protein S14	*Rps14*	12	12	1	3	3	3	4	5	5	5	5	5	48.3	16
F8WJ41;P62245;D3Z712;D3YVB4	40S ribosomal protein S15a	*Rps15a*	7	7	2	1	2	1	3	3	2	4	2	3	54.6	12
P14131	40S ribosomal protein S16	*Rps16*	18	18	6	4	5	7	8	6	7	6	5	6	77.4	16
P63276	40S ribosomal protein S17	*Rps17*	11	11	1	1	2	1	2	4	2	4	2	1	54.8	16
P62270;F6YVP7;G3UZW2;F5H8M6;D3YWV7	40S ribosomal protein S18	*Rps18;Gm10260;Rps18-ps3*	19	19	2	1	4	3	4	8	6	7	7	8	63.8	18
Q9CZX8;F8VQL3;F6Q5Z8;D3Z722;D3Z5R8;D3YUG3;D3YUT3;F8VQH5	40S ribosomal protein S19	*Rps19*	22	22	4	6	7	5	9	7	6	8	7	7	80	16

P; Peptides, UP; Unique peptides, SC; Sequence coverage, MW: Molecular weight

P25444;D3Z659;D3YVC1;J3QMG5;D3YWJ3;J3QM94;D3Z536;E9Q1N8;F6YTZ4	40S ribosomal protein S2	*Rps2;Rps2-ps6;Gm5786;Gm5070;Gm8225;Gm18025;Gm6576*	13	13	0	1	3	2	5	4	5	4	3	4	33.8	31
P60867	40S ribosomal protein S20	*Rps20*	9	9	2	2	4	2	2	3	2	3	3	3	47.1	13
Q9CQR2	40S ribosomal protein S21	*Rps21*	9	9	1	2	5	4	5	3	1	6	1	2	77.1	9
P62267	40S ribosomal protein S23	*Rps23*	8	8	3	3	3	3	3	2	2	3	3	2	38.5	16
P62849-2;P62849-3;P62849	40S ribosomal protein S24	*Rps24*	8	8	0	0	1	1	0	4	2	3	2	3	46.2	15
P62852;E9Q7H0	40S ribosomal protein S25	*Rps25*	11	11	4	3	2	5	5	3	2	3	3	2	56.8	14
P62855	40S ribosomal protein S26	*Rps26*	5	5	1	2	2	2	3	3	2	1	4	3	44.3	13
Q6ZWU9	40S ribosomal protein S27	*Rps27*	7	4	0	1	1	1	1	1	1	1	1	1	47.6	9
P62858;G3UYV7;J3QNN8	40S ribosomal protein S28	*Rps28*	9	9	1	1	3	2	3	6	3	6	4	6	79.7	8
P62908;D3YV43	40S ribosomal protein S3	*Rps3*	25	25	3	7	8	6	11	11	11	9	9	9	82.3	27
P97351;D3Z6C3;D3Z7W7	40S ribosomal protein S3a	*Rps3a;Rps3a2;Rps3a3*	26	26	5	5	8	8	10	10	9	7	8	10	71.6	30
P62702;Q3V1Z5;D3Z2E6	40S ribosomal protein S4, X isoform	*Rps4x;Rps4y2*	23	23	6	6	8	8	10	8	7	10	5	7	60.1	30
P62754	40S ribosomal protein S6	*Rps6*	23	23	3	4	6	5	8	5	6	6	7	5	46.2	29
E9PWV3;P18653;Q505N6;F6RQA2;F6Q8A4;G3UZI3;Q9WUT3;B1AXN5;B1AXN8;F6UG16;Q7TPS0-2;Q7TPS0-3;Q7TPS0;Q8C0P0-2;E9Q6Q5;Q8C0P0;E9Q6G0;E9Q4Q9;Q811L6-2;E9Q1M8;E9Q8S5;Q3U214;Q9R1L5;B1AST8;Q60592;B1AST7;E9QLW6;E9Q1Q1;E9PWX8;E9QPR4;Q811L6	Ribosomal protein S6 kinase;Ribosomal protein S6 kinase alpha-1	*Rps6ka1*	32	24	0	0	3	0	2	6	6	8	7	7	46	81
P18654;B1AXN9;B1AXP0	Ribosomal protein S6 kinase alpha-3	*Rps6ka3*	17	9	0	0	0	0	1	3	2	6	4	3	25.8	84
P62082;J3QJZ3;F6SVV1;J3QQ42	40S ribosomal protein S7	*Rps7;Gm6472;Gm9493*	19	19	4	3	4	6	6	6	4	4	5	4	52.6	22
P62242;Q5SZV3	40S ribosomal protein S8	*Rps8*	15	15	3	5	8	6	4	7	4	5	6	4	46.6	24
Q6ZWN5;F7CJS8;D3YWH9;Q9CXW7;D3Z673;D3YUV6	40S ribosomal protein S9	*Rps9*	20	20	3	7	8	7	8	8	7	6	4	7	53.1	23
P14206;D3YTT7	40S ribosomal protein SA	*Rpsa;Rpsa-ps10*	14	14	7	6	4	5	5	6	6	6	6	5	42	33
P10833	Ras-related protein R-Ras	*Rras*	6	3	0	1	1	2	2	0	1	1	1	1	27.1	24
A2AVJ7;Q99PL5;Q99PL5-3;Q99PL5-2;Q99PL5-4;Q99PL5-5;Q99PL5-7;Q99PL5-6;Q99PL5-8;Q99PL5-9;Q99PL5-10;Q99PL5-11;Q99PL5-12	Ribosome-binding protein 1	*Rrbp1*	89	88	3	5	18	19	25	19	11	11	10	13	61.5	158
Q9D031;Q01730;A2AUR7;B1AYQ0	Ras suppressor protein 1	*Rsu1*	8	8	0	1	3	3	3	4	3	4	4	4	26.7	31
P60122;D3YW60;Q05CB6	RuvB-like 1	*Ruvbl1*	14	14	1	3	1	1	2	4	2	2	3	5	36	50

P; Peptides, UP; Unique peptides, SC; Sequence coverage, MW: Molecular weight

P97352	Protein S100-A13	*S100a13*	5	5	1	3	2	3	4	3	4	4	3	4	48	11
D3Z2Y6;Q9D708	0	*S100a16*	5	5	1	1	1	3	2	3	3	3	3	3	54.8	8
P14069	Protein S100-A6	*S100a6*	12	12	4	4	3	4	4	3	3	4	3	3	51.7	10
P05366;P05367;P04918	Serum amyloid A-1 protein	*Saa1*	6	6	1	2	0	2	0	3	2	2	2	1	40.2	14
Q9R1T2;Q9R1T2-2	SUMO-activating enzyme subunit 1	*Sae1*	12	12	1	1	2	1	2	1	2	2	3	2	31.1	39
Q60710;F6TVP2;E9Q0K6;F8WJE0;E9PYG9;E0CXZ5	SAM domain and HD domain-containing protein 1	*Samhd1*	20	20	1	2	2	3	9	9	7	6	7	5	30.6	73
P26638;Q8C483;A2AFS0;A2AFS1	Serine--tRNA ligase, cytoplasmic	*Sars*	24	24	3	3	5	6	6	6	4	10	6	6	40.2	58
P70122;F6TN03;D6REV5	Ribosome maturation protein SBDS	*Sbds*	16	16	3	2	5	4	3	1	1	1	4	3	46.8	29
Q60604;Q60604-2	Adseverin	*Scin*	42	42	10	7	11	18	13	22	22	20	25	22	49.7	80
Q8K2B3	Succinate dehydrogenase [ubiquinone] flavoprotein subunit, mitochondrial	*Sdha*	30	30	5	5	9	9	8	11	11	8	7	7	46.8	73
Q9CQA3	Succinate dehydrogenase [ubiquinone] iron-sulfur subunit, mitochondrial	*Sdhb*	18	18	2	3	2	3	2	10	8	7	7	8	46.5	32
Q3UPL0;Q3UPL0-2;Q3UPL0-3	Protein transport protein Sec31A	*Sec31a*	19	19	1	0	2	0	3	4	2	5	5	3	15.4	134
P17563;Q63836;G3UYY2;D6RHN2;G3UWK0	Selenium-binding protein 1;Selenium-binding protein 2	*Selenbp1;Selenbp2*	43	35	14	11	13	14	15	20	21	21	21	19	83.7	53
Q8C1B7-3;Q8C1B7-2;Q8C1B7;A2A3W1	Septin-11	*Sept-11*	8	4	0	0	0	0	1	1	0	0	2	0	17.6	49
O55131;E9Q9F5;E9Q1G8;Q9DA97;E9QMM2	Septin-7	*Sept-7*	15	14	2	1	1	3	1	5	4	5	6	5	29.1	51
P07758;Q00896	Alpha-1-antitrypsin 1-1;Alpha-1-antitrypsin 1-3	*Serpina1a;Serpina1c*	20	7	4	5	5	6	5	2	3	4	4	4	38.5	46
P22599	Alpha-1-antitrypsin 1-2	*Serpina1b*	20	5	3	2	4	4	3	1	3	3	3	4	37.5	46
Q00897	Alpha-1-antitrypsin 1-4	*Serpina1d*	20	7	4	4	5	6	5	2	2	3	3	3	39.7	46
Q00898;D3YZL0	Alpha-1-antitrypsin 1-5	*Serpina1e*	21	8	4	5	5	5	6	5	0	5	5	4	35.4	46
P07759;Q03734;P29621;E9Q499;F2Z405;Q5I2A0;Q80X76;D3Z450	Serine protease inhibitor A3K	*Serpina3k*	16	14	3	6	4	8	5	6	6	7	7	7	35.2	47
Q9D154;Q5SV42;Q8VHP7	Leukocyte elastase inhibitor A	*Serpinb1a*	31	31	9	14	17	13	13	16	17	17	15	16	60.4	43
P70124	Serpin B5	*Serpinb5*	18	18	2	1	4	7	8	12	12	12	11	12	51.7	42
Q60854;F8WIV2;K7E6F1;E9Q3Y1;E9Q6X2;E9Q108;E9Q0P9;E9PYY0;E9PZQ9;E9Q4R2;E9Q5Q5;Q3UWK8;O08804;Q6P6K7	Serpin B6	*Serpinb6;Serpinb6a*	22	22	3	3	7	4	11	11	11	14	10	11	54	43
P32261;CON__P41361	Antithrombin-III	*Serpinc1*	20	20	3	2	5	11	9	4	7	7	4	4	36.1	52
Q9EQU5-2	Protein SET	*Set*	13	1	0	0	0	0	1	0	1	1	1	1	35	32
Q921M3;Q921M3-2	Splicing factor 3B subunit 3	*Sf3b3*	17	17	0	0	1	0	0	4	3	6	2	5	17.4	136
O70456	14-3-3 protein sigma	*Sfn*	26	21	4	3	6	2	6	6	6	7	6	7	68.1	28
Q8VIJ6	Splicing factor, proline- and glutamine-rich	*Sfpq*	26	24	2	4	7	3	3	7	4	3	0	3	29.6	75
Q9JJU8	SH3 domain-binding glutamic acid-rich-like protein	*Sh3bgrl*	5	5	2	0	1	1	2	2	3	3	3	3	35.1	13
Q91VW3;I7HPY0	SH3 domain-binding glutamic acid-rich-like protein 3	*Sh3bgrl3*	7	7	2	2	0	2	1	3	3	3	3	3	55.9	10

P; Peptides, UP; Unique peptides, SC; Sequence coverage, MW: Molecular weight

P50431;G3UZ26;G3UYY1;G3UZZ9	Serine hydroxymethyltransferase, cytosolic;Serine hydroxymethyltransferase	*Shmt1*	14	13	0	0	1	2	3	4	6	6	4	6	31.6	53
Q9WTX5;E9PUV4	S-phase kinase-associated protein 1	*Skp1;Skp1a*	8	8	1	1	2	0	5	2	3	3	2	3	41.7	19
P53986	Monocarboxylate transporter 1	*Slc16a1*	7	7	3	1	2	0	2	5	2	2	2	2	11.8	53
Q8BMD8	Calcium-binding mitochondrial carrier protein SCaMC-1	*Slc25a24*	14	14	2	1	4	2	3	10	6	6	5	4	27.8	53
Q8VEM8;G5E902	Phosphate carrier protein, mitochondrial	*Slc25a3*	10	10	1	3	2	4	2	9	6	4	5	5	26.1	40
P51881;P48962;Q3V132	ADP/ATP translocase 2	*Slc25a5*	22	22	8	7	9	7	8	13	12	9	10	11	53	33
Q9WVC8;E9PY22;E9QAZ3;F7BFQ2	Chloride anion exchanger	*Slc26a3*	18	18	7	6	5	5	3	8	11	10	8	9	21.3	84
Q9JMA9	Sodium- and chloride-dependent neutral and basic amino acid transporter B(0+)	*Slc6a14*	3	3	2	0	1	1	0	3	2	2	2	2	6.9	71
P70441;P70441-2	Na(+)/H(+) exchange regulatory cofactor NHE-RF1	*Slc9a3r1*	30	30	8	4	8	9	6	9	11	11	9	7	71.3	39
Q78PY7;Q3TJ56;E9Q3E9	Staphylococcal nuclease domain-containing protein 1	*Snd1*	34	34	1	1	2	5	5	5	3	10	9	8	37.9	102
Q6NZD2;Q9WV80;D3YWH1	Sorting nexin-1	*Snx1*	17	15	0	0	3	3	3	5	6	6	7	8	37.6	59
Q6ZWQ5;Q3TGS7;Q3V2H3;O70493	Sorting nexin-12	*Snx12*	11	9	2	1	2	3	2	2	1	4	2	2	51.9	19
Q9CWK8	Sorting nexin-2	*Snx2*	28	26	1	1	3	2	6	8	6	8	8	6	48	58
Q78ZM0;D3Z6Z0;O70492;D3Z789	Sorting nexin-3	*Snx3*	9	7	2	2	1	2	4	1	1	2	1	1	53.1	19
Q9D8U8	Sorting nexin-5	*Snx5*	22	21	1	1	3	2	5	5	4	8	8	5	45.8	47
Q6P8X1	Sorting nexin-6	*Snx6*	16	15	0	2	3	2	2	0	2	3	2	4	29.3	47
P08228	Superoxide dismutase [Cu-Zn]	*Sod1*	19	19	7	5	6	5	9	8	8	9	7	8	67.5	16
P09671	Superoxide dismutase [Mn], mitochondrial	*Sod2*	10	10	2	1	3	2	2	5	5	5	3	3	45	25
Q64442	Sorbitol dehydrogenase	*Sord*	13	13	4	4	3	3	5	3	9	5	7	6	38.4	38
P09036	Serine protease inhibitor Kazal-type 3	*Spink3*	5	5	2	2	1	4	3	2	2	2	2	2	27.5	8
Q64105;Q91XH5;G3UXX3;G3UZ79;F6SZQ1	Sepiapterin reductase	*Spr*	13	13	4	5	6	4	6	5	8	5	6	5	59	28
P16546;A3KGU7;P16546-2;A3KGU5;E9Q447;A3KGU9;A3KGU4	Spectrin alpha chain, non-erythrocytic 1	*Sptan1*	148	147	16	22	35	18	27	64	51	54	44	52	56.1	285
Q62261;Q62261-2;Q68FG2	Spectrin beta chain, non-erythrocytic 1	*Sptbn1*	124	120	11	15	23	17	21	48	48	52	33	41	50.1	274
Q9R112;F6ZKZ3;H3BLH2	Sulfide:quinone oxidoreductase, mitochondrial	*Sqrdl*	21	21	0	1	1	0	3	13	5	7	3	3	44.9	50
Q6P069-2;Q6P069;F6T867	Sorcin	*Sri*	13	13	5	2	6	5	7	7	9	7	7	7	52.5	20
Q6PDM2;H7BX95;Q6PDM2-3;Q6PDM2-2;F7AI47;F6QXN3	Serine/arginine-rich splicing factor 1	*Srsf1*	16	16	1	1	6	2	2	3	3	2	3	1	55.6	28
Q62093	Serine/arginine-rich splicing factor 2	*Srsf2*	11	11	2	3	5	3	3	6	3	4	5	2	35.7	25
Q3TWW8	Serine/arginine-rich splicing factor 6	*Srsf6*	8	6	0	0	1	0	0	1	1	1	0	1	17.4	39
Q99L47;F8WJK8;E9Q1V0;E9Q1X9	Hsc70-interacting protein	*St13*	16	16	0	3	5	6	8	4	6	6	5	7	38	42
Q9JMD3;E9PVP0;G3UW37;G3UY87;G3UYM0;G3UY59;G3V020;G3UYN6;G3UZB9	PCTP-like protein	*Stard10*	8	8	0	1	2	3	2	4	6	5	5	4	30.9	33
Q60864	Stress-induced-phosphoprotein 1	*Stip1*	45	45	3	3	10	8	13	12	13	17	18	12	58	63
Q9Z2W1;D3Z359	Serine/threonine-protein kinase 25	*Stk25*	9	6	0	0	1	0	0	1	1	0	0	0	17.6	48

P; Peptides, UP; Unique peptides, SC; Sequence coverage, MW: Molecular weight

P54227;D3Z5N2;D3Z1Z8;Q05DI3;P63042;D3Z4C2;G3X9Z6;O70166;REV__Q9WTN3-4;REV__F6XZS9;REV__Q9WTN3-2;REV__Q9WTN3-3;REV__Q9WTN3	Stathmin	*Stmn1*	17	13	0	0	3	3	3	3	2	3	3	2	73.8	17
Q9Z1Z2	Serine-threonine kinase receptor-associated protein	*Strap*	10	10	0	0	0	2	0	2	1	4	3	3	37.4	38
Q9WUM5	Succinyl-CoA ligase [ADP/GDP-forming] subunit alpha, mitochondrial	*Suclg1*	12	12	4	4	6	4	6	3	5	2	2	2	28.9	36
Q9Z2I8;Q9Z2I8-2	Succinyl-CoA ligase [GDP-forming] subunit beta, mitochondrial	*Suclg2*	30	30	7	5	11	5	14	14	12	13	14	11	60	47
E9QNL5;D3Z3G5	Sulfotransferase	*Sult1a1*	9	0	0	0	0	0	0	0	0	0	0	0	43	34
Q9QWG7;Q9QWG7-2	Sulfotransferase family cytosolic 1B member 1	*Sult1b1*	16	16	3	4	5	4	7	7	10	10	10	9	47.5	35
Q9D939	Sulfotransferase 1C2	*Sult1c2*	8	8	0	0	1	0	0	4	5	4	3	4	24.3	35
Q3UZZ6;Q80VR3	Sulfotransferase 1 family member D1	*Sult1d1*	14	14	2	1	4	1	5	6	8	8	6	6	36.6	35
Q9D2X6	Colon SVA-like protein	*Sval1*	3	3	1	2	3	2	2	2	2	2	2	2	17.2	17
G3UZI2;Q7TMK9-2;G3V018;Q7TMK9;G3UZ48;G3UXJ6;G3UWM1;G3XA76;G3UXU5	Heterogeneous nuclear ribonucleoprotein Q	*Syncrip*	17	10	1	0	0	0	0	4	2	2	3	3	31.5	59
Q70IV5-2;Q70IV5;Q70IV5-3	Synemin	*Synm*	23	23	2	3	6	11	6	0	6	2	4	2	19.5	141
P37804	Transgelin	*Tagln*	28	27	15	15	18	17	17	17	17	19	17	17	90	23
Q9WVA4	Transgelin-2	*Tagln2*	20	17	4	4	4	4	3	11	11	11	11	11	82.4	22
Q93092	Transaldolase	*Taldo1*	28	28	6	8	11	9	11	10	11	12	12	11	56.4	37
Q921F2;Q8R0B4;Q8BLD4;Q6VYI5;Q6VYI4	TAR DNA-binding protein 43	*Tardbp*	8	8	0	0	1	1	2	5	3	5	3	2	20	45
Q9DBG9;B1AUD9	Tax1-binding protein 3	*Tax1bp3*	6	6	0	2	1	2	0	2	2	2	2	3	55.6	14
P48428	Tubulin-specific chaperone A	*Tbca*	10	10	2	2	2	1	3	5	3	5	4	4	68.5	13
Q9D1E6	Tubulin-folding cofactor B	*Tbcb*	8	8	0	2	2	2	1	1	4	4	4	4	33.2	27
P10711;E9PYD5;P10711-2	Transcription elongation factor A protein 1	*Tcea1*	13	13	0	0	1	1	2	3	0	3	4	5	33.9	34
P83940	Transcription elongation factor B polypeptide 1	*Tceb1*	6	6	0	0	0	0	1	2	2	3	3	2	46.4	12
P62869	Transcription elongation factor B polypeptide 2	*Tceb2*	9	9	2	0	3	4	5	3	5	5	6	3	63.6	13
P11983;P11983-2;F2Z483	T-complex protein 1 subunit alpha	*Tcp1*	25	25	0	0	5	5	8	6	7	7	4	5	43	60
Q3ULB1;Q921W7;P47226-2;P47226;D6RH72;A2ALC6;B1AXB9	Testin	*Tes*	19	19	5	5	6	5	10	6	10	8	7	7	43.9	47
Q921I1;E9Q035;F7CJN9;F7BAE9;D3YYR8;E9Q2Q7;E9Q939	Serotransferrin	*Tf;Gm20425*	57	52	15	12	21	25	26	17	29	23	19	20	57.7	77
Q08189;A2ART8	Protein-glutamine gamma-glutamyltransferase E;Protein-glutamine gamma-glutamyltransferase E 50 kDa catalytic chain;Protein-glutamine gamma-glutamyltransferase E 27 kDa non-catalytic chain	*Tgm3*	51	51	15	16	19	25	23	30	32	30	26	27	57.9	77
Q8C1A5	Thimet oligopeptidase	*Thop1*	9	9	0	0	1	3	1	2	3	2	6	1	17.3	78

P; Peptides, UP; Unique peptides, SC; Sequence coverage, MW: Molecular weight

P62075	Mitochondrial import inner membrane translocase subunit Tim13	*Timm13*	3	3	1	0	0	1	1	3	2	2	2	2	37.9	10
Q9Z0U1	Tight junction protein ZO-2	*Tjp2*	20	19	1	1	1	4	5	4	3	4	2	3	18	131
P40142;E0CY51;Q9D4D4	Transketolase	*Tkt*	48	48	11	12	12	16	19	19	18	20	19	19	60.7	68
P26039;A2AIM2;F6SX70;F6S1V7	Talin-1	*Tln1*	101	86	8	13	22	19	32	23	28	31	30	31	42.8	270
Q9JHJ0;Q9JKK7-3;Q9JKK7;P49813	Tropomodulin-3	*Tmod3*	16	16	0	1	1	3	4	5	5	4	4	3	41.8	40
Q61029-3;Q61029;Q61029-2;Q61029-4;Q61033-2;Q61033	Lamina-associated polypeptide 2, isoforms beta/delta/epsilon/gamma;Lamina-associated polypeptide 2, isoforms alpha/zeta	*Tmpo*	15	15	0	1	0	0	1	10	4	2	1	4	37.9	46
Q62393-2;D3Z637;D3Z125;D3Z7X7;Q62393;E9PUA7;Q62393-3;F8WHQ1;D3Z2U2	Tumor protein D52	*Tpd52*	16	16	2	3	3	3	5	6	6	6	5	5	56.8	20
Q3TAI4;Q9CYZ2;A2AUD5;F6VQ81;Q3TUJ9;Q8BKP1	Tumor protein D54	*Tpd52l2*	7	7	1	2	3	1	3	3	4	2	5	2	35.9	22
P17751;H7BXC3	Triosephosphate isomerase	*Tpi1*	26	26	9	10	15	14	13	14	16	17	16	15	64.9	32
Q6IRU2	Tropomyosin alpha-4 chain	*Tpm4*	37	28	6	6	8	5	8	5	6	10	10	7	77	28
Q7TQD2	Tubulin polymerization-promoting protein	*Tppp*	10	10	2	0	4	2	3	4	4	3	2	4	49.1	24
Q9CRB6;Q0P5Y3	Tubulin polymerization-promoting protein family member 3	*Tppp3*	16	16	6	3	5	5	4	9	7	10	8	9	76.1	19
P63028;D3YU75	Translationally-controlled tumor protein	*Tpt1*	9	9	1	2	1	3	5	4	5	4	4	4	41.3	19
Q8R3G9	Tetraspanin-8	*Tspan8*	3	3	1	1	1	1	0	2	2	2	2	2	10.2	26
P52196	Thiosulfate sulfurtransferase	*Tst*	12	12	1	1	4	1	5	5	6	6	3	3	43.1	33
P23591	GDP-L-fucose synthase	*Tsta3*	12	12	1	2	5	4	4	5	5	6	7	6	35.2	36
E9PY03	0	*Tstd1*	4	4	0	0	1	0	1	1	2	1	1	2	26.3	14
A3KMP2;A3KMP2-2;D6RH59	Tetratricopeptide repeat protein 38	*Ttc38*	17	17	1	0	1	1	2	7	7	8	8	8	38.9	52
P07309	Transthyretin	*Ttr*	8	8	2	2	3	2	0	3	4	4	3	3	39.5	16
P68368;Q9JJZ2;Q3UX10	Tubulin alpha-4A chain	*Tuba4a*	24	6	1	1	1	1	2	1	2	2	2	2	48.2	50
P68372;J3QNR5;Q9D6F9	Tubulin beta-4B chain;Tubulin beta-4A chain	*Tubb4b;Tubb4b-ps1;Tubb4a*	29	6	2	3	3	4	2	5	5	5	5	5	61.3	50
P99024;A2AQ07;CON__ENSEMBL:ENSBTAP00000025008;G3UZR1	Tubulin beta-5 chain	*Tubb5*	26	4	0	1	1	1	1	1	0	3	3	1	53.4	50
Q8BFR5;D3YVN7;Q8BFR5-2	Elongation factor Tu, mitochondrial;Elongation factor Tu	*Tufm;Gm9755*	29	29	7	9	8	7	12	14	14	11	11	14	55.5	50
Q91YR1;D3Z2H0	Twinfilin-1	*Twf1*	21	18	5	3	6	5	6	6	6	8	6	7	56	40
P10639	Thioredoxin	*Txn*	9	9	3	4	3	3	4	3	5	4	3	3	55.2	12
Q9CQM5	Thioredoxin domain-containing protein 17	*Txndc17*	9	9	2	1	2	3	3	3	3	3	3	2	72.4	14
E9PXX7;Q91W90	Thioredoxin domain-containing protein 5	*Txndc5*	13	13	1	1	1	0	5	4	4	5	6	5	39.2	39
Q8CDN6	Thioredoxin-like protein 1	*Txnl1*	10	10	0	2	1	3	5	3	5	6	5	3	35.3	32
Q9JMH6-2;Q9JMH6	Thioredoxin reductase 1, cytoplasmic	*Txnrd1*	16	16	2	1	3	4	4	4	5	4	4	3	31.1	55

P; Peptides, UP; Unique peptides, SC; Sequence coverage, MW: Molecular weight

Q91YN5-3;Q91YN5;Q3UHZ7;Q91YN5-2	UDP-N-acetylhexosamine pyrophosphorylase;UDP-N-acetylgalactosamine pyrophosphorylase;UDP-N-acetylglucosamine pyrophosphorylase	*Uap1*	19	18	0	3	5	2	6	4	5	4	4	4	39	59
Q02053;P31254	Ubiquitin-like modifier-activating enzyme 1	*Uba1*	41	41	0	1	8	6	14	14	16	17	14	13	35.9	118
Q9Z1F9;H3BJQ2;A2BH29;H3BLM2;H3BLR3	SUMO-activating enzyme subunit 2	*Uba2*	11	11	0	0	2	2	3	3	2	5	4	3	19.9	71
P61089;Q9CQ37	Ubiquitin-conjugating enzyme E2 N	*Ube2n*	8	8	1	3	4	2	2	3	4	4	5	2	52.6	17
Q9CZY3;Q9CZY3-2;D3Z1I3;B7ZBY7;E9PY39;D3YXL3;B7ZBY6;B2KF55;D3Z6R2	Ubiquitin-conjugating enzyme E2 variant 1	*Ube2v1;Gm8325;Gm20431;Gm5830*	7	3	0	0	1	1	2	0	1	1	1	0	43.5	16
Q8R317-2;Q8R317	Ubiquilin-1	*Ubqln1*	7	5	0	0	0	1	0	0	2	3	3	2	14.3	59
Q9JKB1;P58321	Ubiquitin carboxyl-terminal hydrolase isozyme L3	*Uchl3*	8	8	0	1	1	3	4	3	2	4	3	3	37.4	26
O70475;D3Z3F7;D3YXP9;F6ZQT4	UDP-glucose 6-dehydrogenase	*Ugdh*	28	28	8	6	12	9	11	9	15	15	11	11	48.3	55
Q91ZJ5;Q91ZJ5-2	UTP--glucose-1-phosphate uridylyltransferase	*Ugp2*	25	25	3	4	8	10	13	9	8	9	11	9	40.4	57
Q9CQB4;Q9D855	Cytochrome b-c1 complex subunit 7	*Uqcrb*	13	13	1	1	4	2	0	5	7	8	5	7	58.6	14
Q9CZ13;Q9CXT8	Cytochrome b-c1 complex subunit 1, mitochondrial	*Uqcrc1*	18	18	7	6	8	7	7	10	11	12	10	10	32.9	53
Q9DB77	Cytochrome b-c1 complex subunit 2, mitochondrial	*Uqcrc2*	24	24	9	6	10	7	9	18	15	14	15	12	51.7	48
Q9CR68	Cytochrome b-c1 complex subunit Rieske, mitochondrial;Cytochrome b-c1 complex subunit 11	*Uqcrfs1*	13	13	3	2	5	7	6	7	4	8	8	6	38.7	29
Q9CQ69;I7HPX6	Cytochrome b-c1 complex subunit 8	*Uqcrq*	9	9	2	1	3	2	2	2	3	3	3	2	59.8	10
H3BLC4;Q9ES64-3;Q9ES64-2;D6RIM8;E9PYX1;Q9ES64;E9QMN1;H3BIZ2	Harmonin	*Ush1c*	16	16	1	1	0	0	1	5	6	5	7	4	31.9	60
Q9Z1Z0;Q9Z1Z0-2;Q9Z1Z0-4;Q9Z1Z0-3	General vesicular transport factor p115	*Uso1*	15	15	0	0	3	1	3	2	2	4	5	0	18.2	107
E9PYI8;Q9JMA1	Ubiquitin carboxyl-terminal hydrolase;Ubiquitin carboxyl-terminal hydrolase 14	*Usp14*	11	11	2	1	1	1	2	2	2	6	4	3	26.6	52
Q3U4W8;P56399;D3YYA5;D3Z4K7	Ubiquitin carboxyl-terminal hydrolase;Ubiquitin carboxyl-terminal hydrolase 5	*Usp5*	23	23	2	2	3	4	4	4	3	7	7	2	26.7	93
Q9Z1Q9;G3UY93;G3UZ22;G3UYW2	Valine--tRNA ligase	*Vars*	21	21	0	0	1	0	3	7	3	9	9	8	18.8	140
P32921-2;P32921	Tryptophan--tRNA ligase, cytoplasmic;T1-TrpRS;T2-TrpRS	*Wars*	29	29	3	1	6	5	9	6	10	9	10	8	49.1	54
P70460	Vasodilator-stimulated phosphoprotein	*Vasp*	20	20	4	1	3	3	5	7	4	6	7	5	50.7	40
Q64727	Vinculin	*Vcl*	85	85	22	28	35	39	40	33	35	40	36	41	75.2	117
Q01853	Transitional endoplasmic reticulum ATPase	*Vcp*	69	69	11	11	15	14	18	22	20	21	21	20	59.7	89

P; Peptides, UP; Unique peptides, SC; Sequence coverage, MW: Molecular weight

Q60932-2;Q60932;F2Z471	Voltage-dependent anion-selective channel protein 1	*Vdac1*	19	19	1	4	9	5	6	12	11	15	9	9	69.3	31
G3UX26;Q60930;D3YZT5;D3YUN8	Voltage-dependent anion-selective channel protein 2	*Vdac2*	11	11	2	3	7	4	3	6	6	7	5	5	41	30
Q60931;J3QPE8;J3QMG3	Voltage-dependent anion-selective channel protein 3	*Vdac3;Vdac3-ps1*	9	9	1	1	2	1	2	6	3	5	2	2	35	31
O88342	WD repeat-containing protein 1	*Wdr1*	41	41	10	9	12	13	16	16	22	19	20	15	69.6	66
Q62468;F6V2H5;O88398	Villin-1	*Vil1*	67	67	26	23	28	30	26	28	31	30	30	28	66	93
P20152;P03995-2;P03995;A2AKJ2;P15331-3;P15331;G5E846;P15331-2;G3X981;D3YZ35;P46660;P08553;P08551	Vimentin	*Vim*	54	44	2	4	13	4	15	12	11	13	22	21	67.6	54
Q9EQH3	Vacuolar protein sorting-associated protein 35	*Vps35*	13	13	0	0	3	2	2	6	5	5	6	6	17.3	92
Q99KC8;Q8BGF0;F6TIL5;D3Z518	von Willebrand factor A domain-containing protein 5A	*Vwa5a*	19	19	0	2	3	2	4	5	7	6	7	4	23.8	87
Q6P1B1	Xaa-Pro aminopeptidase 1	*Xpnpep1*	9	9	0	0	2	1	3	2	3	2	3	2	16.2	70
Q91WQ3;A2A7S7;F6VXZ2	Tyrosine--tRNA ligase, cytoplasmic	*Yars*	30	30	0	1	8	6	13	7	8	7	7	5	47.7	59
P62960;A2BGG7	Nuclease-sensitive element-binding protein 1	*Ybx1*	15	9	0	0	1	0	1	6	3	6	3	3	56.2	36
Q9CQV8;Q9CQV8-2;A2A5N1	14-3-3 protein beta/alpha;14-3-3 protein beta/alpha, N-terminally processed	*Ywhab*	24	15	3	3	2	2	4	4	5	7	5	4	65	28
P62259;D6REF3;F6WA09	14-3-3 protein epsilon	*Ywhae*	22	19	3	4	5	6	6	12	12	12	11	12	73.3	29
P61982	14-3-3 protein gamma;14-3-3 protein gamma, N-terminally processed	*Ywhag*	22	13	2	2	3	1	5	4	6	6	5	4	62.8	28
P68510	14-3-3 protein eta	*Ywhah*	24	17	3	5	3	5	5	8	8	7	10	7	62.2	28
P68254-2;P68254;F6YY69;F6VW30	14-3-3 protein theta	*Ywhaq*	23	17	0	2	3	3	3	5	6	6	6	7	70.4	28
P63101;D3YXN6;D3YXF4;D3YW45	14-3-3 protein zeta/delta	*Ywhaz*	28	20	4	6	8	7	8	8	11	10	9	9	71.4	28
Q8K0C5	Zymogen granule membrane protein 16	*Zg16*	13	13	7	5	4	5	6	6	7	8	6	6	79.6	18

P; Peptides

UP; Unique peptides
SC; Sequence coverage
MW; Molecular weight

P; Peptides, UP; Unique peptides, SC; Sequence coverage, MW: Molecular weight
